# Cation controlled rotation in anionic pillar[5]arenes and its application for fluorescence switch

**DOI:** 10.1038/s41467-023-36131-w

**Published:** 2023-02-03

**Authors:** Hao Zheng, Lulu Fu, Ranran Wang, Jianmin Jiao, Yingying Song, Conghao Shi, Yuan Chen, Juli Jiang, Chen Lin, Jing Ma, Leyong Wang

**Affiliations:** 1 State Key Laboratory of Analytical Chemistry for Life Science, Jiangsu Key Laboratory of Advanced Organic Materials, School of Chemistry and Chemical Engineering, Nanjing University, 210023 Nanjing, China; 2 Ma’anShan High-Tech Research Institute of Nanjing University, Ma’anShan, 238200 China

**Keywords:** Molecular machines and motors, Supramolecular chemistry

## Abstract

Controlling molecular motion is one of hot topics in the field of chemistry. Molecular rotors have wide applications in building nanomachines and functional materials, due to their controllable rotations. Hence, the development of novel rotor systems, controlled by external stimuli, is desirable. Pillar[n]arenes, a class of macrocycles, have a unique planar chirality, in which two stable conformational isomers *p*R and *p*S would interconvert by oxygen-through-the-annulus rotations of their hydroquinone rings. We observe the differential kinetic traits of planar chirality transformation in sodium carboxylate pillar[5]arene (**WP5-Na**) and ammonium carboxylate pillar[5]arene (**WP5-NH**_**4**_), which inspire us to construct a promising rotary platform in anionic pillar[5]arenes (**WP5**) skeletons. Herein, we demonstrate the non-negligible effect of counter cations on rotational barriers of hydroquinone rings in **WP5**, which enables a cation grease/brake rotor system. Applications of this tunable rotor system as fluorescence switch and anti-counterfeiting ink are further explored.

## Abstract (web summary)

Controlling molecular motion is challenging. Here, the authors report the effect of counter cations on the rotational barriers of hydroquinone rings in a molecular rotor based on anionic pillar[5]arenes.

## Introduction

**Fig. 1:**
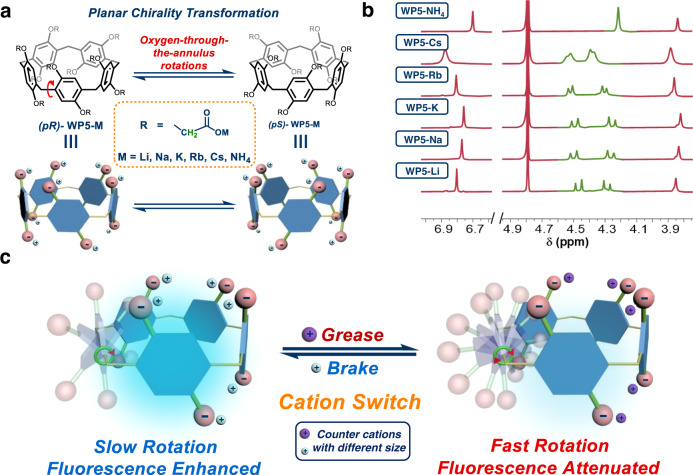
Overview of controllable rotor platforms based on WP5 skeletons. **a** Illustration of planar chirality transformation of **WP5-M**. **b** ^1^H NMR spectra of **WP5-M** in D_2_O (10.0 mM, 298 K), peaks of methylene groups are shown in green. **c** Cartoon Illustrations of cation-modulated switchable rotary motion of **WP5-M** (silver and purple balls represent different counter cations with distinct size). For simplification, only one rotary phenolic ring of **WP5** is shown.

Molecular rotors have various potential applications in molecular devices^1–3^, medicine^4–6^, asymmetric catalysis^7,8^, and smart materials^9–11^, which were derived from their controllable rotations. For example, light-driven molecular motors could drill through cell membranes using their molecular-scale actuation, inducing necrosis and facilitating chemical species into live cells^5^. Feringa and co-workers developed an artificial muscle-like functional materials which was based on supramolecular assembly of photoresponsive molecular motors^9^. Therefore, it is of great significance to develop novel rotor systems with controllable manners. As an emerging class of macrocycles, pillar[n]arenes have attracted wide attentions due to their electron-rich cavities, and played important roles in the field of supramolecular chemistry^12–16^. Pillar[n]arenes have a unique planar chirality, in which two stable conformations *p*R and *p*S could interconvert by oxygen-through-the-annulus rotations of their hydroquinone rings (Fig. 1a)^17–20^. Hence, pillar[n]arenes could be considered as promising rotor platforms.

Recently, a variety of strategies have been established to lock the planar chirality of pillar[n]arenes, such as guest^21–24^, solvent^25–27^, temperature^28–30^, as well as redox^31^. However, these strategies were based on thermodynamic control, while the research on kinetic control of transformation was rarely reported. Steric hindrance and intramolecular hydrogen bonds have been found crucial impacts on rotational barriers of hydroquinone units in pillar[5]arenes, which were reported by Ogoshi and Stoddart, respectively^32,33^. Nevertheless, the kinetic control on rotations in pillar[n]arenes, and in particular, the behavior of switchable rotary motions of them was unexplored so far.

Sodium carboxylate pillar[5]arene (**WP5-Na**) and ammonium carboxylate pillar[5]arene (**WP5-NH**_**4**_) (Fig. 1a), are widely used in aqueous supramolecular assembly systems^34–37^. Hitherto, the effect of counter cations on **WP5** had never been reported. According to the reported ^1^H NMR spectra of **WP5-Na** and **WP5-NH**_**4**_ in D_2_O^34–37^, we serendipitously noticed that the signal of methylene group in the rims of **WP5-Na** was split into two doublets, while in **WP5-NH**_**4**_ case it was a singlet (marked in green, Fig. 1b), indicating slower rotations of hydroquinone moieties in **WP5-Na** and faster rotations in **WP5-NH**_**4**_^38^. We supposed such distinct kinetic properties may attribute to impacts of counterions Na^+^ and NH_4_^+^ on rotational barriers of hydroquinone rings in **WP5**. This discovery provided us possibilities to construct switchable rotations in **WP5** by means of cation switch. In addition, it is worth noting that one of the working mechanisms of the aggregation-induced emission (AIE) phenomena was the restriction of intramolecular rotations (RIR, e.g., tetraphenylethylene derivatives), of which the aggregation could reduce the speed of intramolecular rotations, lowering energy dissipations, and thus the fluorescence intensity could be enhanced^11^. Moreover, pillar[n]arenes have been recently reported as AIEgens (luminogens exhibiting AIE attributes), and their AIE properties were arisen from restricted rotations of their phenolic rings^39,40^. Inspired by that, we inferred controlling rotary speed of hydroquinone rings in **WP5** would thus result in fluorescence switch.

In this work, we investigate the effect of various counterions on rotations of hydroquinone rings in **WP5**, establishing a controllable rotary system, and its applications in fluorescence switch and anti-counterfeiting inks are further developed (Fig. 1c and Supplementary Movie 1).

## Results

### Design and synthesis

**Fig. 2:**
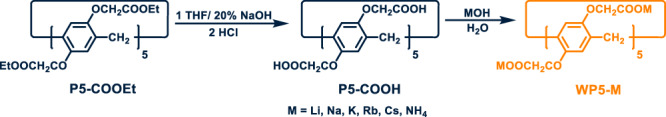
General synthetic routes of WP5-M. **WP5-M** was obtained by reacting the hydroxide of corresponding cations (MOH) with **P5-COOH**, the hydrolysis product of **P5-COOEt**.

To explore the impact of counterions on rotations of hydroquinone units in anionic pillar[5]arenes, carboxylate pillar[5]arenes with various monovalent counterions were chosen (**WP5-M**, Fig. 1a), which could be completely ionized in water. In this work, we focused on alkali metal ions (i.e., Li^+^, Na^+^, K^+^, Rb^+^, Cs^+^), and ammonium ion (NH_4_^+^) was also included (Fig. 1a). These anionic pillar[5]arenes were facilely synthesized by reacting the carboxylic-substituted pillar[5]arene (**P5-COOH**) with the hydroxide of corresponding cations (Fig. 2 and Supplementary Figs. 2–11). Comparing the ^1^H NMR spectra of **WP5-M** in D_2_O at 298 K, it was found that methylene peaks in the rims of **WP5-Li**, **WP5-Na**, **WP5-K**, **WP5-Rb**, and **WP5-Cs** were split into two doublets, indicating their non-equivalency (i.e., slow rotations), while **WP5-NH**_**4**_ exhibited a singlet peak, referring to the fastest rotation among **WP5** derivativities (Fig. 1b).

### Variable-Temperature NMR studies

**Fig. 3:**
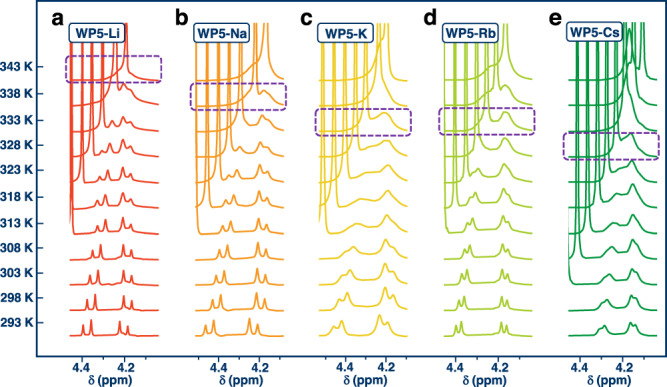
Partial VT NMR spectra of WP5-M in D_2_O. The coalesce temperature for **WP5-Li** (**a**), **WP5-Na** (**b**), **WP5-K** (**c**), **WP5-Rb** (**d**), and **WP5-Cs** (**e**) was 343 K, 338 K, 333 K, 333 K, and 328 K, respectively (marked with purple dashed). The concentration of rotors was 10.0 mM.

**Fig. 4:**
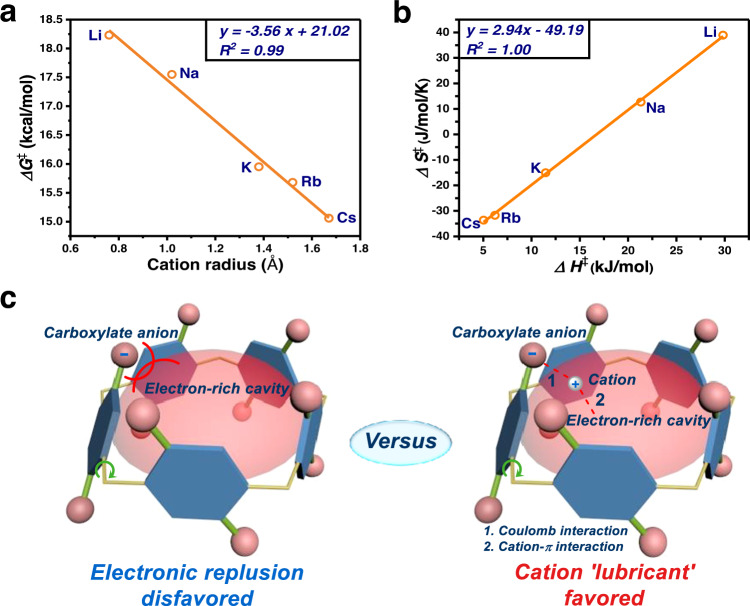
Kinetic analysis of rotations in WP5-M. **a** Correlation of the rotational barrier *ΔG*^*≠*^_*exp*_ with corresponding cation radius. **b** Enthalpy-entropy compensation plot of **WP5-M**. **c** Illustration of electron repulsion between carboxylate anion (represented with pink balls) and electron-rich cavity (represented with red translucent hemisphere) versus cation (represented with silver balls) lubricant effect.

For quantitatively examining rotational barriers of rotors, **WP5-M** were then subjected to variable temperature (VT) NMR studies^32,33,38,41,42^. The two doublet methylene peaks tended to coalesce at 343 K, 338 K, 333 K, 333 K, and 328 K for **WP5-Li**, **WP5-Na**, **WP5-K**, **WP5-Rb**, **WP5-Cs** in D_2_O, respectively (Fig. 3). A rotational barrier (*ΔG*^*≠*^) of 18.23, 17.55, 15.95, 15.68, and 15.06 kcal/mol was revealed based on Eyring plots^38^, of which the accuracy was validated by three independent measurements (Table 1 and Supplementary Figs. 12–21). This result suggested that counter cations of **WP5-M** affected rotational barriers of hydroquinone rings in **WP5-M** indeed. Interestingly, a linear line was obtained when we tried to correlate the experimental rotational barriers with the radius r of corresponding cations (*R*^2^ = 0.99, Fig. 4a). It decreases in rotational barrier *ΔG*^*≠*^ as the ionic radius increases (negative slope). Li^+^, with a smallest radius of 0.76 Å, gave the largest *ΔG*^*≠*^ (i.e., slowest rotation speed). Although it was a failure to measure the rotational barrier of **WP5-NH**_**4**_ owing to the limitation of the freezing point of deuteroxide, a qualitative result could be drawn that **WP5-NH**_**4**_ possessed the lowest rotational barrier.

**Table 1:** The summery of kinetic parameters of WP5-M in D_2_O

Rotors^a^	*ΔH*^*≠*^ (kcal mol^−1^)	*ΔS*^*≠*^ (cal mol^−1^ K^−1^)	*k*_298 K_^b^ (s^−1^)	*ΔG*^*≠*^_298 K_ (kcal mol^−1^)
**WP5-Li**	29.83	38.92	0.26	18.23
**WP5-Na**	21.32	12.67	0.82	17.55
**WP5-K**	11.47	−15.04	13.04	15.95
**WP5-Rb**	6.22	−31.74	18.03	15.68
**WP5-Cs**	5.04	−33.65	53.47	15.06

^a^The concentration of rotors was 10.0 mM.

^b^Rotational speed was revealed based on Eyring Plot.

We inferred that the decreasing tendency of rotational barriers of **WP5-M** could be ascribed to energy difference of ground states (GS) or transition states (TS), which was induced by counter cations of **WP5-M** (Supplementary Fig. 22). The impact of GS energy on rotational barriers should be minor, since NH_4_^+^ possessed stronger stability to **WP5** anion than K^+^ (contribution of hydrogen bonding) in ground states, but the rotational barrier of **WP5-NH**_**4**_ was much lower than that of **WP5-K** (Supplementary Fig. 23). Besides, a linear relationship between experimental *ΔS*^*≠*^ and *ΔH*^*≠*^ was observed, which was a typical enthalpy-entropy compensation (*R*^2^ = 1.00, Fig. 4b)^43^. It suggested the existence of enhanced binding force (enthalpy favored) in TS from **WP5-Li** to **WP5-Cs**, which would lead to reduction of configurational freedom (entropy decrease). Therefore, we speculated that this downward trend of rotational barriers could be interpreted as following: in rotary process, there was a strong electronic repulsion between carboxylate anions in the rims and the electron-rich cavity of pillar[5]arenes (Fig. 4c). Cation acting as grease, could insert between anions and cavities, involving coulomb interactions with anions and cation-π interactions with cavities, by which the disfavored repulsion could be impaired. Ascribing to a size-specific mechanism that was ubiquitous in macrocyclic host-guest systems (e.g., the binding constant of 18-crown-6 with K^+^ was higher than that with Na^+^ and the size of K^+^ that was more matched with the cavity of 18-crown-6)^44–46^, larger counter cations might have stronger interactions, leading to an enhanced lubricant effect (Fig. 4c). Furthermore, although radii of NH_4_^+^ and K^+^ were similar (1.43 Å and 1.38 Å, respectively), **WP5-NH**_**4**_ had a lowest rotational barrier, which could be attributed to additional hydrogen bond between ammonium ion and carboxylate oxygen. To preliminarily validate this size-specific mechanism in pillar[5]arenes, we measured binding constants (*K*_*a*_) of M^+^ with ethoxycarbonylmethoxy-substituted pillar[5]arene (**P5-COOEt**, Fig. 2) in DMF/H_2_O solution (v/v = 4/1) using titration method (Supplementary Fig. 26)^47,48^. Although it was failed to measure *K*_*a*_ of Li^+^ and Na^+^ due to the fact that the changes of their UV absorption were too small to conduct the non-linear curve-fitting, an increased *K*_*a*_ of K^+^, Rb^+^, and NH_4_^+^ was successfully estimated to be 4.2 (± 1.3) × 10^2^, 7.9 (± 1.2) × 10^2^, and 1.1 (± 0.1) × 10^3^ M^−1^ respectively, indicating an enhanced binding force between cations and cavities of pillararenes, which was consistent with our hypothesis.

**P5-COOH**, the precursor of **WP5-M**, was almost insoluble in aqueous solution, and the ionization of its carboxyl hydrogen is incomplete. Notably, Ogoshi reported that the rotational barrier of **P5-COOH** in DMF-*d*_*6*_ was 17.08 kcal/mol^−1^ ^49^, which was lower than **WP5-Li** and **WP5-Na** but higher than **WP5-K**, **WP5-Rb** and **WP5-Cs**. This may be ascribed to the steric favor to hydrogen than Li^+^ and Na^+^, although Li^+^ and Na^+^ had modest electronic favor in rotation process. For K^+^, Rb^+^ and Cs^+^, the stabilizing effect brought by electronic effect would overcome the unfavorable steric hindrance. VT-NMR study on **P5-COOH** in a mixed solvent of DMF-*d*_*6*_/H_2_O (v/v = 3/1, Supplementary Figs. 24−25) was performed. The addition of water in organic solvent would facilitate ionization of carboxyl group (e.g., p*K*_*a*_ value of acetic acid in water and DMSO were 4.76 and 12.60 respectively)^50^, so the electronic effect during rotations of **P5-COOH** in the above mixed solvent could be amplified. A rotational barrier of 18.37 kcal/mol^−1^ was revealed for **P5-COOH**, which was the highest energy barrier among **WP5** derivatives. This result suggested that if the carboxyl group could be fully ionized, the lubricant effect of the proton could be very weak owing to its smallest size compared with metal cations.

### Theoretical computations

**Fig. 5:**
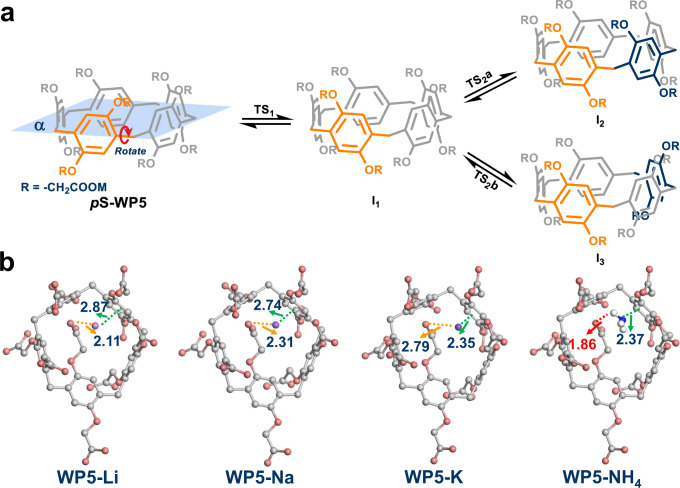
Mechanism studies of rotation process of WP5-M. **a** Partial pathways of transformation between its *p*S and *p*R conformers of **WP5-M**. **b** Configurations extracted from potential energy surface scanning when hydroquinone ring rotated to the plane α. The dashline represents the distance between cations and corresponding functional groups. The non-bonded distances are in units of Å.

Theoretical calculations were next carried out for validating our above-mentioned hypothesis. To begin with, the binding energy between cations and GS structures of **WP5** anion (*p*S conformation) was calculated (E_GS_, Supplementary Table 1). E_GS_ fell off in the sequence of NH_4_^+^ > Li^+^ > Na^+^ > K^+^ (i.e., the stability sequence of GS: **WP5-NH**_**4**_ > **WP5-Li** > **WP5-Na** > **WP5-K**), which was inconsistent to experimental results. Hence, the difference among GS energy of **WP5-M** had minor effect on their discrepant rotational barriers. Then, we focused on deciphering transition states of **WP5-M** during rotations. Considering that the actual transition states of **WP5-M** may be highly distorted and hardly calculated^51^, Stoddart’s TS model was employed, where the transformation between *p*S and *p*R conformers of **WP5-M** could be dissected into four plausible pathways with serval possible intermediates (Fig. 5a and Supplementary Fig. 27)^33^. The potential energy surface scanning (PESS) was subsequently performed to monitor energy variation during rotations (Supplementary Fig. 28)^52,53^. The stage TS_1_ was examined primarily (Fig. 5a), in which only one phenolic ring (marked in orange) was allowed free rotation around the cross-section of **WP5** (plane α, Fig.5a). Due to the complexity of structures of **WP5** derivatives, it was difficult to locate transition state structures in rotation process. Instead, configurations with the highest energy sampled from PESS were considered as preliminary transition state structures for estimating rotational barriers (Supplementary Fig. 29)^54–56^. The calculation results were presented in Supplementary Figs. 30–33 and Supplementary Table 2, from which **WP5-Li** was found to have the highest rotational barrier of 8.83 kcal/mol for TS_1_. Additionally, the binding energy between cations and TS_1_ structures of **WP5** anion was also computed (E_TS_, Supplementary Table 1). For the same cation, E_TS_ was higher than its E_GS_, which showcased stronger impact of cations on transition states than that on ground states.

To further understand rotation processes of **WP5-M**, the energy barrier for TS_2_ was also evaluated. In this step, hydroquinone rings in ortho- (*o*-, TS_2_a) or meta- (*m*-, TS_2_b) position of initially rotated unit flipped around plane α (marked in blue, Fig. 5a). It was found that rotational barriers of TS_2_a were higher than TS_2_b in all studied systems (e.g., TS_2_a: 16.18 kcal/mol; TS_2_b: 13.84 kcal/mol for **WP5-Li**, Supplementary Figs. 30–33, Supplementary Table 2), which may be ascribed to steric hinderance of two neighboring hydroquinone rings in TS_2_a. Thus, TS_2_b was a favored pathway when the second hydroquinone ring tended to rotate. The highest energy barriers among two rotational processes (TS_1_ and TS_2_b) were evidently reduced from **WP5-Li**, **WP5-Na**, **WP5-K**, to **WP5-NH**_**4**_, which was in accordance with experimental results. Sampled structures from PESS were shown in Fig. 5b, in which hydroquinone rings rotated to the plane α for **WP5-Li**, **WP5-Na**, **WP5-K**, and **WP5-NH**_**4**_, respectively. A decrease in the non-bonded distance between cations and vertical phenolic rings in *m*- position (**WP5-Li**: 2.87 Å; **WP5-Na**: 2.74 Å; **WP5-K**: 2.35 Å; and **WP5-NH**_**4**_: 2.37 Å) indicated the enhanced cation-π interactions (Fig. 5b). Meanwhile, hydrogen bonding between ammonium ion and carboxylate oxygen of **WP5-NH**_**4**_ was significant, which could be contributed to the lowest rotational barrier of **WP5-NH**_**4**_ among these anionic pillar[5]arenes (Fig. 5b).

### Solvent effects

Considering cations are highly solvated in water, and solvation might get involved in the rotation process of **WP5-M**, **WP5-Na** was chosen as the model to explore the effect of ionic solvation on rotations. We performed additional VT NMR studies in mixed solvent (D_2_O mixed with polar protic solvent methanol-*d*_*4*_, or polar aprotic solvent DMSO-*d*_*6*_), due to insolubility of **WP5-M** in nonaqueous solvent. Rotational barriers and relevant kinetic parameters of **WP5-Na** in the mixed solvent were equivalent to that in deuterium oxide (Supplementary Table 3 and Supplementary Figs. 34–43). This result indicated that the ionic solvation had little effect on rotation process, which may be ascribed to narrow cavities of **WP5** limited the solvation of cations in transition states. Since solvents were irrelevant to the rotational barriers of **WP5-M**, we attempted to study kinetic traits of **WP5-NH**_**4**_ in a mixed solvent (D_2_O/ methanol-*d*_*4*_, v/v = 3/2, the maximum mixing ratio that be applied for dissolving **WP5-NH**_**4**_ with a 10.0 mM concentration), of which the freezing point was about 228 K. Although a coalesce temperature of 268 K was revealed for **WP5-NH**_**4**_, unfortunately, the lower temperature couldn’t lead the coalescent methylene peaks to fully split into two doublet (Supplementary Fig. 44). Hence, the rotational barrier of **WP5-NH**_**4**_ could not be calculated according to current VT NMR data.

### Cation grease/brake rotations and fluorescence switch

**Fig. 6:**
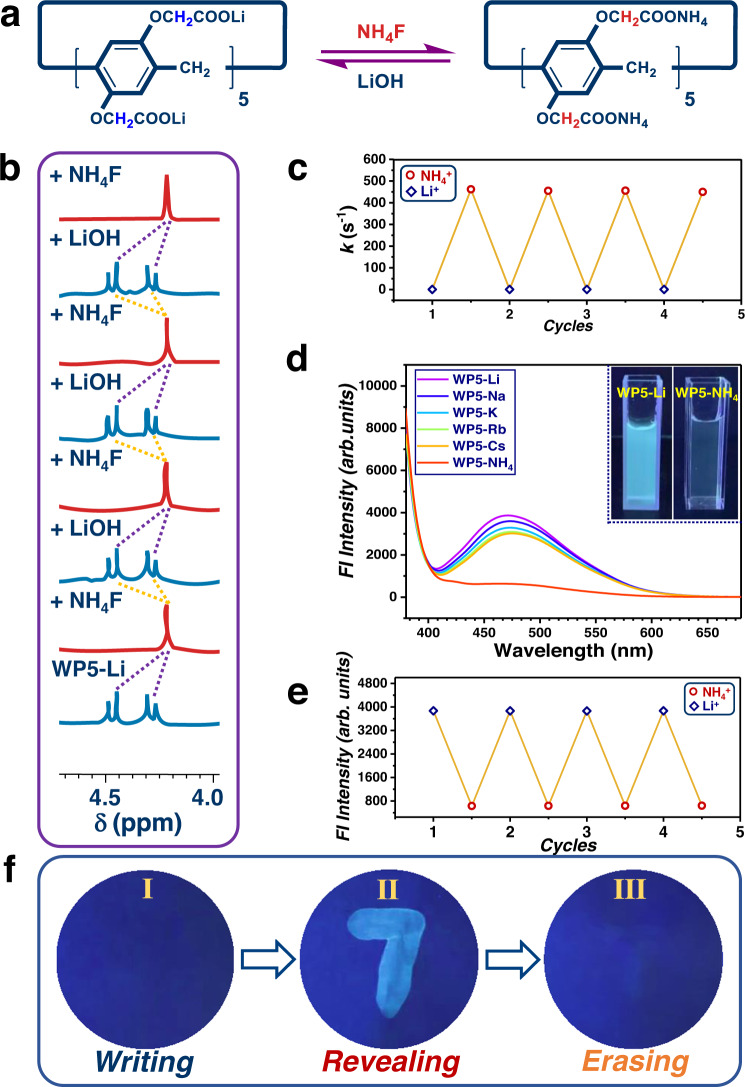
Rotational speed control and fluorescence switch of WP5-M. **a** Cation switch between Li^+^ and NH_4_^+^. **b** Partial NMR of switching control of rotors. **c** Switching of rate for **WP5** in D_2_O at 298 K with multiple cycles of cation exchange. **d** Fluorescence emission spectrum of **WP5-M** (*c* = 5.0 mM, *λ*_ex_ = 365 nm; insert: fluorescence of **WP5-Li** and **WP5-NH**_**4**_ under 365 nm UV irradiation). **e** Fluorescence response of **WP5** (5.0 mM) at 471 nm upon multiple cycles of cation switch between Li^+^ and NH_4_^+^ in D_2_O (*λ*_ex_ = 365 nm). **f** Revealing and erasing information with **WP5** inks: the image written with **WP5-NH**_**4**_ under 365 nm light (I), and the image could be observed upon adding LiOH on the pre-existing images (II), which could be erased by painting NH_4_F (III).

Having extensively explored the impact of cations on rotational barriers of **WP5**, attention was subsequently turned to construct switchable rotary motions in **WP5** platforms, where counter cations could grease/brake of rotors. NH_4_^+^ and Li^+^ were chosen for an exchange pair, between which the switch could be easily achieved (Fig. 6a). Two doublets methylene peaks of **WP5-Li** were observed in ^1^H NMR spectra (Fig. 6b), showing slow rotations of rotors. NH_4_F was added for depositing Li^+^ with F^-^, leading to an acceleration of rotors (Fig. 6b). Subsequently, the solution was treated with LiOH, heating and bubbling with argon to remove NH_3_, resulting in a recovery of slow rotations (Fig. 6b). More importantly, the switch of the rotational speed could recur with multiple cycles (Fig. 6c). Furthermore, an attempt of reversibly switching **WP5-NH**_**4**_ and **WP5-Na** was also achieved, in which Na^+^ could be captured with 15-crown-5, and NH_4_^+^ could be removed under heating and bubbling the solution with argon after basified by NaOH (Supplementary Figs. 45–46).

Encouraged by these findings, next we put our effort on modulating photochemical properties of **WP5**. Strikingly, a downward trend of fluorescence intensities of **WP5-M** under 365 nm irradiation was observed (Fig. 6d), which fall in line with the sequence of rotational barrier. **WP5-Li**, with the highest rotational barrier, had the strongest luminescence, while s quite weak fluorescence was observed for **WP5-NH**_**4**_ (Fig. 6d). Presumably, restricted rotations reduced energy dissipations, and thus enhanced emission intensity, which was similar to RIR mechanisms of AIE phenomenon^11^. Furthermore, reversible fluorescence switching with several cycles was accomplished via the exchange of Li^+^ and NH_4_^+^ (Fig. 6e). This controllable fluorescence switch was promising to be considered in anti-counterfeiting technology (Fig. 6f). **WP5-NH**_**4**_ aqueous solution was utilized as inks, and a number “7” could be written on filter paper, which was invisible under 365 nm UV irradiation (Fig. 6f (I)). When applying LiOH aqueous solution on to the surface of pre-writing “7” image, the number could be revealed under 365 nm irradiation (Fig. 6f (II)). Moreover, the visible “7” image could be erased by painting NH_4_F solution onto the image surface again (Fig. 6f (III)).

## Discussion

In summary, the impact of cations on rotational barriers of anionic pillar[5]arenes has been investigated via dynamic NMR technology. It is found that cations had significant effects on rotational barriers of hydroquinone units in **WP5**, which is correlated with their radius. Next, theoretical computation is employed for providing mechanistic insights into rotation process. Due to orderly enhanced cation-π interactions between cations and electron-rich cavities in rotary processes, rotational barriers of **WP5-M** decrease from Li^+^ to Cs^+^. **WP5-NH**_**4**_ has the lowest rotational barrier among **WP5-M**, which is ascribed to additional hydrogen bonding. Finally, switchable rotary motions of rotors are explored. The accelera-tion/deacceleration of rotors is accomplished by means of cation exchange, and these findings further allow the fluorescence switch, which is applied as anti-counterfeiting inks. The strategies and results presented here should find potential applications in many fields, such as sensing, molecular devices, and smart materials.

## Methods

### Dynamic NMR studies

Rotational barriers of rotors **WP5-M** were measured via variable temperature (VT) ^1^H NMR by monitoring the broadening of diastereotopic methylene protons in the rims of **WP5-M**. VT ^1^H NMR spectra were recorded on a BRUKER AVANCE III 400 MHz or BRUKER AVANCE III 600 MHz spectrometer. The concentration of rotors were 10.0 mM. All VT experiments were performed three times. The NMR line shape analyses were performed on MestReNova 14 sofeware package (Mestrelab Research S. L.). Rates of exchange *k*_*ex*_ were obtained by line width analysis with the equation:

1 $${k}_{{ex}}={{{{{\rm{\pi }}}}}}({{{{{\rm{h}}}}}}-{{{{{{\rm{h}}}}}}}_{0})$$

where h represented the width of peak at half height, h_0_ represented the width of peak at slow or no exchange^38^.

Kinetic parameters were obtained by using the exchange rates (*k*_*ex*_, s^−1^) obtained from line width analysis of the VT ^1^H NMR spectra, the enthalpy change (*ΔH*^*≠*^) and entropy change (*ΔS*^*≠*^) of the transition state were calculated from Eyring plots:

2 $${{{{\mathrm{ln}}}}}\frac{k}{{{\mbox{T}}}}=-\frac{\varDelta {H}^{{{\ddagger}} }}{R}\frac{1}{{{\mbox{T}}}}+\frac{\varDelta {S}^{{{\ddagger}} }}{R}+{{{{\mathrm{ln}}}}}\left(\frac{{k}_{B}}{h}\right)$$

where k is the exchange rate constant, T is the absolute temperature, *ΔH*^*≠*^ is the enthalpy of activation, *R* is the universal gas constant, *k*_*B*_ is the Boltzmann constant, *h* is the Planck’s constant, and *ΔS*^*≠*^ is the entropy of activation. The free energy of activation (*ΔG*^*≠*^) was calculated through Gibbs equation:

3 $$\it {\Delta {{{{G}}}}}^{{{\ddagger}} }={\Delta {{{{H}}}}}^{{{\ddagger}} }-{{{{{{\rm{T}}}}}}\Delta {{{{S}}}}}^{{{\ddagger}} }$$

The relevant NMR spectra and Eyring Plots were avalilble at the Supplementary Information.

### Theoretical calculation

All computational calculations were carried out with Gaussian 16 software^57^. The potential energy surface have been scanned using semi-empirical PM6 method with dispersion correction (PM6-D3)^52,53^. The sampled configurations from the scanned structures were further optimized using the B3LYP-D3 functional^58–61^. The standard 6–31 G(d) and 6–31 G + (d) basis sets were used for nonmetal atoms and metal Li, Na and K, respectively. For detail, please see Supplementary Information and Supplementary Data 1.

## Supplementary information

Supplementary Information

Peer Review File

Description of Additional Supplementary Files

Supplementary Data 1

Supplementary Movie 1

## Data Availability

The authors declare that the all data supporting the findings of this study are available within this article and Supplementary Information files. The Supplementary Information file contains the experimental details and characterization of the compounds. Supplementary Data 1 contains the coordinates of the calculated structures. Supplementary Movie 1 is a demo of how rotations are controlled by cations in **WP5**.

## References

[1] Collins BSL, Kistemaker JCM, Otten E, Feringa BL (2016). A chemically powered unidirectional rotary molecular motor based on a palladium redox cycle. Nat. Chem..

[2] Kassem S (2017). Artificial molecular motors. Chem. Soc. Rev..

[3] Kottas GS, Clarke LI, Horinek D, Michl J (2005). Artificial molecular rotors. Chem. Rev..

[4] Dong J (2020). Self-assembly of highly stable zirconium(IV) coordination cages with aggregation induced emission molecular rotors for live-cell imaging. Angew. Chem. Int. Ed..

[5] Garcia-Lopez V (2017). Molecular machines open cell membranes. Nature.

[6] van Leeuwen T, Lubbe AS, Štacko P, Wezenberg SJ, Feringa BL (2017). Dynamic control of function by light-driven molecular motors. Nat. Rev. Chem..

[7] Wang J, Feringa BL (2011). Dynamic control of chiral space in a catalytic asymmetric reaction using a molecular motor. Science.

[8] Grill K, Dube H (2020). Supramolecular relay-control of organocatalysis with a hemithioindigo-based molecular motor. J. Am. Chem. Soc..

[9] Chen J (2018). Artificial muscle-like function from hierarchical supramolecular assembly of photoresponsive molecular motors. Nat. Chem..

[10] Jin M, Yamamoto S, Seki T, Ito H, Garcia-Garibay MA (2019). Anisotropic thermal expansion as the source of macroscopic and molecular scale motion in phosphorescent amphidynamic crystals. Angew. Chem. Int. Ed..

[11] Mei J, Leung NL, Kwok RT, Lam JW, Tang BZ (2015). Aggregation-induced emission: together we shine, united we soar!. Chem. Rev..

[12] Ogoshi T, Kanai S, Fujinami S, Yamagishi T, Nakamoto Y (2008). para-bridged symmetrical pillar[5]arenes: their lewis acid catalyzed synthesis and host-guest property. J. Am. Chem. Soc..

[13] Ogoshi T, Yamagishi TA, Nakamoto Y (2016). Pillar-shaped macrocyclic hosts pillar[n]arenes: new key players for supramolecular chemistry. Chem. Rev..

[14] Chen CF, Han Y (2018). Triptycene-derived macrocyclic arenes: from calixarenes to helicarenes. Acc. Chem. Res..

[15] Li YF, Li Z, Lin Q, Yang YW (2020). Functional supramolecular gels based on pillar[n]arene macrocycles. Nanoscale.

[16] Zhou J (2021). Supramolecular cancer nanotheranostics. Chem. Soc. Rev..

[17] Strutt NL (2012). Incorporation of an A1/A2-difunctionalized pillar[5]arene into a metal-organic framework. J. Am. Chem. Soc..

[18] Fa S, Kakuta T, Yamagishi T, Ogoshi T (2019). Conformation and planar chirality of pillar[n]arenes. Chem. Lett..

[19] Fa S (2021). Pre-regulation of the planar chirality of pillar[5]arenes for preparing discrete chiral nanotubes. Chem. Sci..

[20] Wang X, Jia F, Yang LP, Zhou H, Jiang W (2020). Conformationally adaptive macrocycles with flipping aromatic sidewalls. Chem. Soc. Rev..

[21] Ogoshi T, Masaki K, Shiga R, Kitajima K, Yamagishi T (2011). Planar-chiral macrocyclic host pillar[5]arene: no rotation of units and isolation of enantiomers by introducing bulky substituents. Org. Lett..

[22] Lee E (2018). S. pseu-do[1]catenane-type pillar[5]thiacrown whose planar chiral inversion is triggered by metal cation and controlled by anion. J. Am. Chem. Soc..

[23] Chen Y (2020). Competitive selection of conformation chirality of water-soluble pillar[5]arene induced by amino acid derivatives. Org. Lett..

[24] Zhu H (2020). Pillararene host-guest complexation induced chirality amplification: a new way to detect cryptochiral compounds. Angew. Chem. Int. Ed..

[25] Ogoshi T, Akutsu T, Yamafuji D, Aoki T, Yamagishi T (2013). Solvent- and achiral-guest-triggered chiral inversion in a planar chiral pseudo[1]catenane. Angew. Chem. Int. Ed..

[26] Yao J (2021). Pressure-driven, solvation-directed planar chirality switching of cyclophano-pillar[5]arenes (molecular universal joints). Chem. Sci..

[27] Ogoshi T, Shiga R, Yamagishi T, Nakamoto Y (2011). Planar-chiral pillar[5]arene: chiral switches induced by multiexternal stimulus of temperature, solvents, and addition of achiral guest molecule. J. Org. Chem..

[28] Yao J (2017). Temperature-driven planar chirality switching of a pillar[5]arene-based molecular universal joint. Angew. Chem. Int. Ed..

[29] Fa S, Egami K, Adachi K, Kato K, Ogoshi T (2020). Sequential chiral induction and regulator-assisted chiral memory of pillar[5]arenes. Angew. Chem. Int. Ed..

[30] Yao J (2021). Overtemperature-protection intelligent molecular chiroptical photoswitches. Nat. Commun..

[31] Xiao C (2020). Redox-triggered chirality switching and guest-capture/release with a pillar[6]arene-based molecular universal joint. Angew. Chem. Int. Ed..

[32] Ogoshi T (2010). Synthesis and conformational characteristics of alkyl-substituted pillar[5]arenes. J. Org. Chem..

[33] Strutt NL, Schneebeli ST, Stoddart JF (2013). Stereochemical inversion in difunctionalised pillar[5]arenes. Supramol. Chem..

[34] Hao M (2020). A supramolecular artificial light-harvesting system with two-step sequential energy transfer for photochemical catalysis. Angew. Chem. Int. Ed..

[35] Li H (2018). Smart self-assembled nanosystem based on water-soluble pillararene and rare-earth-doped upconversion nanoparticles for pH-responsive drug delivery. ACS Appl. Mater. Interfaces.

[36] Shao L (2020). Constructing adaptive photosensitizers via supramolecular modification based on pillararene host-guest interactions. Angew. Chem. Int. Ed..

[37] Shi B (2016). Nanoparticles with near-infrared emission enhanced by pillararene-based molecular recognition in water. J. Am. Chem. Soc..

[38] Abraham, R. J., Fisher, J., Loftus, P. *Application of NMR Spectroscopy. Introduction to NMR Spectroscopy* (John Wiley & Sons Ltd: New York, 1988).

[39] Chen J-F, Meng G, Zhu Q, Zhang S, Chen P (2019). Pillar[5]arenes: a new class of AIEgen macrocycles used for luminescence sensing of Fe^3+^ ions. J. Mater. Chem. C.

[40] Chen YY (2021). Pillararene-based AIEgens: research progress and appealing applications. Chem. Commun..

[41] Dial BE, Pellechia PJ, Smith MD, Shimizu KD (2012). Proton grease: an acid accelerated molecular rotor. J. Am. Chem. Soc..

[42] Wu Y (2018). A multistage rotational speed changing molecular rotor regulated by pH and metal cations. Nat. Commun..

[43] Liu L, Guo Q-X (2001). Isokinetic relationship, isoequilibrium relationship, and enthalpy-entropy compensation. Chem. Rev..

[44] Gokel GW, Leevy WM, Weber ME (2004). Crown ethers: sensors for ions and molecular scaffolds for materials and biological models. Chem. Rev..

[45] Shan P (2022). Progress in host-guest macrocycle/pesticide research: Recognition, detection, release and application. Coord. Chem. Rev..

[46] Nie H, Wei Z, Ni X-L, Liu Y (2022). Assembly and applications of macrocyclic-confinement-derived supramolecular organic luminescent emissions from cucurbiturils. Chem. Rev..

[47] Ashton PR (1996). Self-assembly, spectroscopic, and electrochemical properties of [n]rotaxanes. J. Am. Chem. Soc..

[48] Connors, K. A. *Binding Constants* (John Wiley & Sons, Inc: New York, 1987)

[49] Ogoshi T, Hashizume M, Yamagishi T, Nakamoto Y (2010). Synthesis, conformational and host-guest properties of water-soluble pillar[5]arene. Chem. Commun..

[50] Bordwell FG (1988). Equilibrium acidities in dimethyl sulfoxide solution. Acc. Chem. Res..

[51] Wang X (2019). Introduction of polar or nonpolar groups at the hydroquinone units can lead to the destruction of the columnar structure of Pillar[5]arenes. Comput. Theor. Chem..

[52] Jan Řezáč JF, Salahub D, Hobza P (2009). Semiempirical quantum chemical PM6 method augmented by dispersion and H-bonding correction terms reliably describes various types of noncovalent complexes. J. Chem. Theory Comput..

[53] Stewart JJ (2007). Optimization of parameters for semiempirical methods V: modification of NDDO approximations and application to 70 elements. J. Mol. Model..

[54] Guo X (2020). Tackling the activity and selectivity challenges of electrocatalysts toward the nitrogen reduction reaction via atomically dispersed biatom cata-lysts. J. Am. Chem. Soc..

[55] Li J (2020). Accelerated dinitrogen electroreduction to ammonia via interfacial polarization triggered by single-atom protrusions. Chem.

[56] Liu S (2019). Facilitating nitrogen accessibility to boron-rich covalent organic frameworks via electro-chemical excitation for efficient nitrogen fixation. Nat. Commun..

[57] Frisch, M. et al. *Gaussian 16* (Revision B.01, Gaussian, Inc., 2016).

[58] Becke AD (1993). Density-functional thermochemistry. III. The role of exact exchange. J. Chem. Phys..

[59] Lee C, Yang W, Parr RG (1988). Development of the Colle-Salvetti correlation-energy formula into a functional of the electron density. Phys. Rev. B..

[60] Grimme S, Antony J, Ehrlich S, Krieg H (2010). A consistent and accurate ab initio parametrization of density functional dispersion correction (DFT-D) for the 94 elements H-Pu. J. Chem. Phys..

[61] Grimme S, Ehrlich S, Goerigk L (2011). Effect of the damping function in dispersion corrected density functional theory. J. Comput. Chem..

