# Supplementary material for: Cation controlled rotation in anionic pillar[5]arenes and its application for fluorescence switch
Source: Nat Commun. 2023 Feb 3;14:590. doi: 10.1038/s41467-023-36131-w (PMC9898256; doi:10.1038/s41467-023-36131-w)
Supplement: Supplementary file 1 — Supplementary Information [file 41467_2023_36131_MOESM1_ESM.pdf]

Supplementary Information

Cation Controlled Rotation in Anionic Pillar[5]arenes and Its Application for Fluorescence Switch

Hao Zheng, ‡[1] Lulu Fu, ‡[1] Ranran Wang,[1] Jianmin Jiao,[1] Yingying Song,[1] Conghao Shi,[1] Yuan Chen,[1] Juli Jiang,*[1,2] Chen Lin,*[1] Jing Ma,*[1] and Leyong Wang[1]

[1] State Key Laboratory of Analytical Chemistry for Life Science, Jiangsu Key Laboratory of Advanced Organic Materials, School of Chemistry and Chemical Engineering, Nanjing University, 163 Xianlin Avenue, Nanjing 210023, China

[2] Ma'anShan High-Tech Research Institute of Nanjing University, Ma'anShan, 238200, China

Table of Contents

1. **Supplementary Methods……………………………………………….….3-9**

   (1). Synthesis and characterization…………………..……......…3-6

   (2). Dynamic NMR Studies…………………………………………...6-7

   (3). UV titration……………………………..……………………..…..7-8

   (4). Theoretical calculation…………………………………………..8

   (5). Solvent effect……………………………..…………………….…..8-9

   (6). Cation switch reactions…………………………………..……...9

   (7). Inks writing tests…………………………………….………..9

2. Supplementary Figures…………………………..……..…….10-33

3. Supplementary Tables……………………………………..…….33-34

4. Supplementary Reference……………………………………….34-35

1. Supplementary Methods

All reactions were performed in air atmosphere unless otherwise stated. Deuterium solvents were purchased from Aldrich. All other reagents were obtained from commercial sources and were used without further purification, unless indicated otherwise. All yields were given as isolated yields. ^{1}H NMR and ^{13}C NMR spectra were recorded on a BRUKER AVANCE III 400 MHz or BRUKER AVANCE III 600 MHz spectrometer, and the chemical shifts (δ) for ^{1}H NMR spectra, given in ppm, are referenced to the residual proton signal of the deuterated solvent. Mass spectra were recorded on a Bruker IMPACT-II spectrometer. The UV-vis spectra were recorded on a Shimadzu UV-1780 UV-Vis-NIR Spectrometer. The fluorescence emission spectra were recorded on a Hitachi F-7000 Fluorescence Spectrometer.

(1).Synthesis and Characterization

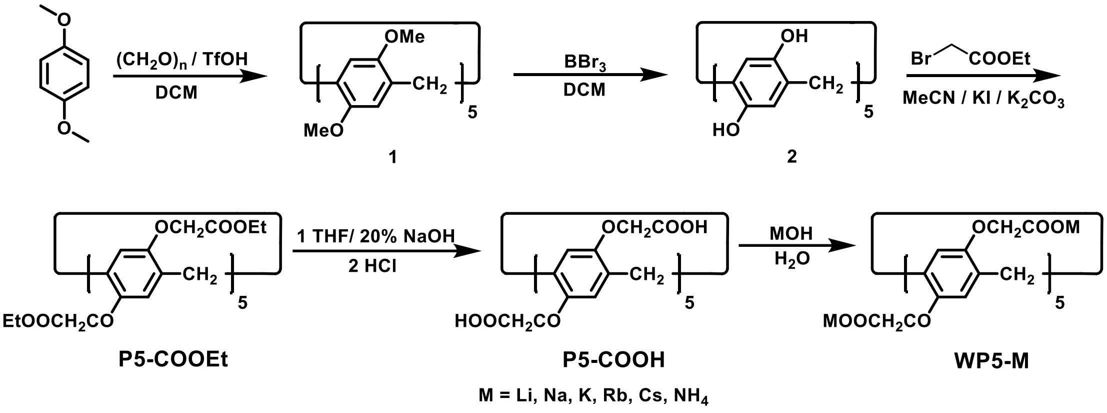

Supplementary Figure 1. General synthetic routes of **WP5-M**.

General procedure for synthesis of WP5-M: To a suspension of carboxylic pillar[5]arene (50 mg, 0.042 mmol, 1.0 equiv.) in H_2O (20 ml), was added MOH (0.42 mmol, 10.0 equiv.). The reaction was stirred at room temperature until affording a clear solution. After removal of solvent and dried under vacuo, **WP5-M** was obtained quantitatively as a dark yellow solid.

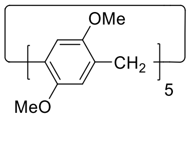

1: The reported procedure[1] was used: To a mixture of 1,4-

dimethoxybenzene (500 mg, 3.67 mmol) and paraformaldehyde (330.3 mg, 3.0 equiv.) in dry DCM, was added 5% TfOH. The reaction was stirred at room temperature for 5 h, and then quenched by the addition of a small amount of H_2O. The mixture was washed with H_2O, the organic phase was collected, concentrated under vacuo and then subjected to column chromatography (SiO_2: PE / EA, 10:1 to 6:1) to give the title compound **1** as white powder (275 mg, 50%). ^{1}H NMR (400 MHz, $CDCl_3$, 298 K): δ6.76 (s, 10H), 3.77 (s, 10H), 3.65 (s, 30H).

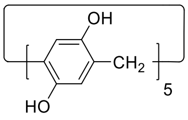

2: The reported procedure[1] was used: To a solution of **1** (150 mg, 0.2 mmol) in dry DCM (10 mL), boron tribromide (2.4 g, 9.7 mmol) was added in the condition of ice bath, and the mixture was stirred at room temperature for 96 h. Then, H_2O (20 mL) was added into the mixture, which was stirred for another 72 h. The precipitate formed was filtered and washed with water to afford title compound **2** as white powder (120 mg, 96%). ^{1}H NMR (400 MHz, CD_3COCD_3, 298 K): δ 7.95 (s, 10H), 6.48 (s, 10H), 3.57 (s, 10H).

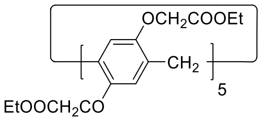

P5-COOEt: The reported procedure[1] was used: To a suspension of **2** (1.22 g, 2.0 mmol) in dry acetonitrile (60 ml), was added K_2CO_3. The mixture was stirred at room temperature for 30 min. 100 mg KI and excess of ethyl bromoacetate (5.0 ml, 45.0 mmol) were added. The reaction was refluxed under argon for 18 h. After the reaction was cooled down, the mixture was filtered. The filtrate was concentrated under vacuo, and the residue was subjected to column chromatography on silica gel (DCM / Me_2CO, 100/0 to 30/1) to afford the title compound as a brown solid (total yield: 23%). ^{1}H NMR (400 MHz, $CDCl_3$, 298 K): δ 7.05 (s, 10H), 4.54 (q, J = 15.0 Hz, 20H), 4.09 (m, J = 6.1 Hz, 20H), 3.86 (s, 10H), 0.98 (t, J = 6.0 Hz, 30H).

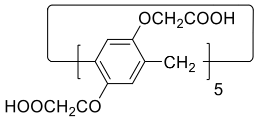

P5-COOH: The reported procedure[1] was used: To a solution of **P5-COOEt** (1.47 g, 1.0 mmol) in THF (60.0 ml), was added aqueous NaOH solution (30.0 ml, 20%). The reaction was refluxed for 15 h. The mixture was concentrated under vacuo, and the residue was diluted into 100 ml water. The solution was acidified with HCl, and the resulting precipitate was filtered off, washed with water, dried under vacuum to afford the title compound as a brown solid (total yield: 11%). ^{1}H NMR (400 MHz, DMSO-d_6, 298 K): δ 12.95 (br, 10H), 7.10 (s, 10H), 4.69 (d, J = 15.9 Hz, 10H), 4.41 (d, J = 15.8 Hz, 10H), 3.74 (s, 10H).

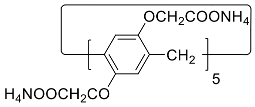

WP5-NH$_4$: The reported procedure[2] was used: To a suspension of **P5-COOH** (200.0 mg, 0.168 mmol, 1.0 equiv.) in H_2O (20.0 ml), was added $NH_3{\cdot}H_2O$ (0.3 ml, 20%). The reaction was stirred at room temperature until affording a clear solution. After removal of solvent and dried under vacuo, **WP5-NH$_4$** was obtained quantitatively as a dark yellow solid. ^{1}H NMR (400 MHz, D_2O, 298 K): δ 6.70 (s, 10H), 4.21 (s, 20H), 3.84 (s, 10H).

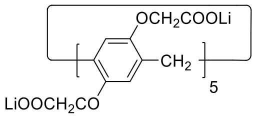

WP5-Li: The general procedure was used to quantitatively afford the title compound as a light brown solid. ^{1}H NMR (400 MHz, D_2O, 298 K): δ 6.80 (s, 10H), 4.47 (d, J = 15.9 Hz, 10H), 4.29 (d, J = 15.9 Hz, 10H), 3.85 (s, 10H). ^{13}C NMR (100 MHz, D_2O, 298 K) δ 177.44, 149.22, 128.68, 114.62, 67.74, 28.99. ESI-HRMS: m/z calcd for $C_{55}H_{40}O_{30}{}^{10-}$ $[M-10Li]^{10-}$: 118.0166; found: 118.0168.

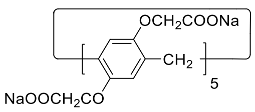

WP5-Na: The reported procedure[S1] was used: To a suspension of **P5-COOH** (200.0 mg, 0.168 mmol, 1.0 equiv.) in H_2O (20.0 ml), was added aqueous

NaOH solution (80.0 mg NaOH in 1 ml water). The reaction was stirred at room temperature until affording a clear solution. After removal of solvent and dried under vacuo, **WP5-Na** was obtained quantitatively as a dark yellow solid to quantitatively. ^{1}H NMR (400 MHz, D_2O, 298 K): δ 6.77 (s, 10H), 4.50 (d, *J* = 16.0 Hz, 10H), 4.26 (d, *J* = 16.1 Hz, 10H), 3.83 (s, 10H).

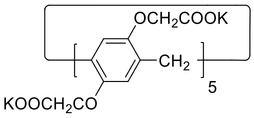

WP5-K: The general procedure was used to quantitatively afford the title compound as a light brown solid. ^{1}H NMR (400 MHz, D_2O, 298 K): δ 6.76 (s, 10H), 4.49 (d, *J* = 15.9 Hz, 10H), 4.26 (d, *J* = 16.1 Hz, 10H), 3.82 (s, 10H). ^{13}C NMR (100 MHz, D_2O, 298 K): δ 177.18, 149.10, 128.79, 114.47, 67.54, 28.83. ESI-HRMS: m/z calcd for $C_{55}H_{40}O_{30}^{10-}$ $[M-10K]^{10-}$: 118.0166; found: 118.0166.

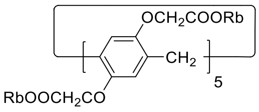

WP5-Rb: The general procedure was used to quantitatively afford the title compound as a light brown solid. ^{1}H NMR (400 MHz, D_2O, 298 K): δ 6.74 (s, 10H), 4.47 (d, *J* = 16.1 Hz, 10H), 4.24 (d, *J* = 16.1 Hz, 10H), 3.80 (s, 10H). ^{13}C NMR (100 MHz, D_2O, 298 K) δ 177.15, 149.32, 128.68, 114.87, 67.69, 29.19. ESI-HRMS: m/z calcd for $C_{55}H_{40}O_{30}^{10-}$ $[M-10Rb]^{10-}$: 118.0166; found: 118.0167.

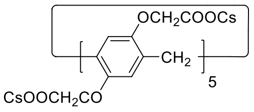

WP5-Cs: The general procedure was used to quantitatively afford the title compound as a light brown solid. ^{1}H NMR (400 MHz, D_2O, 298 K): δ 6.82 (s, 10H), 4.49 (d, *J* = 13.1 Hz, 10H), 4.33 (d, *J* = 14.7 Hz, 10H), 3.83 (s, 10H). ^{13}C NMR (100 MHz, D_2O, 298 K) δ 177.14, 149.05, 128.75, 114.43, 67.49, 28.78. ESI-HRMS: m/z calcd for $C_{55}H_{40}O_{30}^{10-}$ $[M-10K]^{10-}$: 118.0166; found: 118.0167.

(2). Dynamic NMR Studies

^{1}H NMR line shape analysis: Rotational barriers of rotors **WP5-M** were measured

via variable temperature (VT) ^{1}H NMR by monitoring the broadening of diastereotopic methylene protons in the rims of **WP5-M**. Rates of exchange k_{ex} were obtained by line width analysis with the equation:

$$k_{ex} = \pi(h - h_0) \qquad (1)$$

where h represented the width of peak at half height, h_0 represented the width of peak at slow or no exchange.[3] The concentration of rotors was 10.0 mM. All VT experiments were performed three times.

Kinetic parameters: By using the exchange rates (k_{ex}, s^{-1}) obtained from line width analysis of the VT ^{1}H NMR spectra, the enthalpy change ($\Delta H^‡$) and entropy change ($\Delta S^‡$) of the transition state were calculated from Eyring plots:

$$\ln\frac{k}{T} = -\frac{\Delta H^‡}{R}\frac{1}{T} + \frac{\Delta S^‡}{R} + \ln\left(\frac{k_B}{h}\right) \qquad (2)$$

where k is the exchange rate constant, T is the absolute temperature, $\Delta H^‡$ is the enthalpy of activation, R is the universal gas constant, k_B is the Boltzmann constant, h is the Planck's constant, and $\Delta S^‡$ is the entropy of activation. The free energy of activation ($\Delta G^‡$) was calculated through Gibbs equation:

$$\Delta G^‡ = \Delta H^‡ - T\Delta S^‡ \qquad (3)$$

(3). UV titration

The association constant of complex M^+ with **P5-COOEt** was determined by probing the charge-transfer band of the complex by UV/Vis spectroscopy and employing a titration method. Addition of MBF4 aqueous solution to a DMF/H2O solution (v/v = 4/1) with the same concentration (0.1 mM) of P5-COOEt resulted in an increase of the intensity of the CT band of the complex (**Supplementary Figure 26** left). Treatment of the collected absorbance data with a non-linear curve-fitting program afforded the corresponding association constant (K_a) of 4.2 (± 1.3) × 10^2, 7.9 (± 1.2) × 10^2, 1.1 (± 0.1) × 10^3 M^{-1} for K^+, Rb^+ and NH_4^+ respectively.

The non-linear curve-fitting was based on the equation:[4]

$$\Delta A = (A_\infty/[H]_0)\ (0.5[G]_0 + 0.5\ ([H]_0 + 1/K_a) - (0.5\ (([G]_0^2) + (2[G]_0(1/K_a - [H]_0)) + (1/K_a + [G]_0^2)^{0.5})) \qquad (4)$$

Where ΔA is the change of absorption intensity of **P5-COOEt** at 296 nm after addition of MBF_4 solution, A_∞ is the absorption intensity of the charge-transfer band when the host is completely complexed, $[H]_0$ is the fixed initial concentration of the host, and $[G]_0$ is the varying concentration of the guest.

(4). Theoretical calculation

All computational calculations were carried out with Gaussian 16 software.[5] The potential energy surface have been scanned using semi-empirical PM6 method with dispersion correction (PM6-D3).[6,7] The plane α was defined as original point (rotary part in horizontal position), where rotation to up and down corresponds to negative and position directions, respectively (**Figure S33a**). To give a further description of the relative position of cations in cavities of **WP5**, the angle between cation and two neighboring carbonyl oxygen atoms (θ) was also displayed (**Figure S33a**). The sampled configurations from the scanned structures were further optimized using the B3LYP-D3 functional.[8-11] The standard 6-31G(d) and 6-31G+(d) basis sets were used for nonmetal atoms and metal Li, Na and K, respectively.

The interaction energies are defined as:

$$E_{int} = E_{complex} - E_h - E_m \qquad (5)$$

where E_{int} is the interactions between **WP5** and different metal, E_{complex} is the total energy of the self-assembly formed by **WP5** and metal, E_{h} and E_{m} are the energy of **WP5** and different metals, respectively. The energies were calculated by the dispersion-corrected functional B3LYP-D3 with 6-31G(d) basis set for nonmetal atoms. The 6-31G+(d) basis sets were used for metal Li, Na and K, respectively.

(5). Solvent effect

Since the chemical shift of deuterium oxide in NMR spectrum could shift with the increase of temperature, the chemical shifts (δ) for VT ^{1}H NMR spectra in mixed solvent, given in ppm, are referenced to the proton signal of methanol-d_4 or DMSO-d_6. **WP5-Na** was chosen as the model compound to explore the effect of ionic solvation on rotational barriers of **WP5-M** in mixed solvent (D_2O mixed with polar protic solvent

methanol-d$_4$, or polar aprotic solvent DMSO-d_6). For further verifing our conclusion, VT studies of the remaining **WP5-M** in D_2O/methanol-d_4 were performed.

(6). Cation switch reactions

Rate of exchange k_{ex} of **WP5-NH4** at 298 K was estimated by line width analysis with the equation:

$$k_{ex} = \frac{\sqrt{2}\pi h}{2} \qquad (6)$$

where h represented the width of peak at half height.[3]

Li$^+$/NH$_4^+$ switching: To an aqueous solution of **WP5-Li** (10.0 mM, 3.0 ml, 1.0 equiv.), was added NH_4F (11.0 mg, 10.0 equiv.). After storing at 0 °C for 30 min, filtration of precipitate and characterization with ^{1}H NMR spectrum and fluorescence emission spectrum, LiOH (7.2 mg, 10.0 equiv.) was added. The mixture was heated to 60 °C and bubbled with Ar for 30 min, after which the solution was characterized with ^{1}H NMR spectrum and fluorescence emission spectrum.

Na$^+$/NH$_4^+$ switching: To an aqueous solution of **WP5-Na** (10.0 mM, 600 μl, 1.0 equiv.), was added 15-Crown-5 (24 μl, 20.0 equiv.) and NH_4Cl (3.2 mg, 10.0 equiv.). After characterizing with ^{1}H NMR spectrum, NaOH (2.4 mg, 10.0 equiv.) was added. The mixture was heated to 60 °C and bubbled with Ar for 30 min, after which the solution was characterized with ^{1}H NMR spectrum.

(7). Inks writing tests.

WP5-NH4 aqueous solution (10.0 mM) was employed as inks. Typically, inks were written on a filter paper and LiOH aqueous solution (10.0 mM) was used for revealing the preexisting images. The visible images could be erased by NH_4F aqueous solution (10.0 mM).

2. Supplementary Figures

(1). NMR spectrum of synthetic compounds

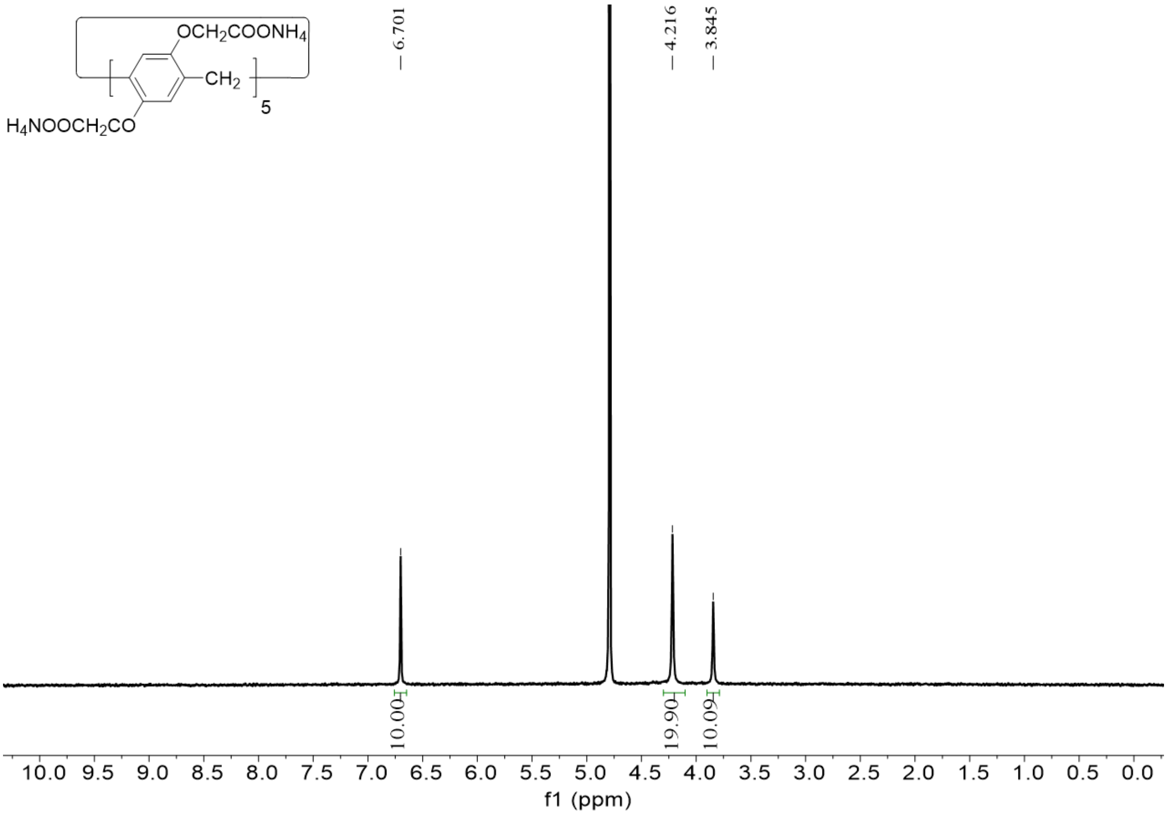

Supplementary Figure 2. ^{1}H NMR spectra of **WP5-NH4** in D_2O at 298 K.

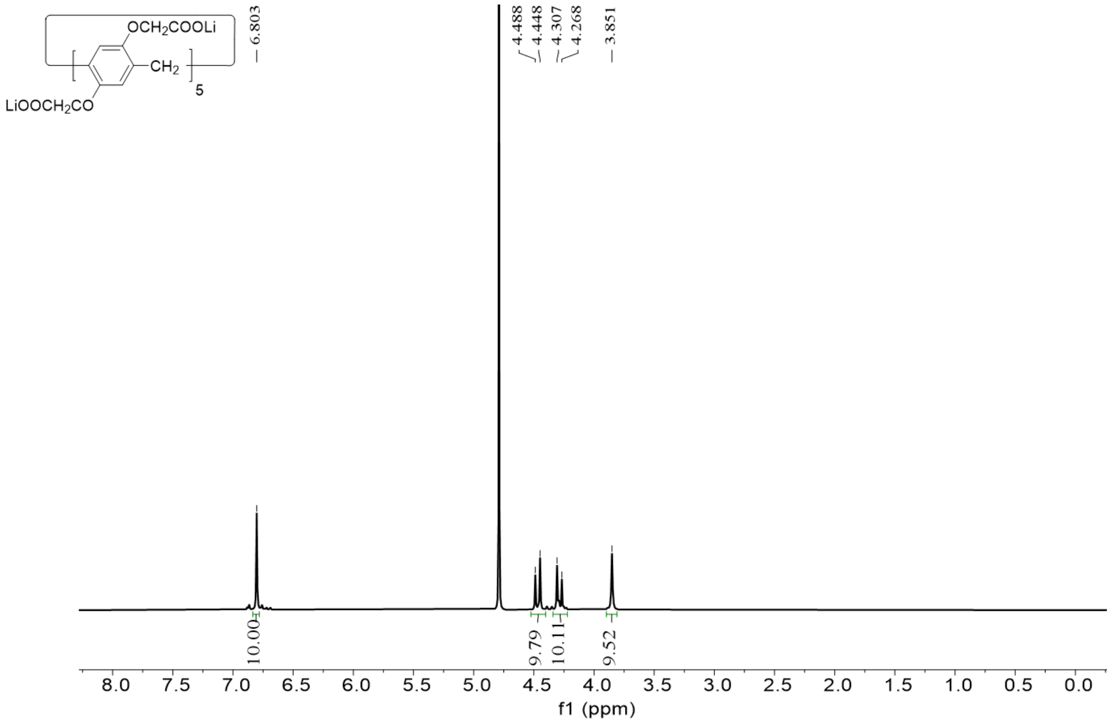

Supplementary Figure 3. ^{1}H NMR spectra of **WP5-Li** in D_2O at 298 K.

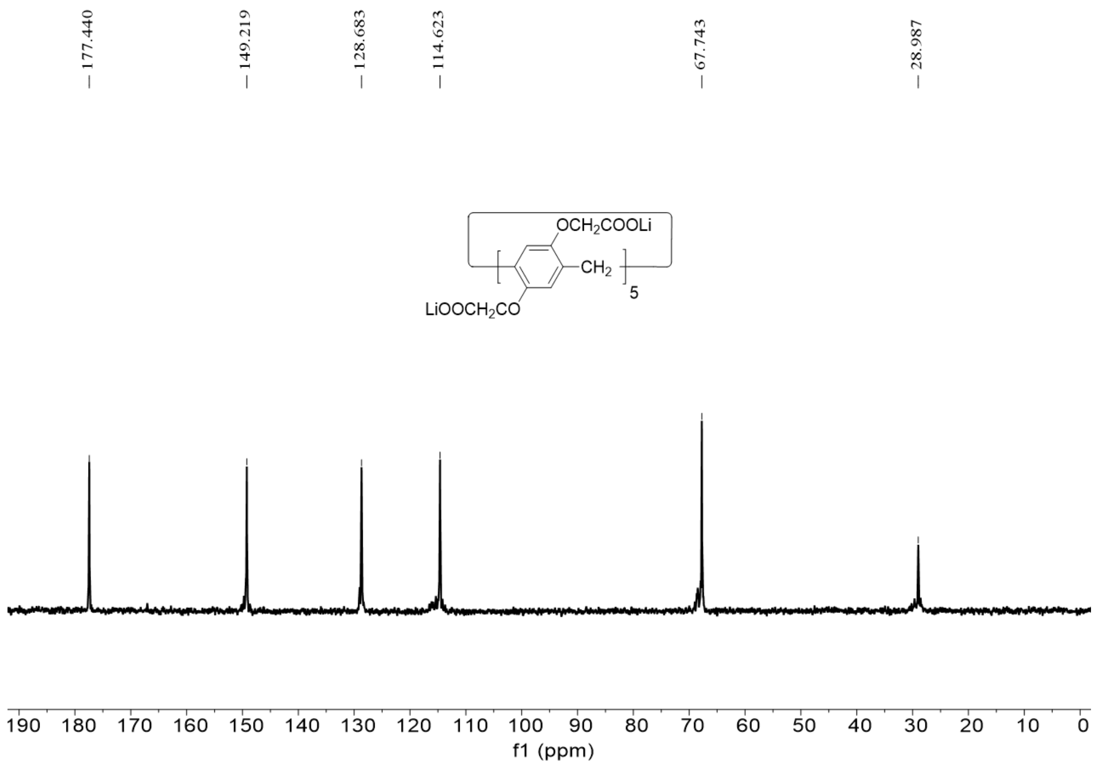

Supplementary Figure 4. ^{13}C NMR spectra of **WP5-Li** in D_2O at 298 K.

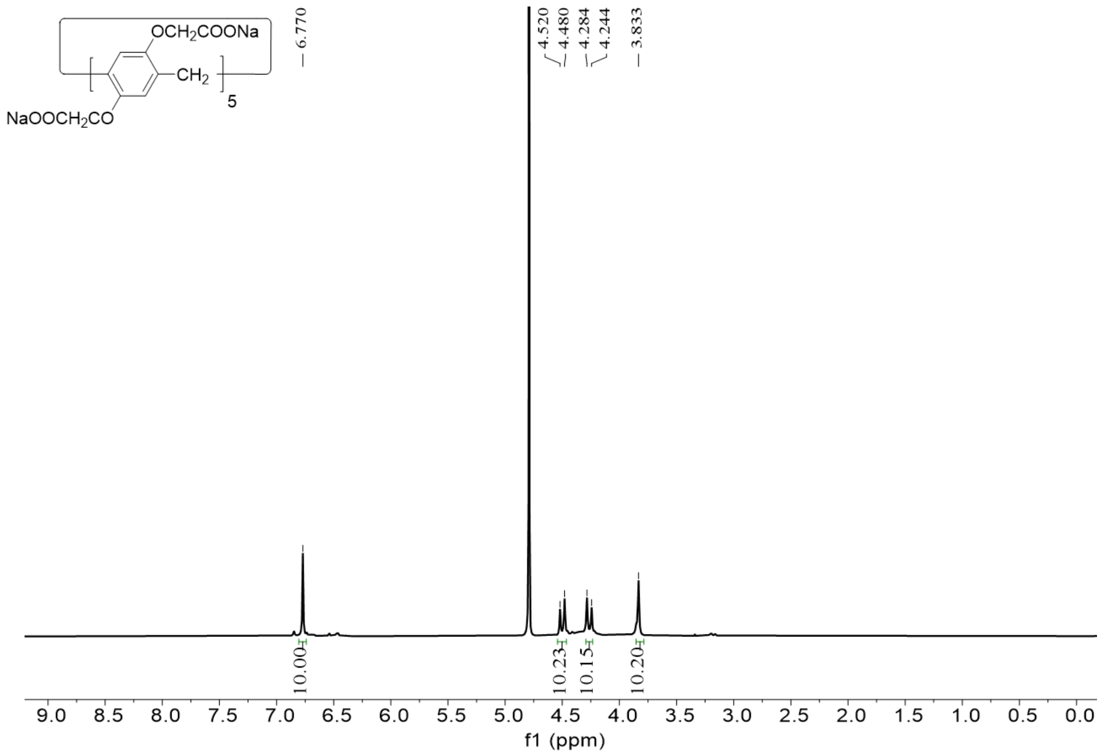

Supplementary Figure 5. ^{1}H NMR spectra of **WP5-Na** in D_2O at 298 K.

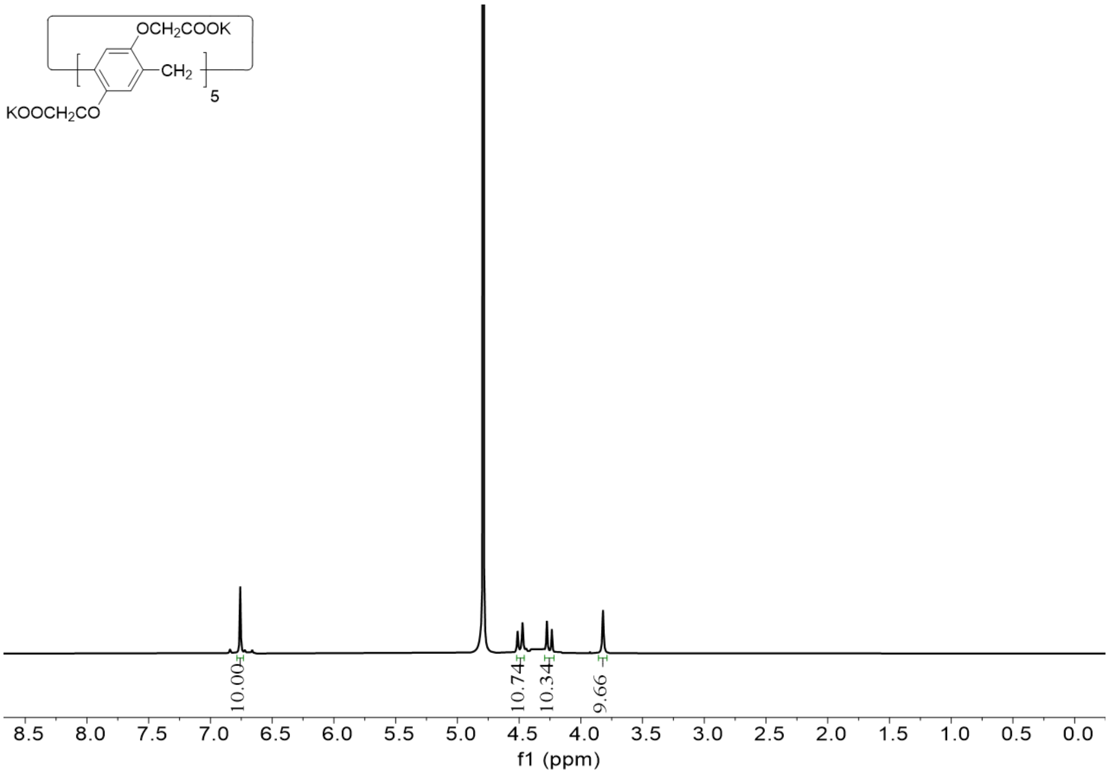

Supplementary Figure 6. ^{1}H NMR spectra of **WP5-K** in D_2O at 298 K.

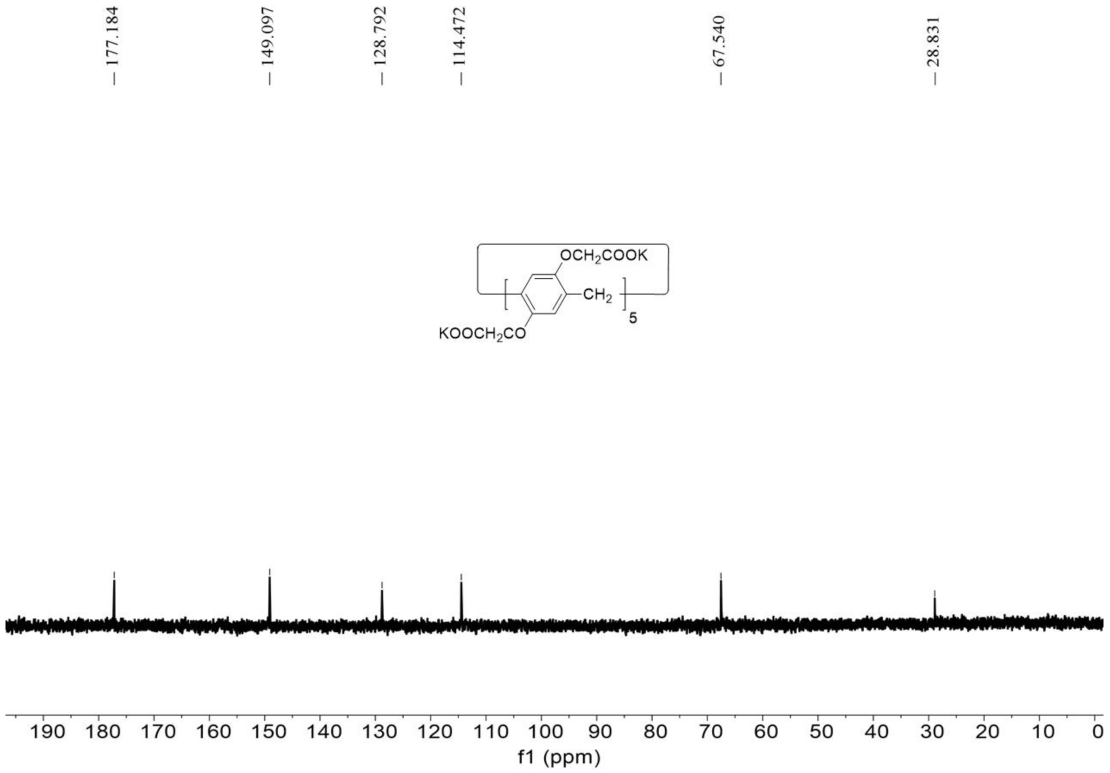

Supplementary Figure 7. ^{13}C NMR spectra of **WP5-K** in D_2O at 298 K.

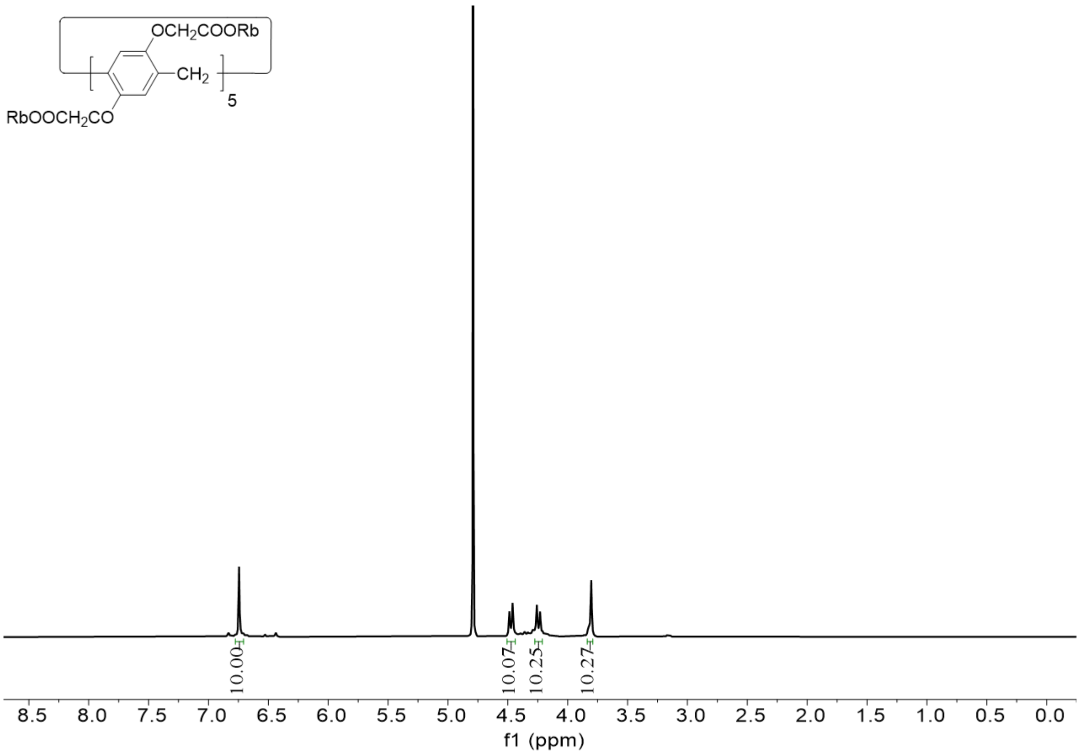

Supplementary Figure 8. ^{1}H NMR spectra of **WP5-Rb** in D_2O at 298 K.

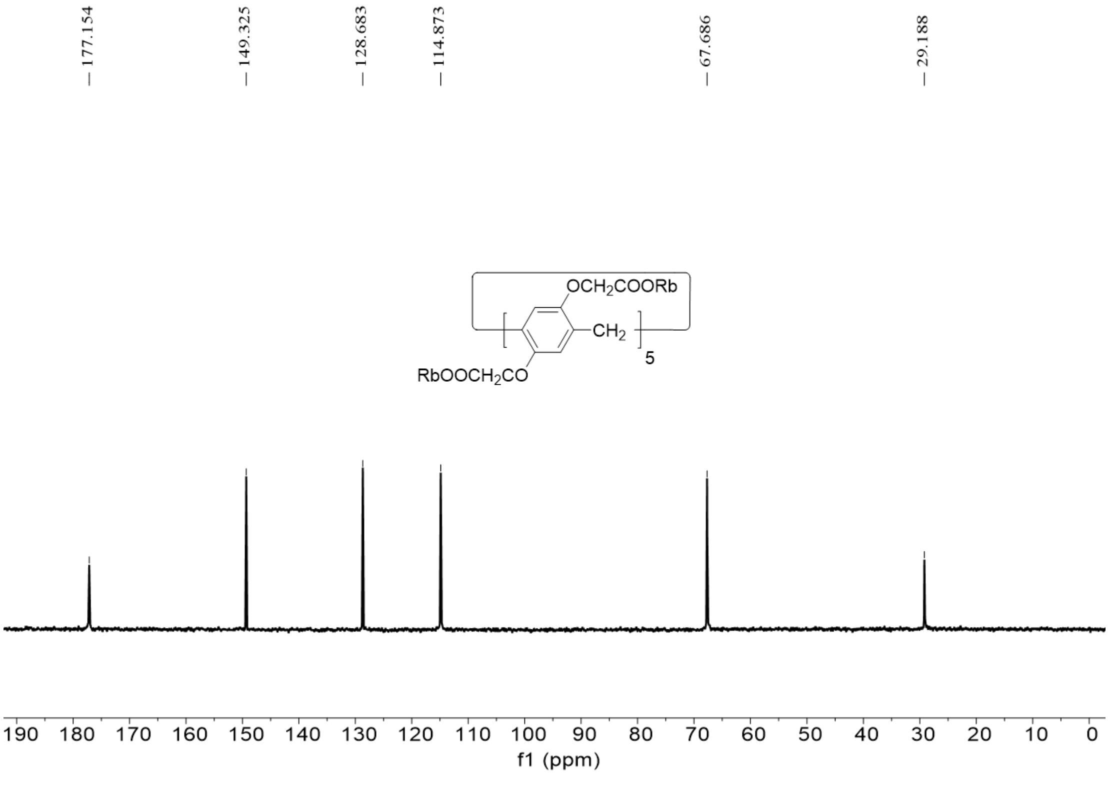

Supplementary Figure 9. ^{13}C NMR spectra of **WP5-Rb** in D_2O at 298 K.

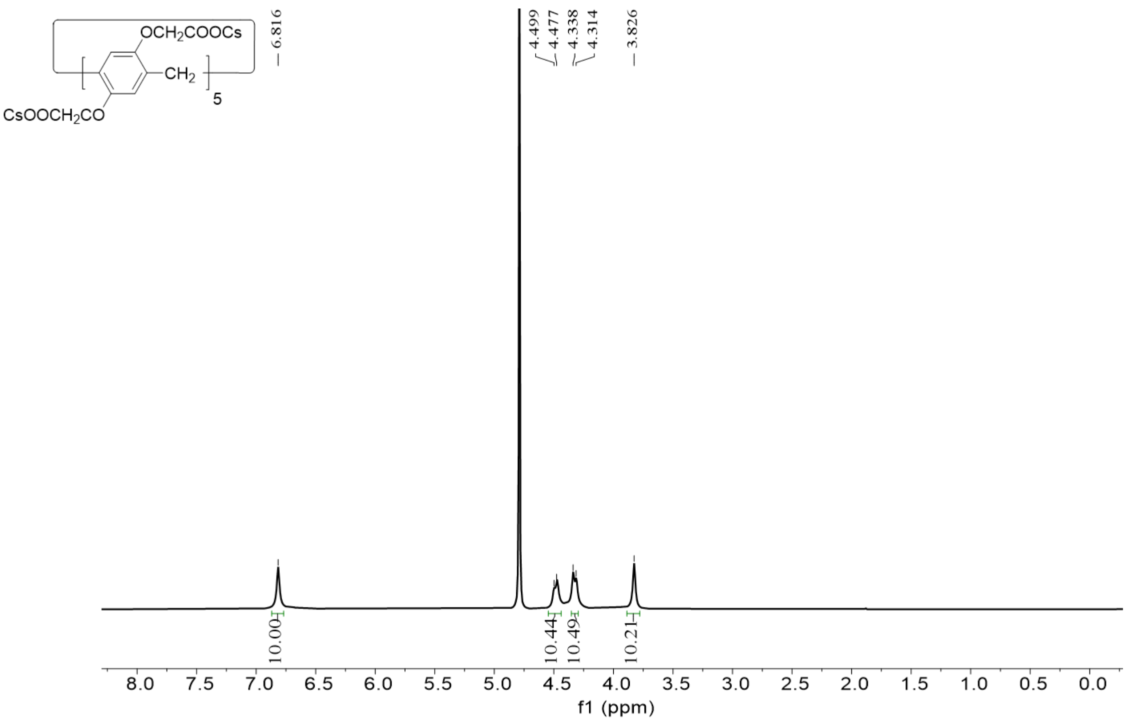

Supplementary Figure 10. ^{1}H NMR spectra of **WP5-Cs** in D_2O at 298 K.

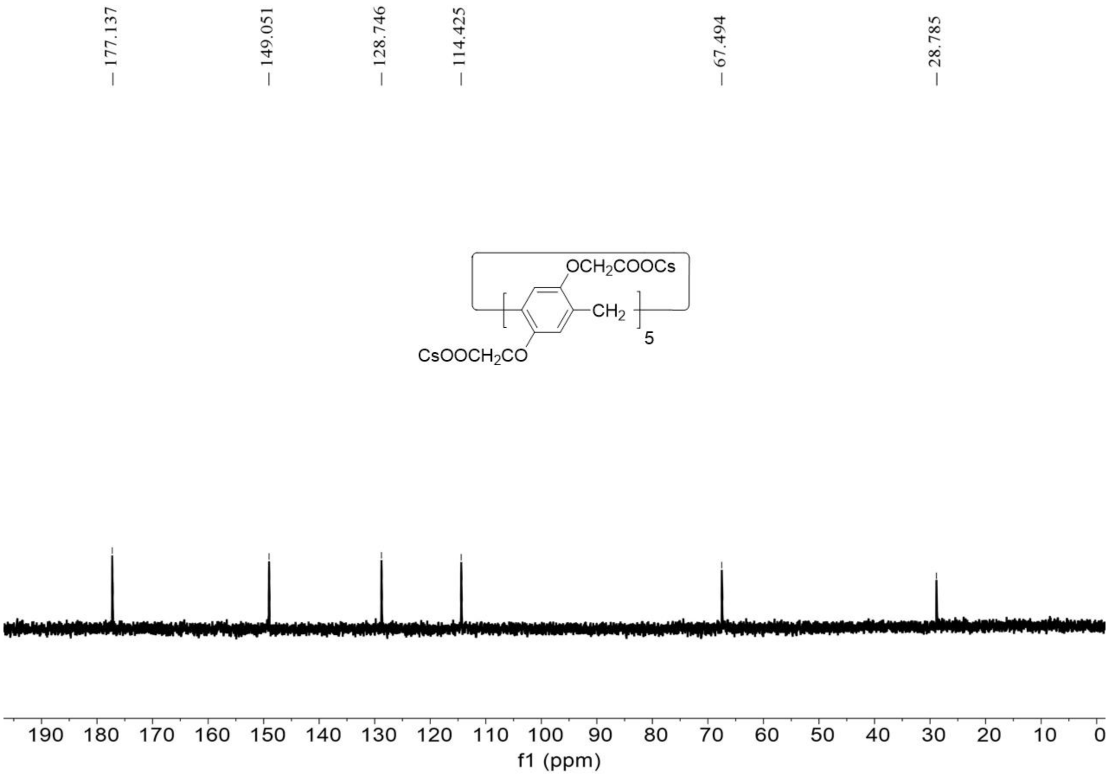

Supplementary Figure 11. ^{13}C NMR spectra of **WP5-Cs** in D_2O at 298 K.

(2). VT NMR spectrum and Eyring plots of WP5-M

Since the chemical shift of deuterium oxide in NMR spectrum could shift with the increase of temperature, the chemical shifts (δ) for VT ^{1}H NMR spectra, given in ppm, are referenced to the proton signal of phenolic rings in **WP5-M**.

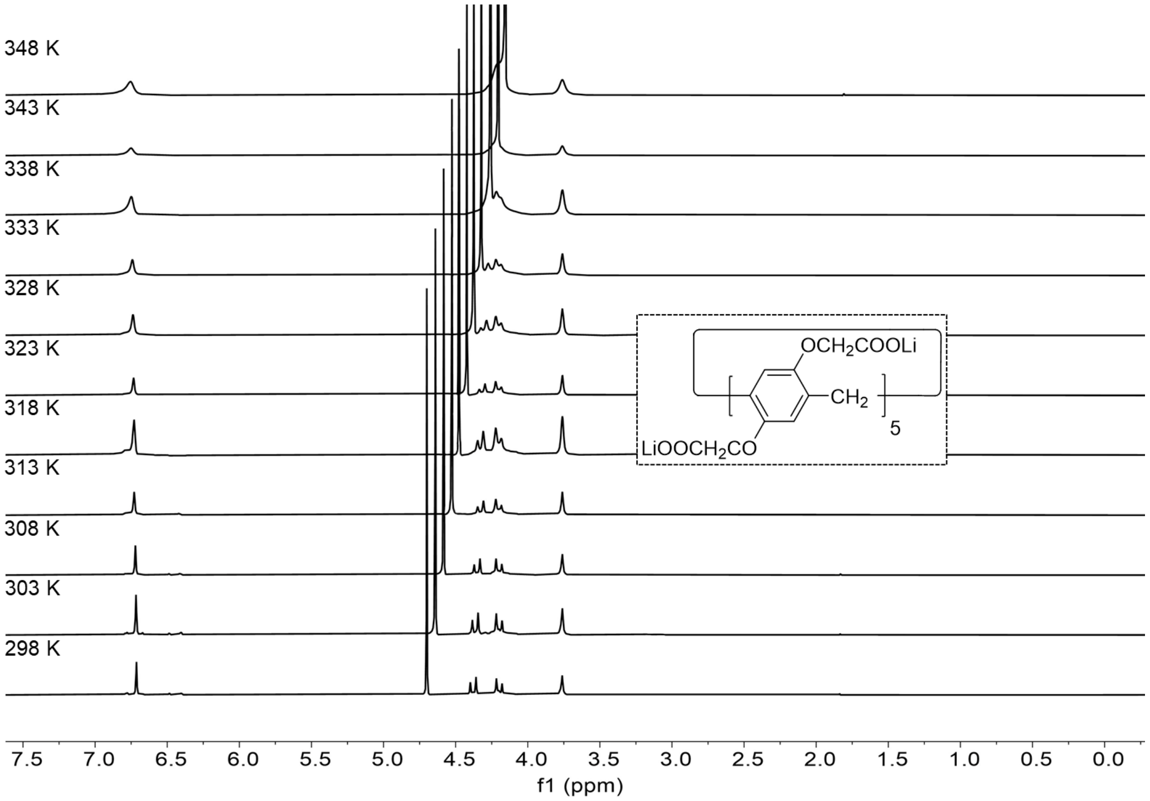

Supplementary Figure 12. VT NMR spectra of **WP5-Li** in D_2O between 298 K and 348 K.

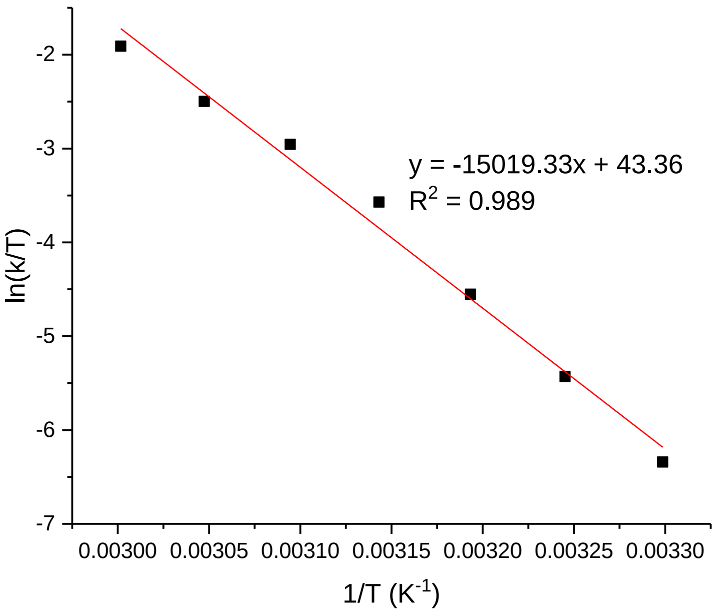

Supplementary Figure 13. Eyring plot of the rates of exchange in D_2O obtained from line width analysis of methene protons on **WP5-Li**. The barrier ($\Delta G^{\ddagger}$ = 18.23 kcal·mol^{-1}) was calculated from the slope ($\Delta H^{\ddagger}$ = 29.83 kcal·mol^{-1}) and y-intercept ($\Delta S^{\ddagger}$ = 38.92 cal·mol^{-1}·K^{-1}).

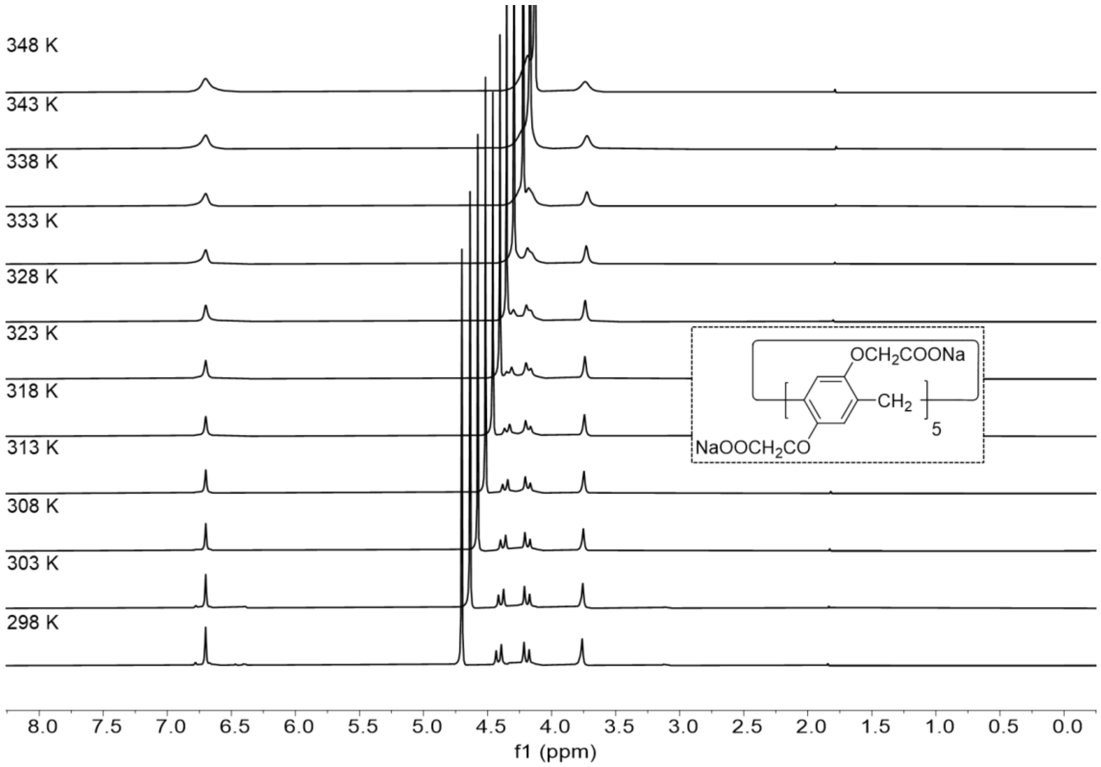

Supplementary Figure 14. VT NMR spectra of **WP5-Na** in D_2O between 298 K and 348 K.

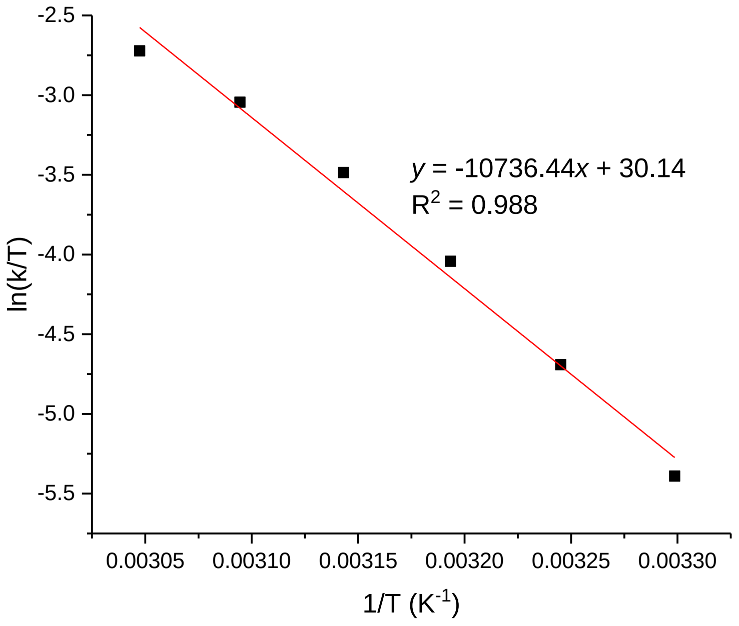

Supplementary Figure 15. Eyring plot of the rates of exchange in D_2O obtained from line width analysis of methene protons on **WP5-Na**. The barrier ($\Delta G^{\ddagger}$ = 17.55 kcal·mol^{-1}) was calculated from the slope ($\Delta H^{\ddagger}$ = 21.32 kcal·mol^{-1}) and y-intercept ($\Delta S^{\ddagger}$ = 12.67 cal·mol^{-1}·K^{-1}).

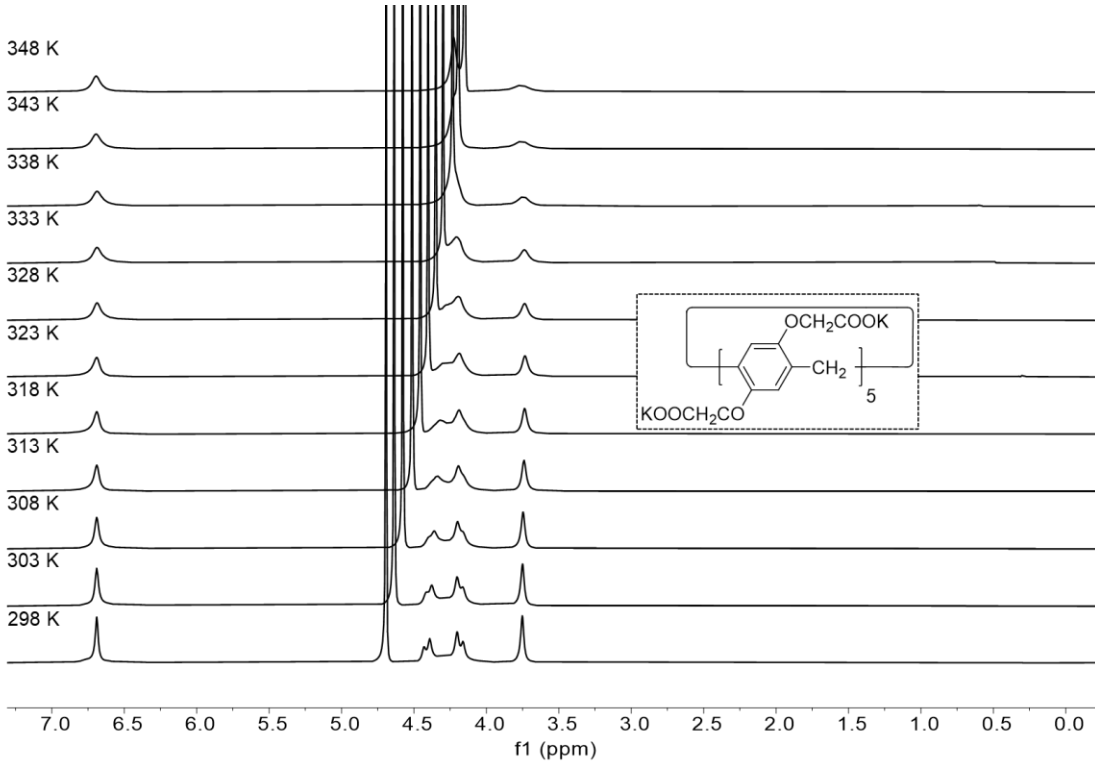

Supplementary Figure 16. VT NMR spectra of **WP5-K** in D_2O between 298 K and 348 K.

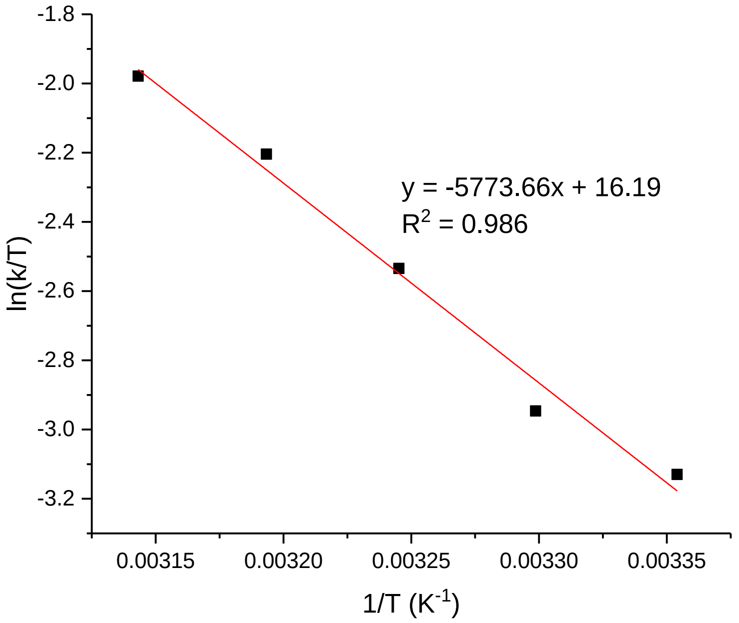

Supplementary Figure 17. Eyring plot of the rates of exchange in D_2O obtained from line width analysis of methene protons on **WP5-K**. The barrier ($\Delta G^‡$ = 15.95 kcal·mol^{-1}) was calculated from the slope ($\Delta H^‡$ = 11.47 kcal·mol^{-1}) and y-intercept ($\Delta S^‡$ = -15.04 cal·mol^{-1}·K^{-1}).

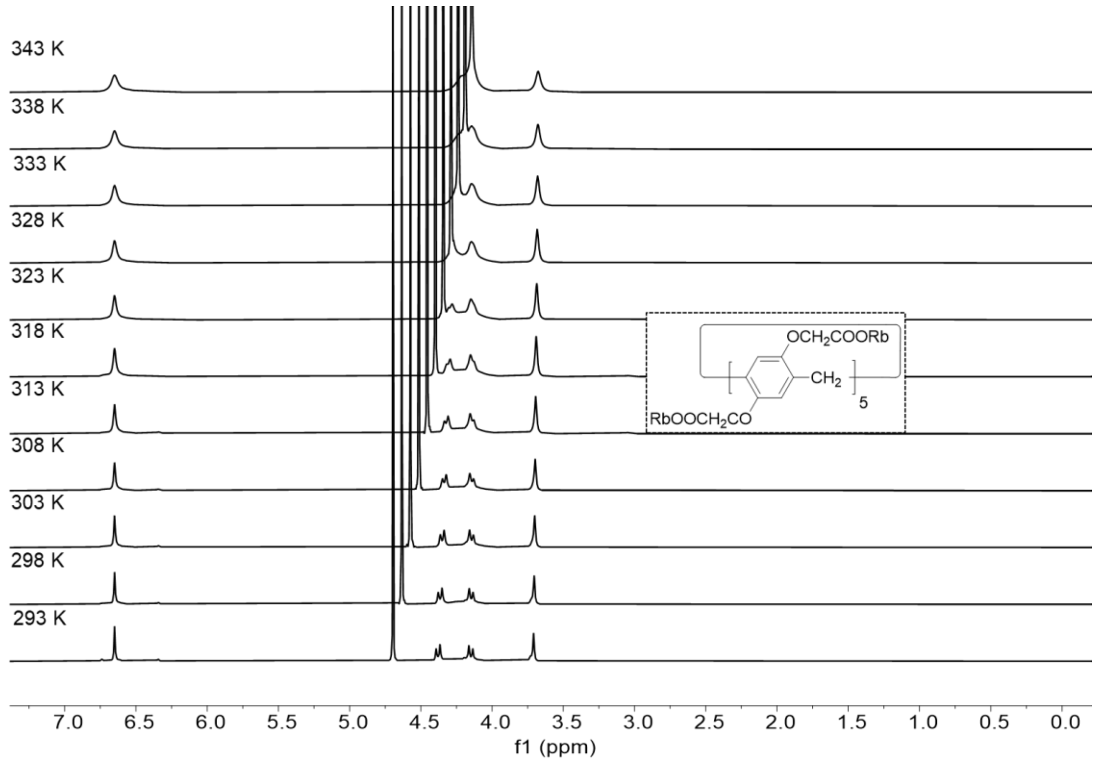

Supplementary Figure 18. VT NMR spectra of **WP5-Rb** in D_2O between 293 K and 343 K.

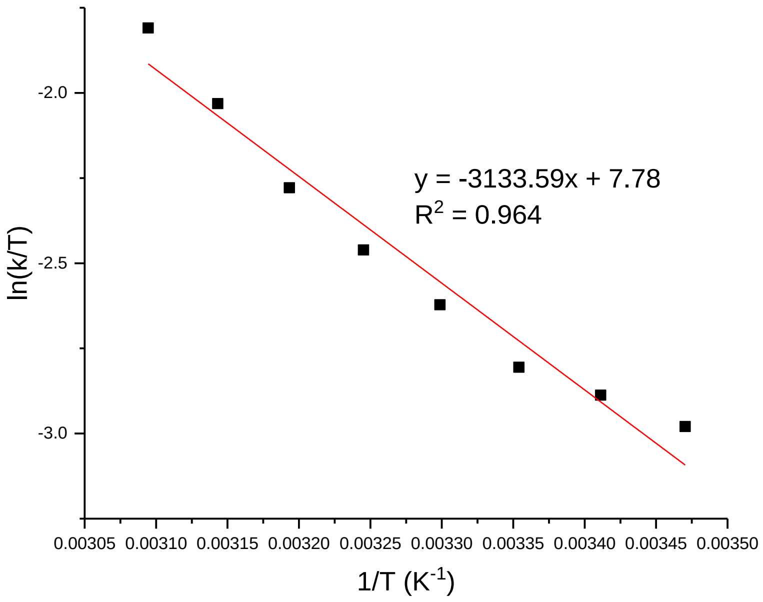

Supplementary Figure 19. Eyring plot of the rates of exchange in D_2O obtained from line width analysis of methene protons on **WP5-Rb**. The barrier ($\Delta G^‡$ = 15.68 kcal·mol^{-1}) was calculated from the slope ($\Delta H^‡$ = 6.22 kcal·mol^{-1}) and y-intercept ($\Delta S^‡$ = -31.74 cal·mol^{-1}·K^{-1}).

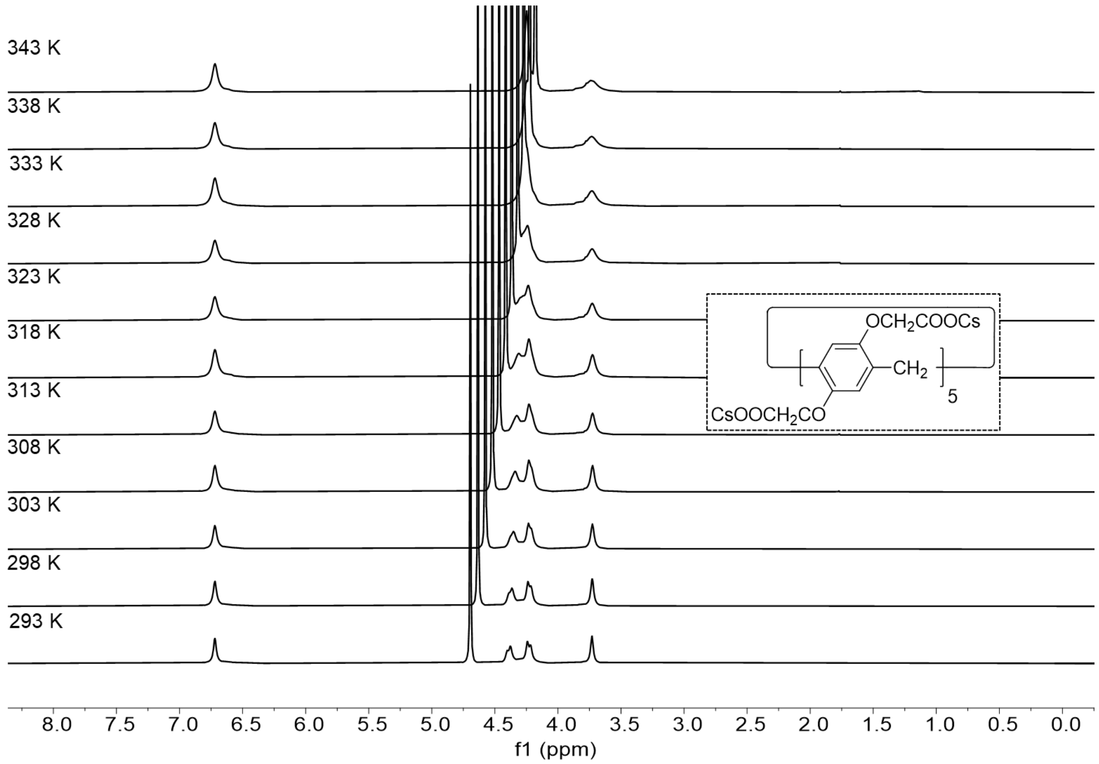

Supplementary Figure 20. VT NMR spectra of **WP5-Cs** in D_2O between 293 K and 343 K.

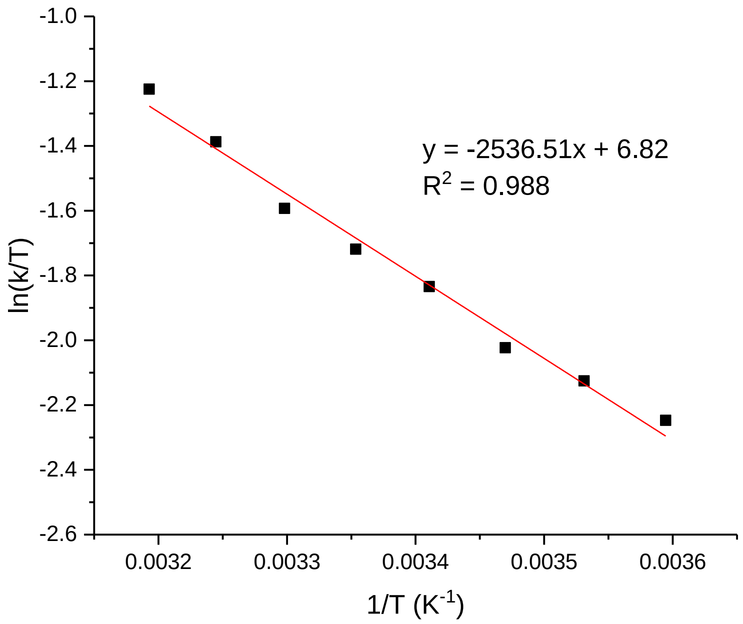

Supplementary Figure 21. Eyring plot of the rates of exchange in D_2O obtained from line width analysis of methene protons on **WP5-Cs**. The barrier ($\Delta G^{\ddagger}$ = 15.06 kcal·mol^{-1}) was calculated from the slope ($\Delta H^{\ddagger}$ = 5.04 kcal·mol^{-1}) and y-intercept ($\Delta S^{\ddagger}$ = -33.65 cal·mol^{-1}·K^{-1}).

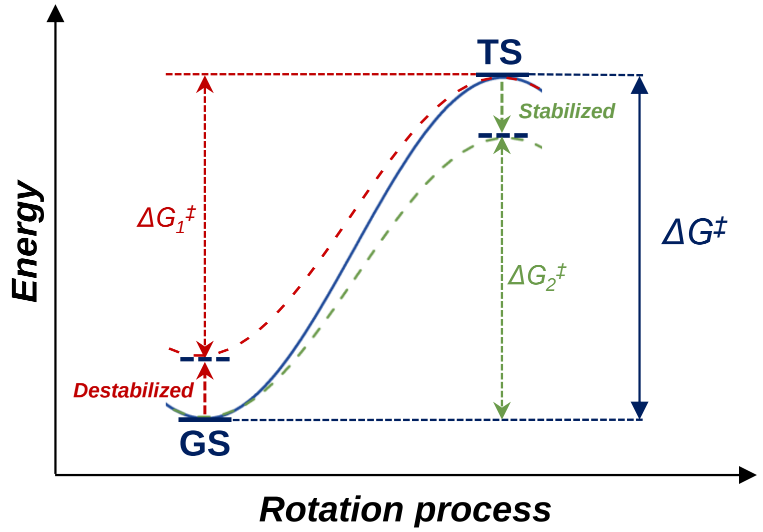

Supplementary Figure 22. Relative energy between ground state (GS) and transition state (TS) of **WP5-M**. The impact of counter cations on GS or TS could change rotational barriers.

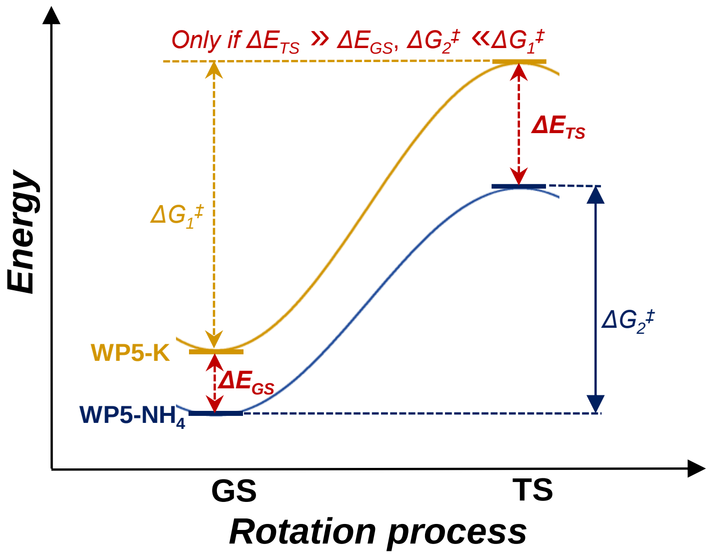

Supplementary Figure 23. Relative energy between ground state (GS) and transition state (TS) of **WP5-K** and **WP5-NH4**. Only if ΔE_{TS} was much bigger than ΔE_{GS}, rotational barriers of **WP5-NH4** would much lower than that of **WP5-K** (*i.e.*, complied with experimental results), which indicated the effect of cations on transition states was much greater than that on ground states.

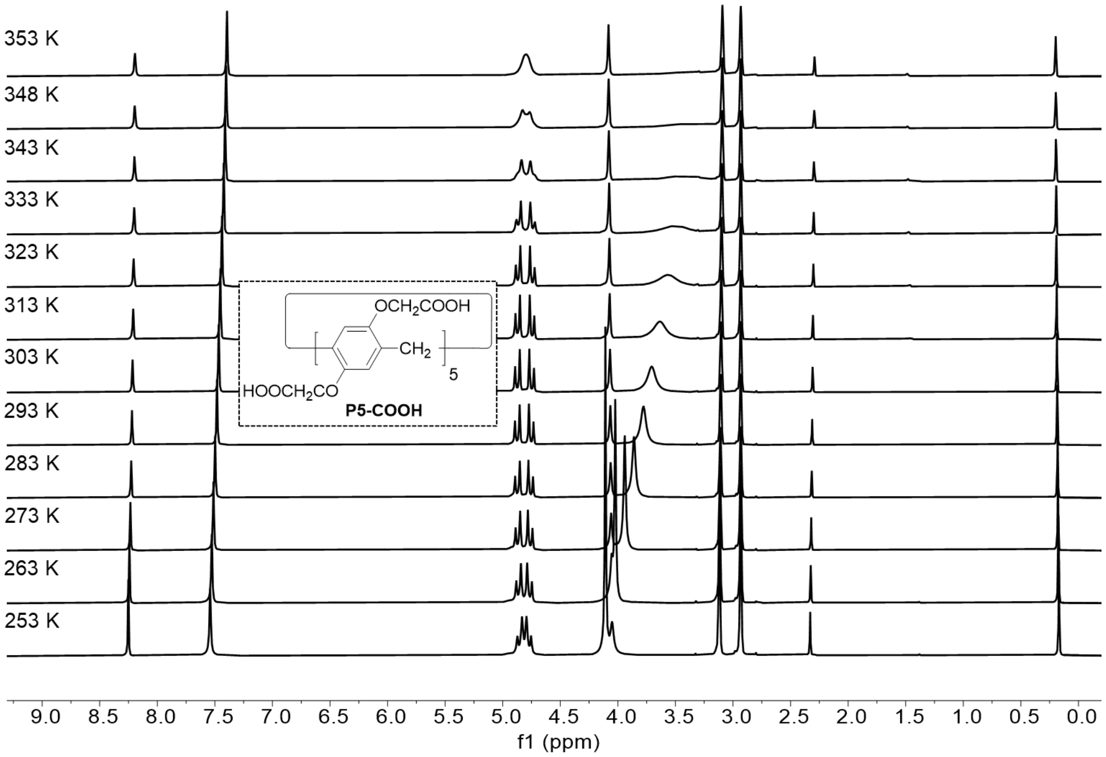

Supplementary Figure 24. VT NMR spectra of **P5-COOH** in DMF-d_6/H_2O (v/v = 3/1) between 253 K and 353 K. The coalesce temperature was 353 K.

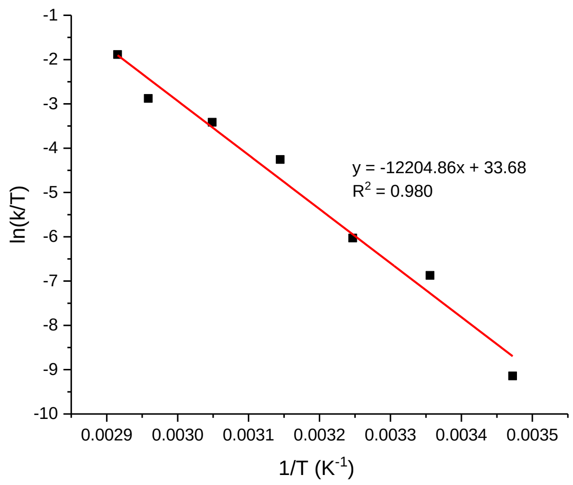

Supplementary Figure 25. Eyring plot of the rates of exchange in DMF-d_6/H_2O (v/v = 3/1) obtained from line width analysis of methene protons on **P5-COOH**. The barrier ($\Delta G^{\ddagger}$ = 18.37 kcal·mol^{-1}) was calculated from the slope ($\Delta H^{\ddagger}$ = 24.24 kcal·mol^{-1}) and y-intercept ($\Delta S^{\ddagger}$ = 19.70 cal·mol^{-1}·K^{-1}).

(3). UV titration plots

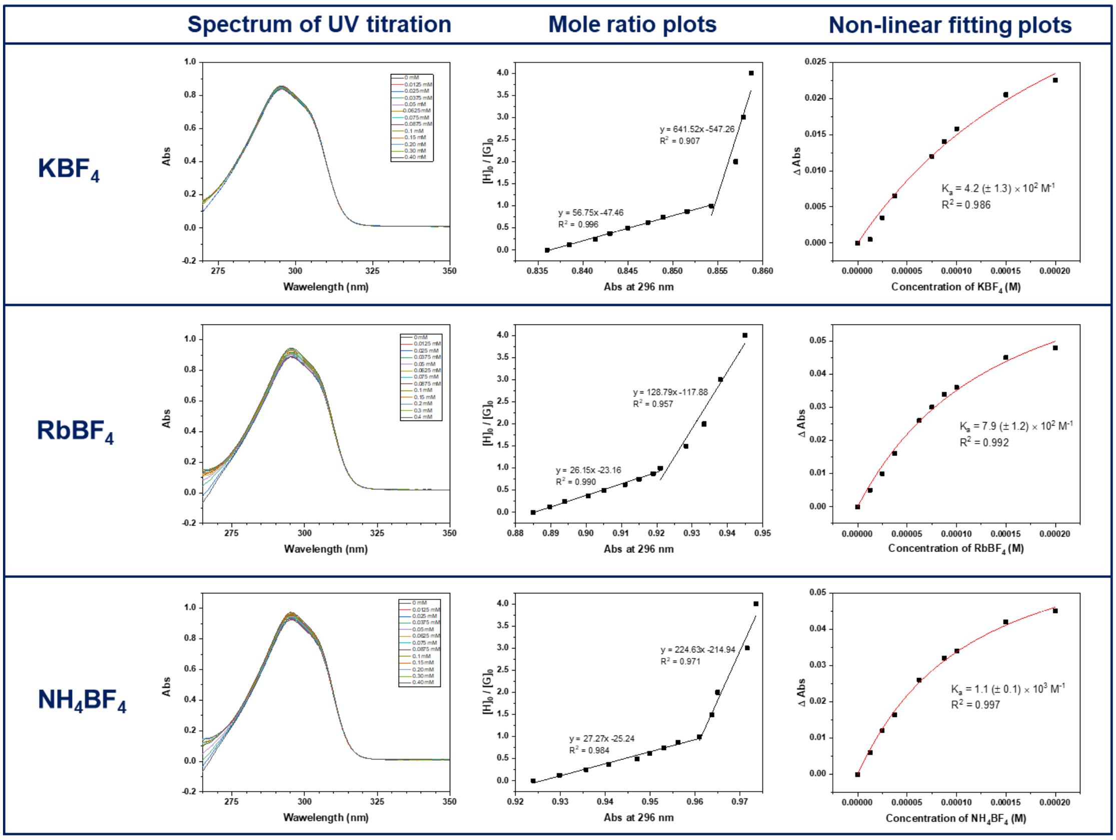

Supplementary Figure 26. Titration curve (left), mole ratio plot (mid) and non-linear fitting curve (right) of host **P5-COOEt** and guest K^+, Rb^+, and NH_4^+ in DMF/H_2O solution (v/v = 4/1), The mole ratio plots for the complexation between **P5-COOEt** and cations, indicating a 1:1 stoichiometry.

(4). Theoretical computation

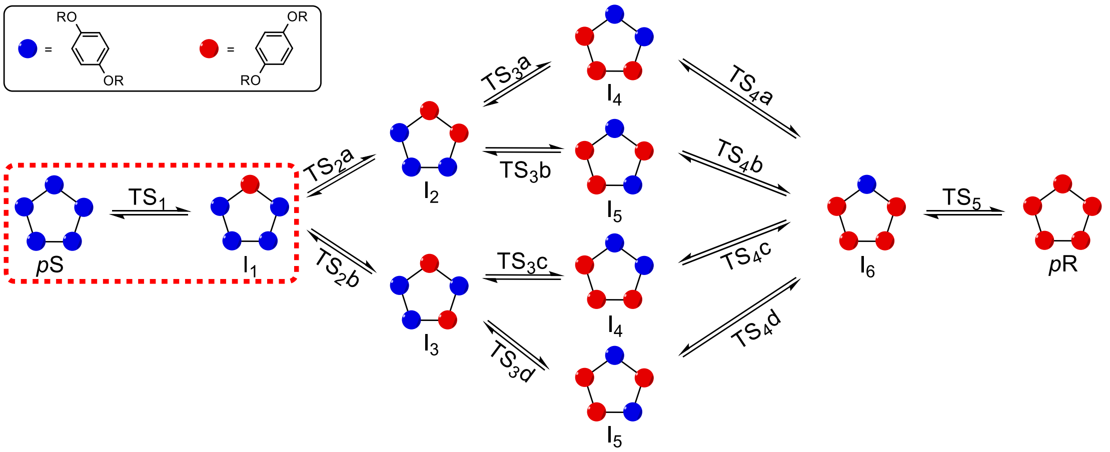

Supplementary Figure 27. Pathways of transformation between *p*S and *p*R conformers of **WP5-M**.

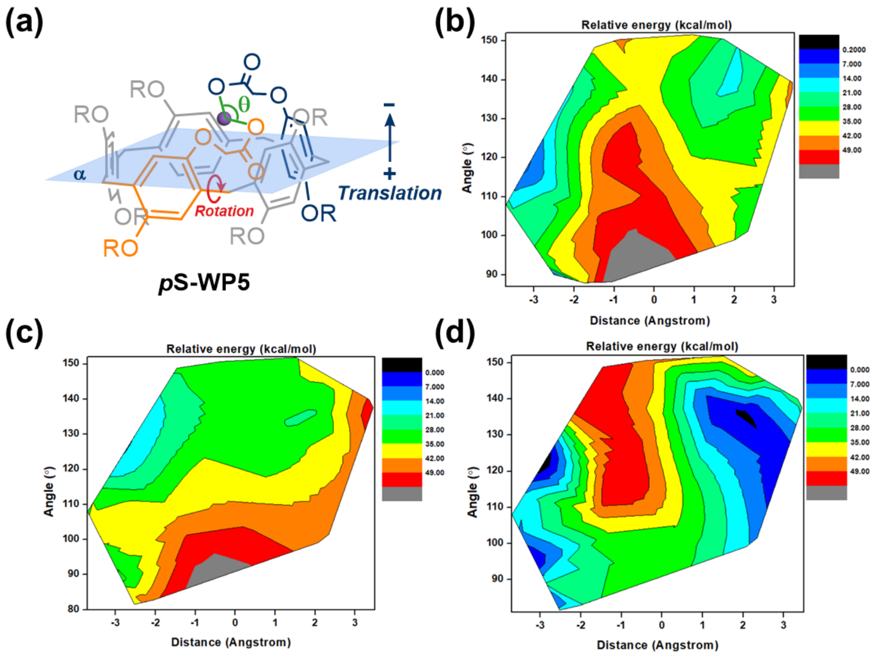

Supplementary Figure 28. (a) Illustration of configuration for **WP5** when ion is located in different positions of cavity. The potential energy surface of **WP5** with Li (b), Na (c) and K (d) using semi-empirical PM6 method with dispersion correction (PM6-D3).

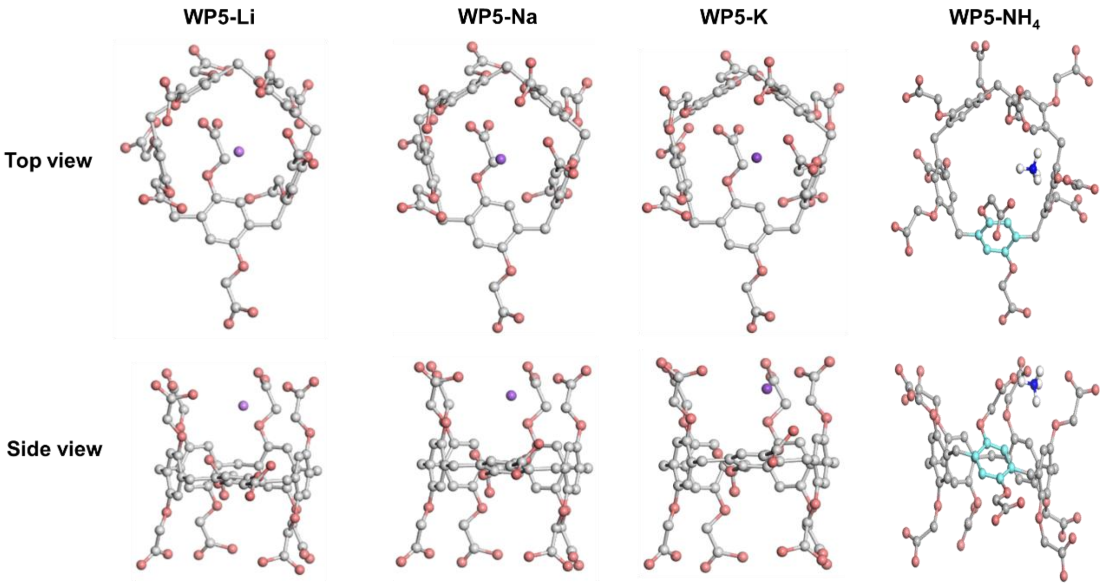

Supplementary Figure 29. The sampled highest-energy configurations from the scanned structures at the PM6-D3 level for **WP5-Li**, **WP5-Na**, **WP5-K** and **WP5-NH4**.

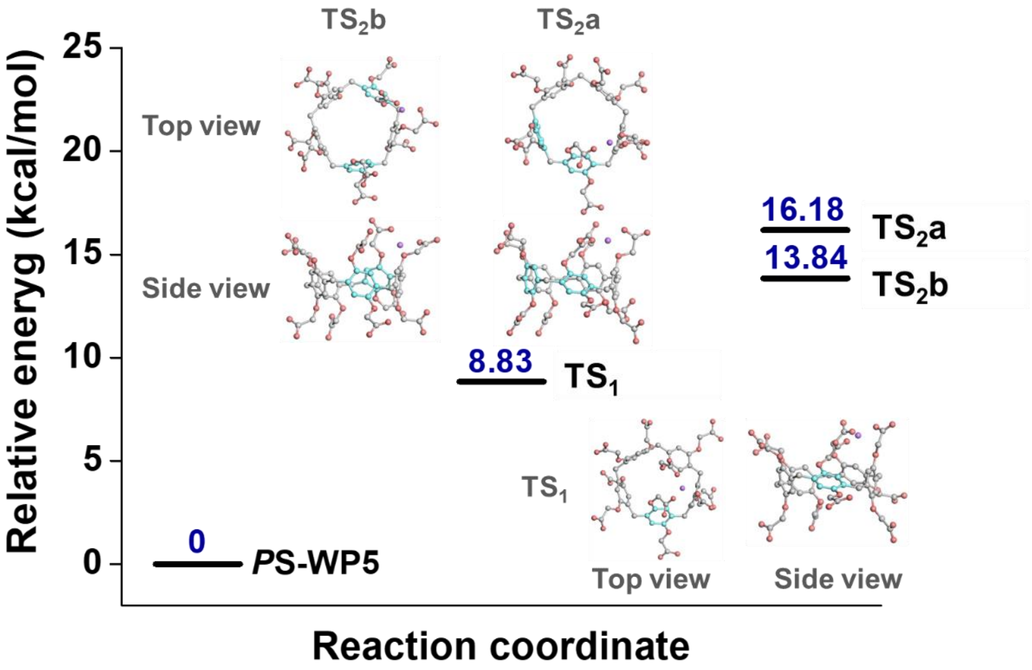

Supplementary Figure 30. Relative energy diagram and optimized geometric structures of two intermediates in TS_1 and TS_2 for **WP5-Li** using B3LYP-D3 functional.

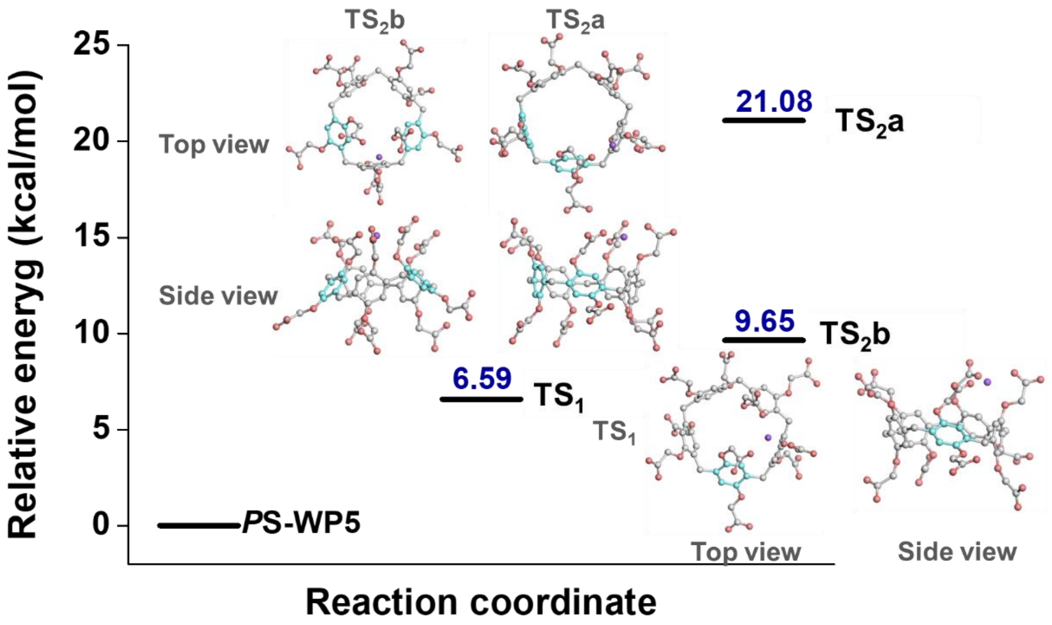

Supplementary Figure 31. Relative energy diagram and optimized geometric structures of two intermediates in TS_1 and TS_2 for **WP5-Na** using B3LYP-D3 functional.

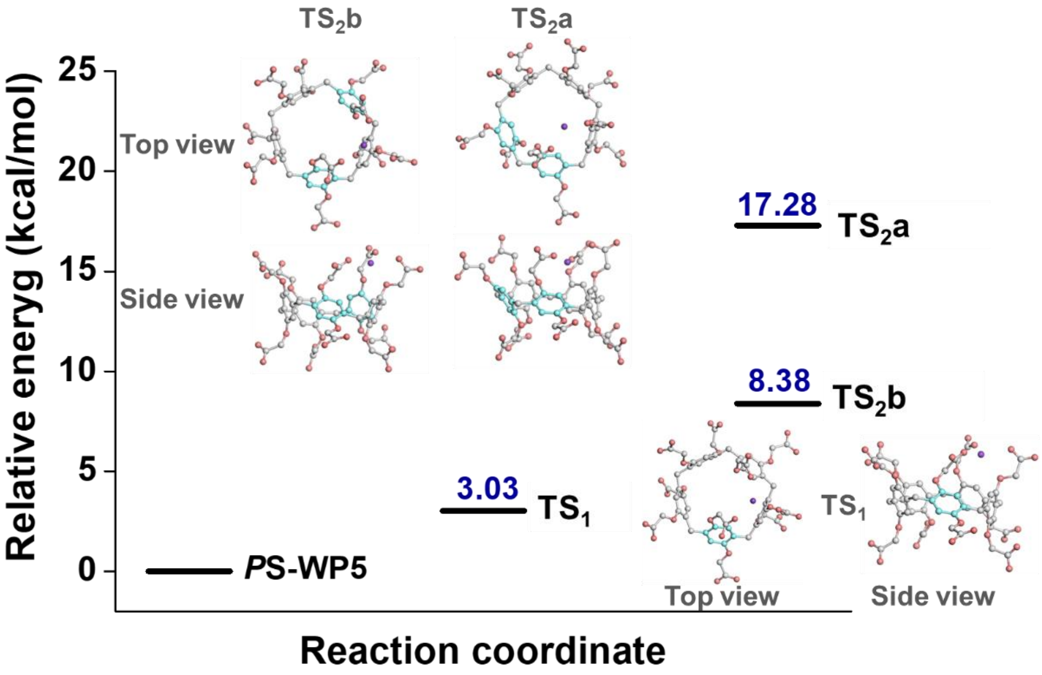

Supplementary Figure 32. Relative energy diagram and optimized geometric structures of two intermediates in TS_1 and TS_2 for **WP5-K** using B3LYP-D3 functional.

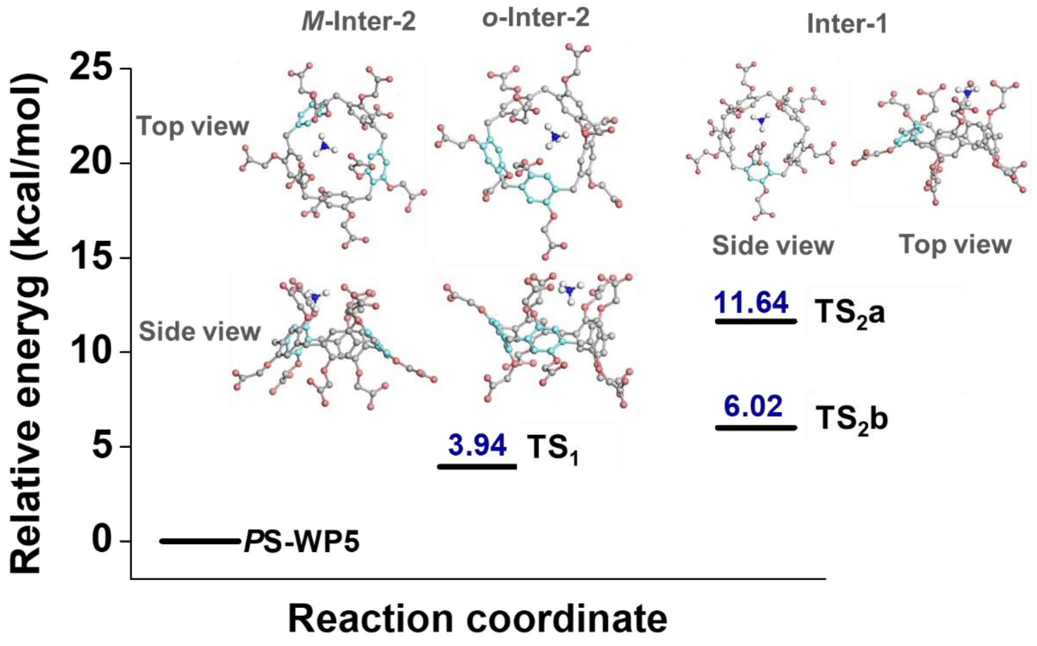

Supplementary Figure 33. Relative energy diagram and optimized geometric structures of two intermediates in TS_1 and TS_2 for **WP5-NH$_4$** using B3LYP-D3 functional.

(5). Solvent effect

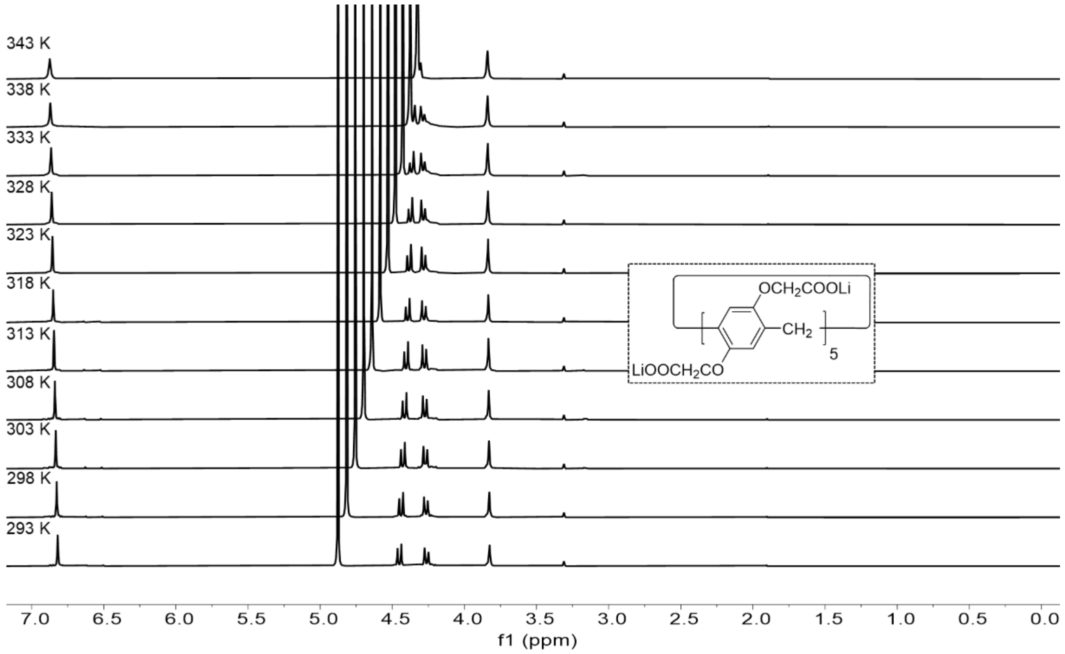

Supplementary Figure 34. VT NMR spectra of **WP5-Li** in mixed solvent (v/v of D_2O/methanol-d_4 = 2:1) between 293 K and 343 K.

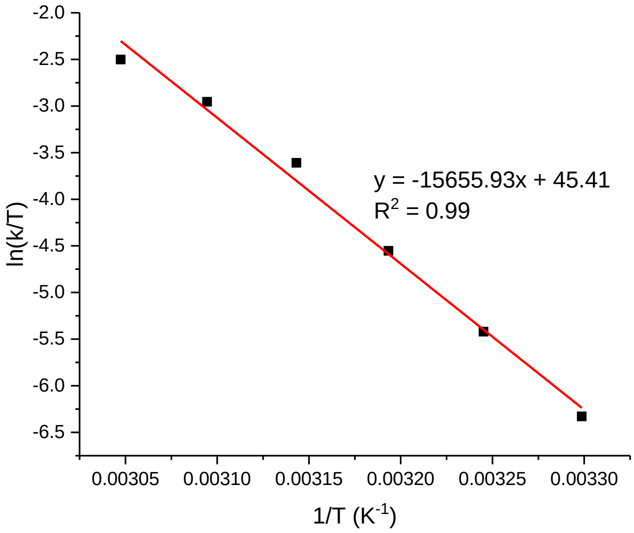

Supplementary Figure 35. Eyring plot of the rates of exchange in mixed solvent obtained from line width analysis of methene protons on **WP5-Li**. The barrier ($\Delta G^{\ddagger}$ = 18.28 kcal·mol^{-1}) was calculated from the slope ($\Delta H^{\ddagger}$ = 31.09 kcal·mol^{-1}) and y-intercept ($\Delta S^{\ddagger}$ = 42.99 cal·mol^{-1}·K^{-1}).

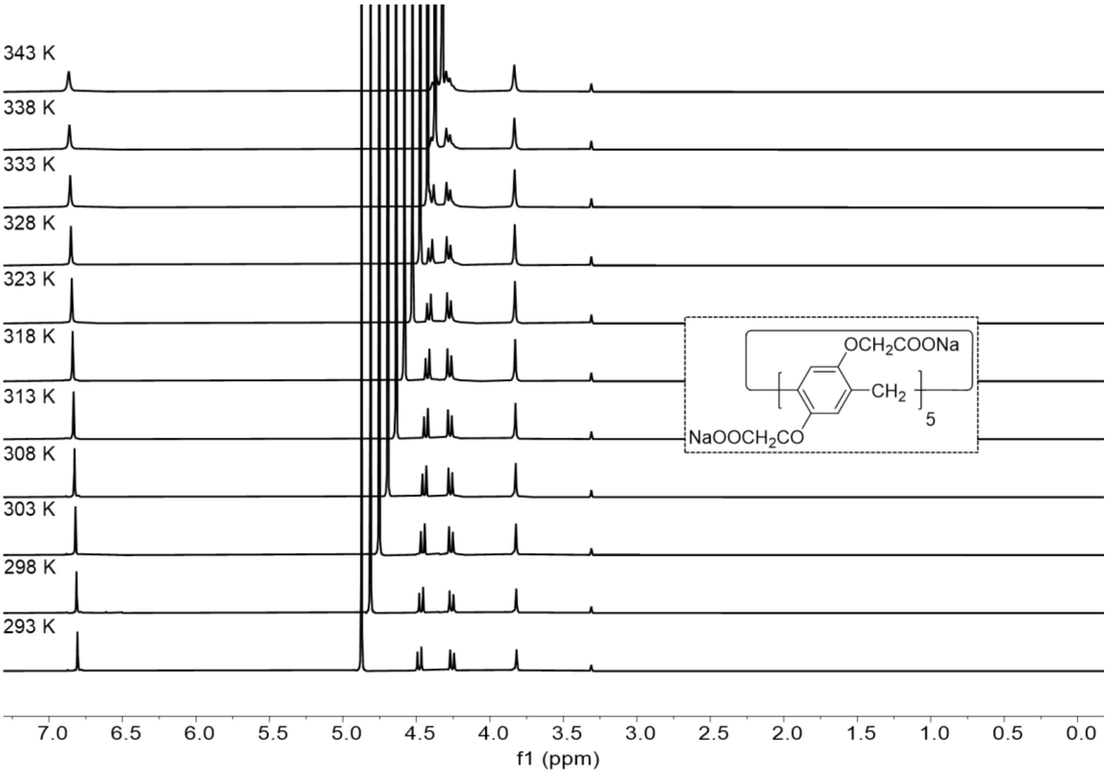

Supplementary Figure 36. VT NMR spectra of **WP5-Na** in mixed solvent (v/v of D_2O/methanol-d_4 = 2:1) between 293 K and 343 K.

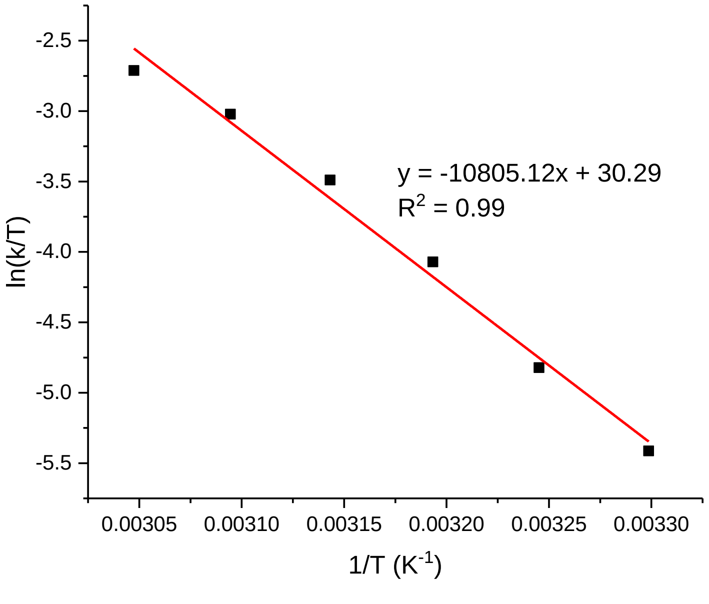

Supplementary Figure 37. Eyring plot of the rates of exchange in mixed solvent obtained from line width analysis of methene protons on **WP5-Na**. The barrier ($\Delta G^{‡}$ = 17.59 kcal·mol^{-1}) was calculated from the slope ($\Delta H^{‡}$ = 21.46 kcal·mol^{-1}) and y-intercept ($\Delta S^{‡}$ = 12.97 cal·mol^{-1}·K^{-1}).

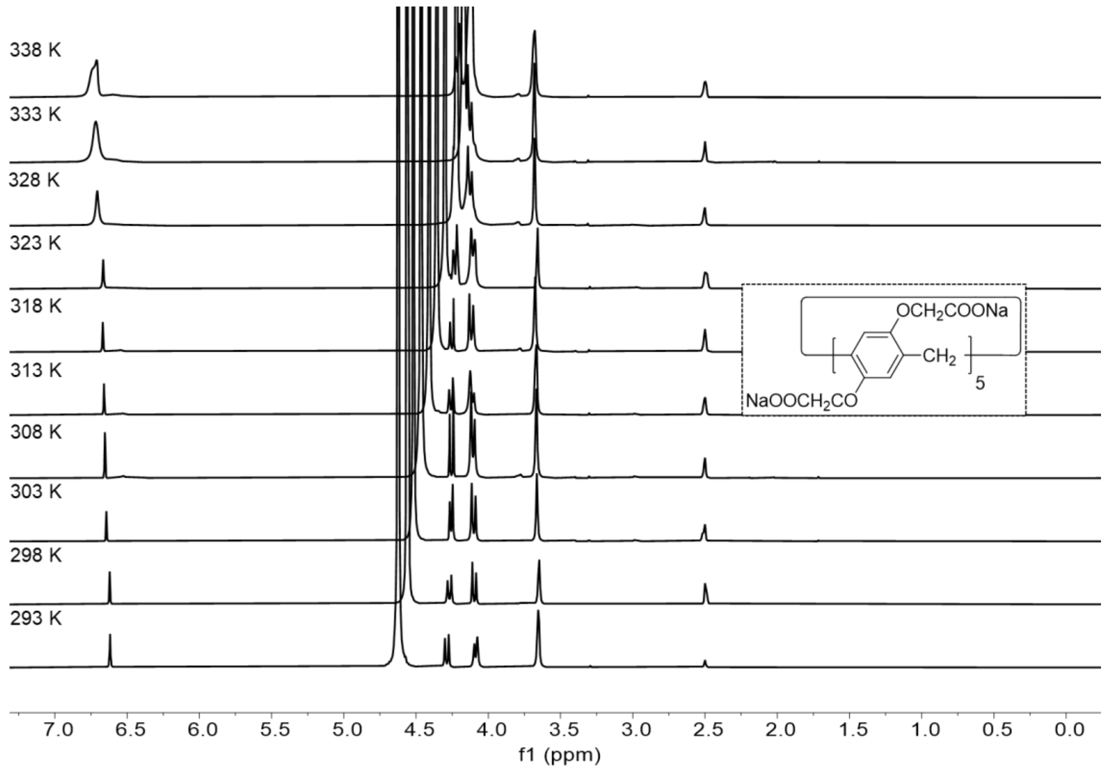

Supplementary Figure 38. VT NMR spectra of **WP5-Na** in mixed solvent (v/v of D_2O/DMSO-d_6 = 2:1) between 293 K and 338 K.

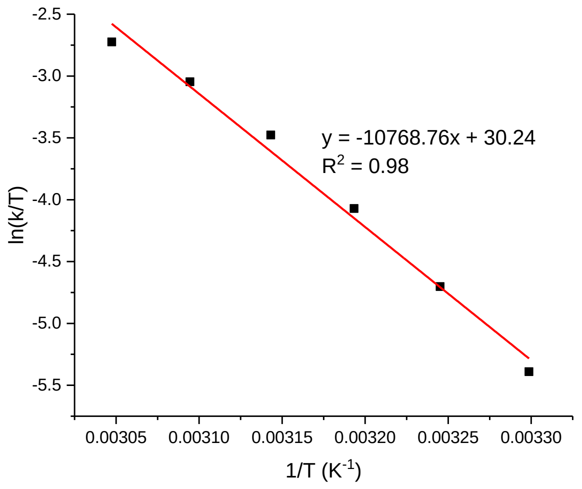

Supplementary Figure 39. Eyring plot of the rates of exchange in mixed solvent obtained from line width analysis of methene protons on **WP5-Na**. The barrier ($\Delta G^{\ddagger}$ = 17.55 kcal·mol^{-1}) was calculated from the slope ($\Delta H^{\ddagger}$ = 21.39 kcal·mol^{-1}) and y-intercept ($\Delta S^{\ddagger}$ = 12.87 cal·mol^{-1}·K^{-1}).

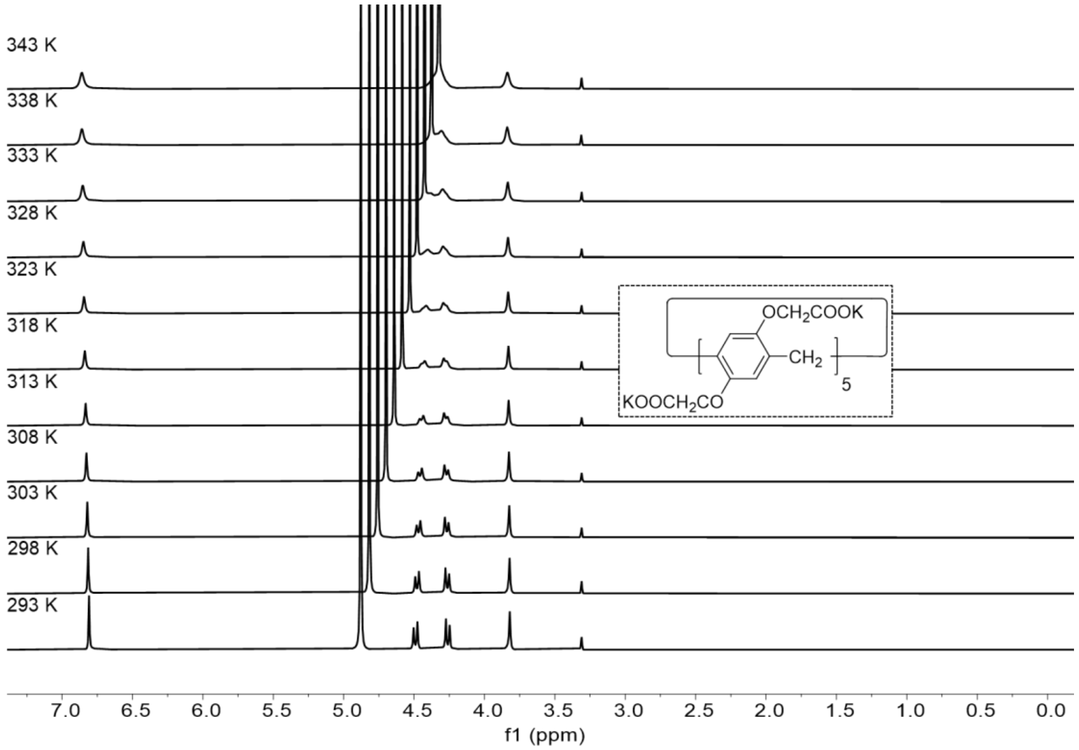

Supplementary Figure 40. VT NMR spectra of **WP5-K** in mixed solvent (v/v of D_2O/methanol-d_4 = 2:1) between 293 K and 343 K.

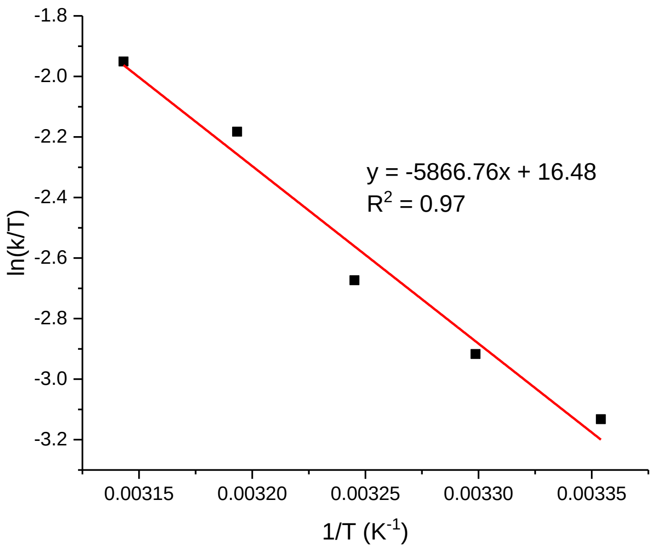

Supplementary Figure 41. Eyring plot of the rates of exchange in mixed solvent obtained from line width analysis of methene protons on **WP5-K**. The barrier ($\Delta G^‡$ = 15.96 kcal·mol^{-1}) was calculated from the slope ($\Delta H^‡$ = 11.65 kcal·mol^{-1}) and y-intercept ($\Delta S^‡$ = -14.46 cal·mol^{-1}·K^{-1}).

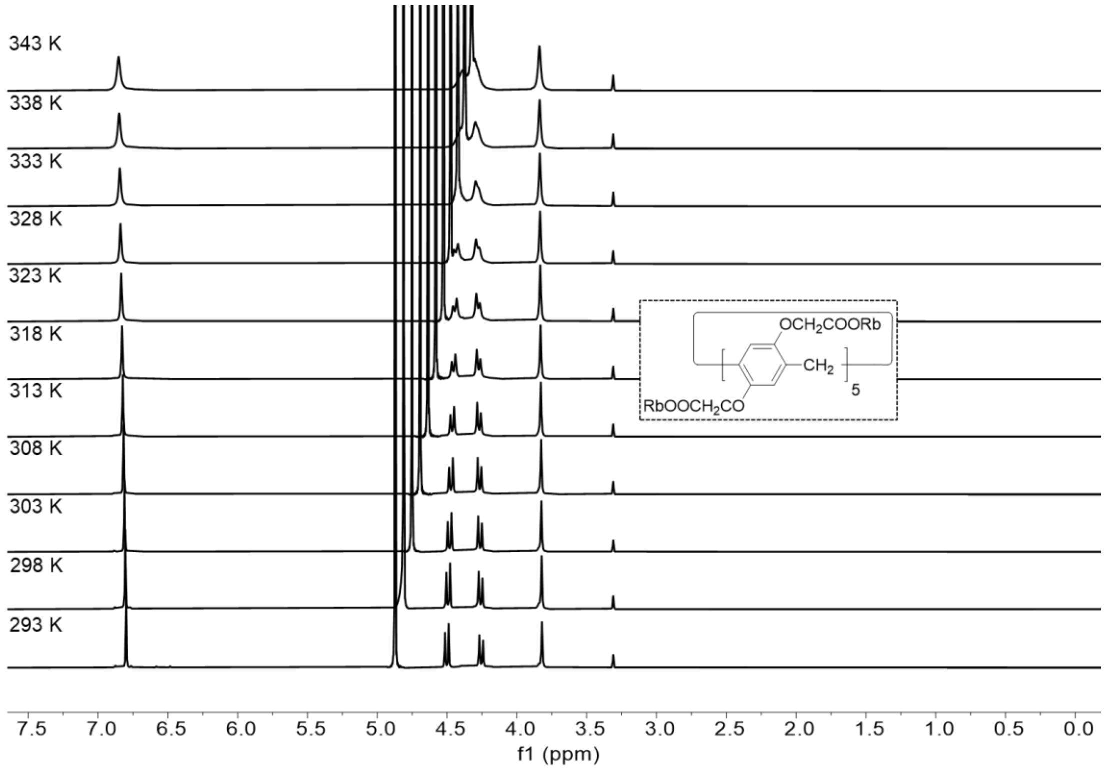

Supplementary Figure 42. VT NMR spectra of **WP5-K** in mixed solvent (v/v of D_2O/methanol-d_4 = 2:1) between 293 K and 343 K.

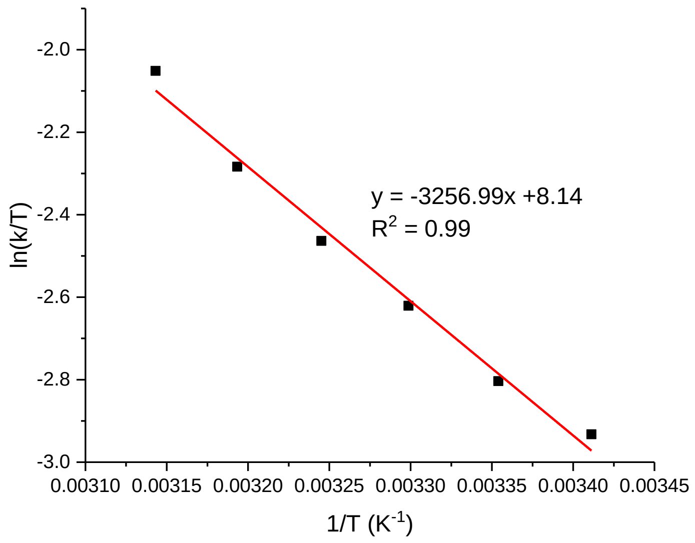

Supplementary Figure 43. Eyring plot of the rates of exchange in mixed solvent obtained from line width analysis of methene protons on **WP5-Rb**. The barrier ($\Delta G^‡$ = 15.71 kcal·mol^{-1}) was calculated from the slope ($\Delta H^‡$ = 6.47 kcal·mol^{-1}) and y-intercept ($\Delta S^‡$ = -31.02 cal·mol^{-1}·K^{-1}).

(6) Cation switch reactions and ink writing test

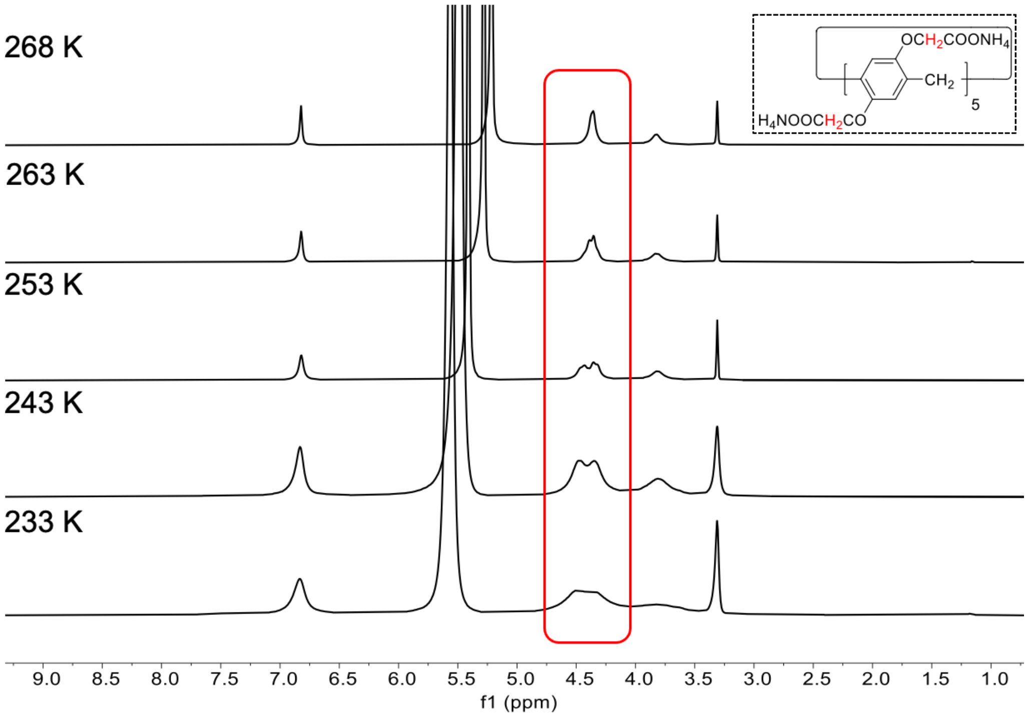

Supplementary Figure 44. VT NMR spectra of **WP5-NH4** in mixed solvent (D_2O/methanol-d_4 v/v = 3:2).

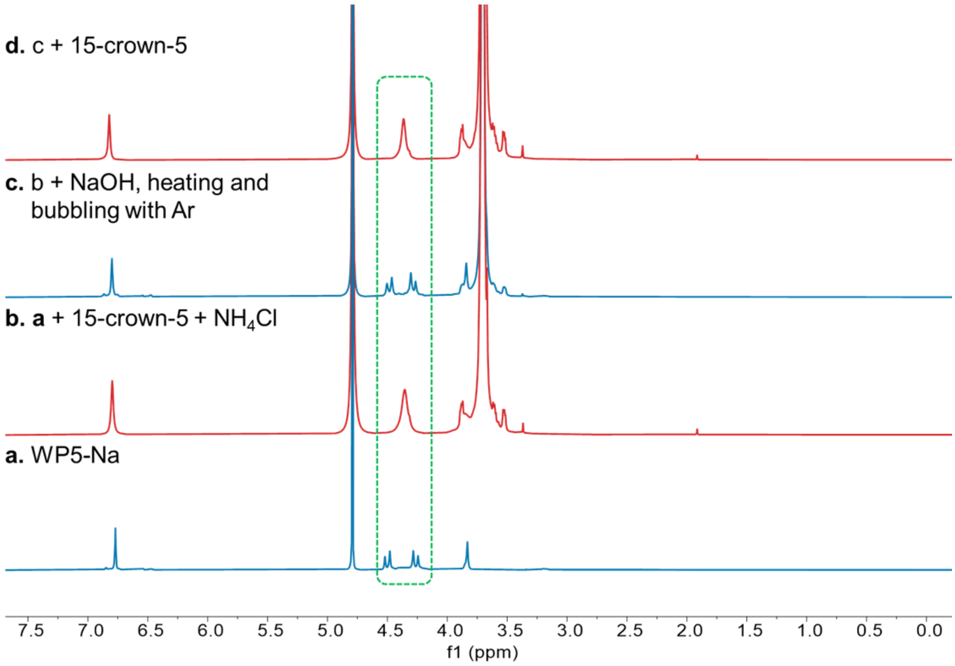

Supplementary Figure 45. Stacking NMR spectra of Na^+/NH_4^+ switching: (a). **WP5-Na** (10.0 mM); (b). a + 15-crown-5 (20.0 equiv.) + NH_4Cl (10.0 equiv.); (c). b + NaOH (10.0 equiv.), heating and bubbling with Ar; (d). c + 15-crown-5.

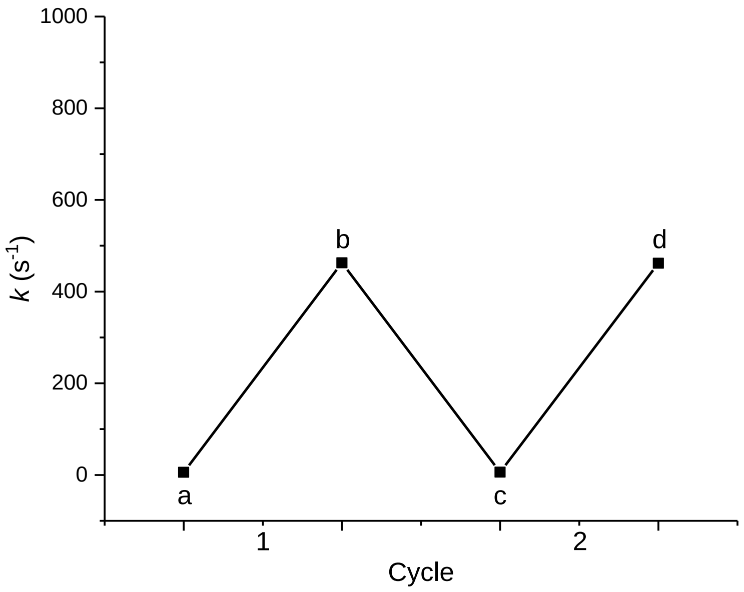

Supplementary Figure 46. Switching of rate for WP5 in D_2O at 298 K with multiple cycles shown in **Figure S37**.

3. Supplementary Tables

Supplementary Table 1. The summery of binding energy (kcal/mol) between cations and **WP5** anion in GS and TS_1.

	Li^+	Na^+	K^+	NH_4^+
E_{GS}	−493.42	−464.44	−461.67	−513.48
E_{TS}	−535.02	−499.05	−465.84	−515.28

The binding energy in TS_1 was higher than that in GS, which indicated stronger stabilizing effect of cations on TS_1 than GS.

Supplementary Table 2. Summary of calculated rotational barriers (kcal/mol) of **WP5-Li**, **WP5-Na**, **WP5-K** and **WP5-NH4**.

	$\Delta G^{\ddagger}_{TS1}$	$\Delta G^{\ddagger}_{TS2a}$	$\Delta G^{\ddagger}_{TS2b}$
**WP5-Li**	8.83	16.18	13.84
**WP5-Na**	6.59	21.08	9.65
**WP5-K**	3.03	17.28	8.38
**WP5-NH4**	3.94	11.64	6.02

Supplementary Table 3. The summery of kinetic parameters of **WP5-M** in different solvent.

Compound[a]	Solvent[b]	$\Delta H^{\ddagger}$ (kcal·mol^{-1})	$\Delta S^{\ddagger}$ (cal·mol^{-1}·K^{-1})	$\Delta G^{\ddagger}_{298K}$ (kcal·mol^{-1})
**WP5-Li**	D_2O	29.83	38.92	18.23
	D_2O/CD_3OD	31.09	42.99	18.28
**WP5-Na**	D_2O	21.32	12.67	17.55
	D_2O/CD_3OD	21.46	12.97	17.59
	D_2O/DMSO-d_6	21.39	12.87	17.55
**WP5-K**	D_2O	11.47	−15.04	15.95
	D_2O/CD_3OD	11.65	−14.46	15.96
**WP5-Rb**	D_2O	6.22	−31.74	15.68
	D_2O/CD_3OD	6.47	−31.02	15.71
**WP5-Cs**[c]	D_2O	5.04	−33.65	15.06
	D_2O/CD_3OD	-	-	-

(a). The concentration of rotors was 10.0 mM; (b). v/v of D_2O/CD_3OD = 2:1; v/v of D_2O/DMSO-d_6 = 2:1; (c). It was a failure to obtain kinetic parameters of **WP5-Cs** in mixed solvent since the limited solubility of **WP5-Cs**.

4. Supplementary Reference

1. Li, H.; Chen, D.-X.; Sun, Y.-L.; Zheng, Y. B.; Tan, L.-L.; Weiss, P. S.; Yang, Y.-W. Viologen-Mediated Assembly of and Sensing with Carboxylatopillar[5]arene-Modified Gold Nanoparticles. *J. Am. Chem. Soc.* **2013**, *135*, 1570–1576.

2. Ogoshi, T.; Hashizume, M.; Yamagishi, T.; Nakamoto, Y. Synthesis, conformational and host–guest properties of water-soluble pillar[5]arene. *Chem. Commun.* **2010**, *46*, 3708–3710.

3. Abraham, R. J.; Fisher, J.; Loftus, P. *Application of NMR Spectroscopy. Introduction to NMR Spectroscopy*, John Wiley & Sons Ltd.: New York, **1988**.

4. Connors, K. A. *Binding Constants*. John Wiley & Sons, Inc: New York, **1987**; Ashton, P. R., Ballardini, R., Balzani, V., Beˇlohradsky´, M., Gandolfi, M. T., Philp, D., Prodi, L., Raymo, F. M., Reddington, M. V., Spencer, N., Stoddart, J. F., Venturi, M., Williams. D. J. Self-Assembly, Spectroscopic, and Electrochemical Properties of [n]Rotaxanes. *J. Am. Chem. Soc.* **1996**, *118*, 4931-4951.

5. Frisch, M., Trucks, G., Schlegel, H., Scuseria, G., Robb, M., Cheeseman, J., Scalmani,

G., Barone, V., Mennucci, B., Petersson, G. et al., *Gaussian 16, Revision B.01, Gaussian, Inc., Wallingford CT* **2016**.

6. Stewart, J. J. P. Optimization of parameters for semiempirical methods V: modification of NDDO approximations and application to 70 elements. *J. Mol. Model.* **2007**, *13*, 1173-1213.

7. Řezáč, J.; Fanfrliék, J.; Salahub, D.; Hobza, P. Semiempirical Quantum Chemical PM6 Method Augmented by Dispersion and H-Bonding Correction Terms Reliably Describes Various Types of Noncovalent Complexes. *J. Chem. Theory Comput.* **2009**, *5*, 1749–1760.

8. Becke, A. D. Density-functional thermochemistry. III. The role of exact exchange. *J. Chem. Phys.* **1993**, *98*, 5648–5652.

9. Lee, C., Yang, W., Parr, R. G. Development of the Colle-Salvetti correlation-energy formula into a functional of the electron density. *Phys. Rev. B.* **1988**, *37*, 785–789.

10. Grimme, S., Antony, J., Ehrlich, S., Krieg, H. A consistent and accurate ab initio parametrization of density functional dispersion correction (DFT-D) for the 94 elements H-Pu. *J. Chem. Phys.* **2010**, *132*, 154104–154119.

11. Grimme, S., Ehrlich, S. Goerigk, L. Effect of the damping function in dispersion corrected density functional theory. *J. Comput. Chem.* **2011**, *32*, 1456–1465.
