# Supplementary material for: Cation controlled rotation in anionic pillar[5]arenes and its application for fluorescence switch
Source: Nat Commun. 2023 Feb 3;14:590. doi: 10.1038/s41467-023-36131-w (PMC9898256; doi:10.1038/s41467-023-36131-w)
Supplement: Supplementary file 3 — Description of Additional Supplementary Files [file 41467_2023_36131_MOESM3_ESM.pdf]

Description of Additional Supplementary Files

Supplementary Data 1: contains molecular coordination of calculated structures.

Supplementary Movie 1: a demo of how rotations is controlled by cations in WP5.
