# Supplementary material for: Cation controlled rotation in anionic pillar[5]arenes and its application for fluorescence switch
Source: Nat Commun. 2023 Feb 3;14:590. doi: 10.1038/s41467-023-36131-w (PMC9898256; doi:10.1038/s41467-023-36131-w)
Supplement: Supplementary file 4 — Supplementary Data 1 [file 41467_2023_36131_MOESM4_ESM.pdf]

Supplementary Data

Cation Controlled Rotation in Anionic Pillar[5]arenes and Its Application for Fluorescence Switch

Hao Zheng, [‡1] Lulu Fu, [‡1] Ranran Wang,[1] Jianmin Jiao,[1] Yingying Song,[1] Conghao Shi,[1] Yuan Chen,[1] Juli Jiang,*[1,2] Chen Lin,*[1] Jing Ma,*[1] and Leyong Wang[1]

[1] State Key Laboratory of Analytical Chemistry for Life Science, Jiangsu Key Laboratory of Advanced Organic Materials, School of Chemistry and Chemical Engineering, Nanjing University, 163 Xianlin Avenue, Nanjing 210023, China

[2] Ma'anShan High-Tech Research Institute of Nanjing University, Ma'anShan, 238200, China

Molecular coordinates of the calculated structures of WP5-M

WP5-Li – configuration with highest energy extracted from potential energy surface scanning

C	1.553	2.709	-3.142
C	0.845	3.654	-2.388
C	1.347	4.082	-1.152
C	2.562	3.573	-0.675
C	3.27	2.627	-1.429
C	2.762	2.191	-2.66
C	0.561	5.109	-0.316
H	1.243	5.694	0.266
H	0.007	5.752	-0.967
C	3.532	1.139	-3.481
H	4.58	1.233	-3.29
H	3.344	1.292	-4.523
C	-0.409	4.365	0.621
C	-1.708	4.066	0.19
C	0.009	3.985	1.903
C	-2.586	3.375	1.037
C	-0.868	3.295	2.75
C	-2.164	2.985	2.315
C	3.058	-0.268	-3.073
C	3.7	-0.949	-2.03
C	1.983	-0.867	-3.743
C	3.26	-2.223	-1.649
C	1.543	-2.142	-3.362
C	2.177	-2.818	-2.311
C	1.683	-4.212	-1.882
H	2.504	-4.778	-1.494
C	-3.123	2.212	3.239
H	-2.896	2.439	4.26
C	-3.893	-0.322	3.558
C	-3.7	-1.656	3.176
C	-2.597	-2.006	2.387
C	-1.679	-1.024	1.989

C	-1.872	0.31	2.372
C	-2.982	0.662	3.151
C	-2.429	-3.549	2.272
H	-2.116	-4.033	3.173
H	-3.352	-3.975	1.94
C	-1.354	-3.738	1.185
C	-1.724	-3.783	-0.166
C	-0.006	-3.864	1.547
C	-0.744	-3.941	-1.155
C	0.974	-4.022	0.558
C	0.605	-4.054	-0.794
H	1.267	-4.72	-2.727
H	-4.132	2.497	3.027
H	-0.079	4.049	-2.756
H	-3.578	3.146	0.707
H	-4.396	-2.407	3.486
H	-1.172	1.06	2.068
H	-1.026	-3.974	-2.187
H	0.276	-3.838	2.579
H	1.498	-0.351	-4.545
H	3.75	-2.743	-0.852
H	4.197	2.238	-1.064
H	0.999	4.221	2.235
O	1.04	2.271	-4.404
O	0.445	-2.753	-4.045
O	-3.1 -3.666	-0.534	
O	-5.02	0.036	4.363
O	-2.138	4.463	-1.115
O	-0.442	2.906	4.058
O	-0.547	-1.384	1.193
O	2.349	-4.149	0.927
O	4.805	-0.342	-1.354
O	3.08	4.018	0.582
C	4.508	3.963	0.555
H	4.824	2.957	0.376
H	4.875	4.597	-0.226
C	5.694	-1.359	-0.886
H	5.174	-2.006	-0.211
H	6.055	-1.927	-1.718

C	2.443	-4.769	2.212
H	1.933	-4.168	2.936
H	1.993	-5.739	2.174
C	0.969	2.671	4.05
H	1.196	1.891	3.354
H	1.478	3.567	3.76
C	-0.18	-0.28	0.361
H	-1	-0.028	-0.279
H	0.067	0.562	0.973
C	1.428	2.252	5.459
C	5.066	4.442	1.909
C	6.883	-0.706	-0.158
C	3.924	-4.905	2.61
C	1.039	-0.667	-0.497
O	8.009	-1.268	-0.161
O	4.292	-5.856	3.348
O	2.203	-0.458	-0.067
O	1.792	3.126	6.288
O	6.189	5.008	1.962
O	4.307	4.245	3.105
O	6.708	0.54	0.522
O	4.881	-3.952	2.14
O	0.849	-1.265	-1.782
O	1.442	0.87	5.825
C	0.283	3.326	-5.002
H	-0.531	3.591	-4.359
H	0.913	4.179	-5.149
C	0.414	-2.298	-5.401
H	0.297	-1.234	-5.417
H	1.329	-2.564	-5.887
C	-3.334	-4.394	-1.743
H	-2.72	-3.995	-2.524
H	-3.091	-5.425	-1.591
C	-5.377	-1.067	5.2
H	-5.627	-1.91	4.592
H	-4.551	-1.315	5.833
C	-3.11	3.533	-1.6
H	-2.675	2.557	-1.65
H	-3.95	3.517	-0.938

C	-4.816	-4.268	-2.14
C	-6.59	-0.681	6.067
C	-3.573	3.962	-3.005
C	-0.269	2.855	-6.361
C	-0.768	-2.956	-6.136
O	-0.695	-3.178	-7.373
O	0.406	3.03	-7.409
O	-4.557	4.735	-3.135
O	-6.738	-1.194	7.206
O	-5.38	-5.199	-2.771
O	-7.55	0.259	5.576
O	-5.552	-3.092	-1.791
O	-1.95	-3.314	-5.414
O	-1.548	2.22	-6.431
O	-2.885	3.48	-4.163
Li	-2.767	0.57	-2.96

WP5-Li - *pS*

C	-0.68	-4.102	1.662
C	-2.002	-3.643	1.519
C	-2.649	-3.572	0.274
C	-1.933	-4.036	-0.85
C	-0.558	-4.311	-0.727
C	0.105	-4.325	0.512
C	-4.095	-3.079	0.157
H	-4.546	-3.582	-0.705
H	-4.645	-3.379	1.053
C	1.599	-4.647	0.609
H	1.875	-5.307	-0.218
H	1.757	-5.193	1.546
C	-4.264	-1.565	-0.01
C	-4.625	-0.731	1.073
C	-4.116	-0.954	-1.265
C	-4.572	0.666	0.914
C	-4.248	0.433	-1.453
C	-4.359	1.279	-0.333
C	2.541	-3.438	0.581

C	3.365	-3.15	-0.535
C	2.661	-2.595	1.695
C	4.07	-1.933	-0.576
C	3.507	-1.474	1.709
C	4.143	-1.066	0.527
C	4.956	0.226	0.487
H	5.724	0.114	-0.284
C	-4.3 2.8	-0.471	
H	-4.774	3.065	-1.425
C	-2.477	4.333	0.533
C	-1.119	4.707	0.602
C	-0.174	4.312	-0.358
C	-0.646	3.541	-1.438
C	-1.956	3.044	-1.431
C	-2.887	3.394	-0.44
C	1.278	4.793	-0.282
H	1.509	5.314	-1.222
H	1.366	5.518	0.532
C	2.339	3.714	-0.079
C	2.899	3.42	1.184
C	2.859	3.014	-1.172
C	3.713	2.281	1.332
C	3.79	1.985	-1.034
C	4.149	1.505	0.238
H	5.471	0.314	1.449
H	-4.881	3.261	0.331
H	-2.564	-3.348	2.401
H	-4.72	1.309	1.776
H	-0.778	5.336	1.417
H	-2.29	2.38	-2.221
H	4.002	1.951	2.324
H	2.5 3.281	-2.16	
H	2.083	-2.834	2.584
H	4.6 -1.655	-1.482	
H	0.021	-4.545	-1.616
H	-3.901	-1.589	-2.12
O	-0.095	-4.343	2.892
O	3.697	-0.722	2.857
O	2.595	4.269	2.222

O	-3.441	4.861	1.376
O	-5.024	-1.341	2.248
O	-4.236	1.017	-2.709
O	0.238	3.328	-2.478
O	4.32	1.47	-2.243
O	3.454	-4.1 -1.536	
O	-2.63	-4.2 -2.033	
C	-2.207	-5.22	-2.944
H	-1.613	-4.766	-3.747
H	-1.559	-5.934	-2.419
C	4.608	-4.08	-2.379
H	4.44	-3.399	-3.225
H	5.464	-3.688	-1.812
C	5.753	1.541	-2.39
H	6.174	2.13	-1.569
H	6.167	0.528	-2.329
C	-5.004	0.406	-3.756
H	-4.311	0.008	-4.507
H	-5.57	-0.441	-3.35
C	-0.293	3.076	-3.777
H	-0.643	2.035	-3.852
H	-1.147	3.735	-3.969
C	-6.029	1.339	-4.489
C	-3.355	-6.065	-3.598
C	5.061	-5.465	-2.952
C	6.203	2.162	-3.736
C	0.787	3.305	-4.861
O	5.973	-5.338	-3.834
O	7.446	2.273	-3.888
O	0.396	3.793	-5.949
O	-6.299	2.467	-4.016
O	-2.941	-6.758	-4.584
O	-4.507	-6.045	-3.104
O	4.571	-6.527	-2.504
O	5.294	2.49	-4.56
O	1.976	2.944	-4.586
O	-6.521	0.79	-5.529
C	-0.913	-4.823	3.965
H	-1.11	-3.998	4.661

H	-1.881	-5.156	3.569
C	4.235	-1.385	4.01
H	3.668	-1.015	4.872
H	4.058	-2.465	3.939
C	3.557	4.493	3.261
H	3.178	4.047	4.187
H	4.499	3.988	3.013
C	-3.161	6.111	2.009
H	-2.545	5.949	2.905
H	-2.573	6.739	1.324
C	-5.872	-0.614	3.14
H	-5.26	-0.085	3.882
H	-6.431	0.145	2.576
C	3.911	5.996	3.537
C	-4.409	6.957	2.431
C	-6.945	-1.466	3.902
C	-0.326	-6.028	4.779
C	5.756	-1.166	4.316
O	6.103	-1.661	5.438
O	0.611	-6.708	4.299
O	-7.234	-2.617	3.499
O	-4.073	7.998	3.087
O	4.502	6.158	4.654
O	-5.562	6.604	2.094
O	3.654	6.864	2.67
O	6.476	-0.563	3.485
O	-0.946	-6.217	5.876
O	-7.473	-0.822	4.866
Li	3.518	2.299	-3.974

WP5-Li - TS_1

C	0.435	-3.084	-1.063
C	1.743	-2.596	-1.166
C	2.802	-3.099	-0.403
C	2.512	-4.172	0.47
C	1.173	-4.546	0.686
C	0.099	-4	-0.047
C	4.224	-2.557	-0.583

H	4.869	-3.05	0.149
H	4.581	-2.848	-1.579
C	-1.334	-4.461	0.264
H	-1.283	-5.128	1.132
H	-1.712	-5.058	-0.578
C	4.366	-1.044	-0.449
C	4.249	-0.181	-1.556
C	4.65	-0.455	0.795
C	4.194	1.204	-1.342
C	4.738	0.937	0.982
C	4.353	1.791	-0.077
C	-2.381	-3.397	0.579
C	-2.403	-2.759	1.83
C	-3.418	-3.083	-0.316
C	-3.265	-1.671	2.037
C	-4.405	-2.124	-0.028
C	-4.255	-1.303	1.113
C	-5.122	-0.062	1.349
H	-5.4	-0.013	2.407
C	4.017	3.265	0.124
H	4.375	3.593	1.103
C	1.993	4.857	0.356
C	0.601	5.064	0.293
C	-0.294	4.12	-0.248
C	0.261	2.932	-0.752
C	1.621	2.668	-0.549
C	2.516	3.594	-0.007
C	-1.779	4.445	-0.405
H	-1.961	5.418	0.077
H	-1.997	4.589	-1.468
C	-2.783	3.419	0.122
C	-3.849	2.966	-0.686
C	-2.705	2.905	1.423
C	-4.564	1.822	-0.292
C	-3.58	1.912	1.897
C	-4.416	1.243	0.981
H	-6.035	-0.161	0.755
H	4.562	3.877	-0.611
H	1.928	-1.812	-1.892

H	3.95	1.855	-2.175
H	0.181	6.001	0.646
H	2.006	1.713	-0.869
H	-5.254	1.353	-0.986
H	-1.923	3.281	2.079
H	-3.458	-3.616	-1.261
H	-3.171	-1.081	2.945
H	0.938	-5.271	1.459
H	4.793	-1.12	1.641
O	-0.502	-2.598	-1.958
O	-5.533	-1.943	-0.814
O	-4.116	3.675	-1.842
O	2.899	5.844	0.742
O	4.166	-0.749	-2.811
O	5.182	1.521	2.151
O	-0.567	2.087	-1.425
O	-3.628	1.529	3.227
O	-1.522	-3.198	2.8
O	3.584	-4.805	1.083
C	3.389	-6.128	1.58
H	2.869	-6.096	2.549
H	2.741	-6.684	0.888
C	-2.028	-3.458	4.115
H	-1.246	-3.128	4.808
H	-2.918	-2.849	4.31
C	-3.478	2.537	4.232
H	-2.531	2.365	4.758
H	-3.416	3.524	3.76
C	5.921	0.73	3.085
H	5.233	0.288	3.817
H	6.416	-0.096	2.555
C	-0.018	0.939	-2.08
H	0.546	0.32	-1.374
H	-0.887	0.35	-2.381
C	7.063	1.482	3.851
C	4.694	-6.971	1.77
C	-2.382	-4.942	4.475
C	-4.617	2.631	5.308
C	0.846	1.218	-3.343

O	-2.671	-5.083	5.71
O	-4.309	3.423	6.259
O	0.928	2.373	-3.804
O	7.613	2.477	3.321
O	4.452	-8.122	2.267
O	5.809	-6.508	1.433
O	-2.367	-5.822	3.583
O	-5.687	1.999	5.153
O	1.395	0.165	-3.811
O	7.377	0.912	4.946
C	-0.966	-3.518	-2.946
H	-1.152	-4.502	-2.502
H	-1.93	-3.119	-3.279
C	-5.991	-3.054	-1.587
H	-5.506	-3.048	-2.573
H	-5.698	-3.988	-1.088
C	-5.413	3.574	-2.427
H	-5.435	2.747	-3.149
H	-6.151	3.342	-1.645
C	2.39	7.151	0.978
H	1.713	7.435	0.157
H	1.797	7.163	1.903
C	4.55	0.077	-3.92
H	3.795	0.859	-4.073
H	5.526	0.533	-3.709
C	-5.924	4.87	-3.148
C	3.45	8.299	1.07
C	4.615	-0.759	-5.219
C	-0.1	-3.749	-4.221
C	-7.539	-3.142	-1.811
O	-7.848	-4.052	-2.65
O	-0.549	-4.667	-4.966
O	5.577	-0.503	-6.004
O	2.971	9.354	1.609
O	-6.946	4.648	-3.879
O	4.594	8.15	0.581
O	-5.371	5.974	-2.929
O	-8.319	-2.402	-1.167
O	0.913	-3.03	-4.428

O	3.672	-1.578	-5.405
Li	1.981	-1.504	-4.341

WP5-Li – TS_2a

C	-3.244	2.388	-1.711
C	-3.928	1.169	-1.75
C	-4.474	0.572	-0.608
C	-4.447	1.351	0.579
C	-3.644	2.506	0.635
C	-2.997	3.041	-0.497
C	-5.02	-0.852	-0.655
H	-5.647	-1.032	0.221
H	-5.682	-0.961	-1.527
C	-2.124	4.296	-0.381
H	-2.588	4.986	0.335
H	-2.094	4.791	-1.357
C	-3.934	-1.943	-0.73
C	-4.163	-3.267	-0.29
C	-2.686	-1.675	-1.299
C	-3.075	-4.162	-0.222
C	-1.669	-2.63	-1.403
C	-1.802	-3.868	-0.749
C	-0.679	4.087	0.073
C	-0.364	3.915	1.429
C	0.398	4.163	-0.829
C	0.95	3.592	1.804
C	1.738	4.039	-0.419
C	2.023	3.611	0.901
C	3.441	3.252	1.359
H	3.56	3.578	2.399
C	-0.68	-4.918	-0.707
H	-1.011	-5.728	-0.047
C	1.118	-4.453	1.086
C	2.319	-3.801	1.423
C	3.224	-3.318	0.463
C	2.937	-3.609	-0.889
C	1.669	-4.102	-1.222
C	0.711	-4.466	-0.264

C	4.489	-2.559	0.879
H	4.833	-2.98	1.835
H	5.274	-2.739	0.143
C	4.335	-1.04	1.057
C	5.027	-0.107	0.249
C	3.543	-0.513	2.088
C	4.659	1.252	0.309
C	3.37	0.866	2.293
C	3.807	1.766	1.303
H	4.149	3.81	0.739
H	-0.578	-5.338	-1.719
H	-4.009	0.656	-2.706
H	-3.208	-5.128	0.254
H	2.569	-3.669	2.472
H	1.39	-4.204	-2.263
H	5.059	1.948	-0.422
H	3.05	-1.207	2.764
H	0.176	4.357	-1.875
H	1.156	3.321	2.835
H	-3.48	3.012	1.579
H	-2.51	-0.688	-1.697
O	-2.82	2.923	-2.95
O	2.806	4.339	-1.242
O	6.047	-0.583	-0.549
O	0.321	-5.103	2.015
O	-5.464	-3.615	0.042
O	-0.529	-2.416	-2.13
O	3.926	-3.346	-1.803
O	2.747	1.377	3.416
O	-1.379	4.026	2.367
O	-5.213	0.906	1.621
C	-5.577	1.775	2.704
H	-5.011	1.471	3.59
H	-5.306	2.81	2.467
C	-1.208	4.981	3.424
H	-1.607	4.508	4.329
H	-0.141	5.168	3.593
C	3.066	0.818	4.697
H	2.117	0.555	5.179

H	3.639	-0.107	4.574
C	-0.386	-1.192	-2.836
H	-0.529	-0.334	-2.171
H	0.66	-1.183	-3.159
C	3.832	-3.753	-3.167
H	2.814	-3.628	-3.553
H	4.464	-3.036	-3.702
C	-1.271	-1.034	-4.106
C	-7.107	1.792	3.046
C	-1.919	6.368	3.267
C	3.872	1.723	5.692
C	4.344	-5.184	-3.563
O	-1.799	7.094	4.308
O	3.98	1.185	6.844
O	4.721	-5.984	-2.675
O	-1.675	-2.036	-4.716
O	-7.345	2.06	4.267
O	-7.936	1.633	2.117
O	-2.515	6.645	2.199
O	4.335	2.821	5.309
O	4.336	-5.363	-4.825
O	-1.45	0.192	-4.449
C	-3.725	3.9 -3.501	
H	-4.753	3.62	-3.238
H	-3.519	4.884	-3.062
C	2.593	5.246	-2.327
H	2.363	4.68	-3.239
H	1.725	5.881	-2.107
C	7.046	0.325	-1.016
H	6.768	0.699	-2.009
H	7.101	1.19	-0.341
C	0.951	-5.614	3.191
H	2.023	-5.756	2.997
H	0.86	-4.884	4.006
C	-5.772	-4.998	0.203
H	-5.201	-5.585	-0.532
H	-5.462	-5.339	1.2
C	8.502	-0.254	-1.09
C	0.425	-6.994	3.714

C	-7.273	-5.391	-0.009
C	-3.612	3.984	-5.042
C	3.773	6.228	-2.641
O	3.61	6.827	-3.755
O	-4.318	4.871	-5.589
O	-7.548	-6.537	0.48
O	0.877	-7.267	4.876
O	9.254	0.408	-1.88
O	-0.302	-7.711	2.989
O	8.829	-1.213	-0.351
O	4.696	6.391	-1.81
O	-2.846	3.145	-5.607
O	-8.034	-4.641	-0.665
Li	-2.066	1.842	-4.434

WP5-Li – TS_2b

C	3.386	-3.625	0.483
C	4.215	-2.51	0.276
C	3.995	-1.575	-0.75
C	2.943	-1.846	-1.642
C	2.003	-2.836	-1.316
C	2.175	-3.721	-0.242
C	4.886	-0.337	-0.847
H	5.734	-0.493	-0.169
H	5.32	-0.237	-1.847
C	1.128	-4.802	0.061
H	1.399	-5.296	0.996
H	1.182	-5.563	-0.733
C	4.202	0.978	-0.462
C	3.774	1.913	-1.411
C	4.007	1.32	0.887
C	3.023	3.03	-1.03
C	3.372	2.509	1.282
C	2.753	3.337	0.308
C	-0.317	-4.329	0.172
C	-0.828	-3.858	1.394
C	-1.215	-4.408	-0.908
C	-2.102	-3.269	1.43

C	-2.556	-3.986	-0.818
C	-2.98	-3.283	0.335
C	-4.355	-2.607	0.437
H	-4.82	-2.903	1.384
C	1.897	4.551	0.689
H	2.121	4.839	1.716
C	-0.507	4.502	1.637
C	-1.847	4.097	1.473
C	-2.395	3.738	0.231
C	-1.561	3.885	-0.899
C	-0.19	4.106	-0.708
C	0.38	4.362	0.549
C	-3.853	3.287	0.105
H	-4.426	3.788	0.9
H	-4.252	3.636	-0.849
C	-4.11	1.776	0.209
C	-4.716	1.038	-0.835
C	-3.819	1.074	1.388
C	-4.709	-0.37	-0.767
C	-4.043	-0.305	1.533
C	-4.347	-1.075	0.395
H	-4.979	-2.98	-0.379
H	2.2	5.388	0.043
H	5.088	-2.366	0.904
H	2.66	3.69	-1.814
H	-2.5	4.075	2.341
H	0.465	4.107	-1.571
H	-5.018	-0.948	-1.633
H	-3.41	1.628	2.229
H	-0.858	-4.847	-1.835
H	-2.44	-2.778	2.336
H	1.095	-2.931	-1.902
H	4.394	0.644	1.642
O	3.663	-4.643	1.354
O	-3.491	-4.256	-1.806
O	-5.302	1.747	-1.863
O	-0.025	5.06	2.81
O	4.169	1.782	-2.756
O	3.278	2.925	2.575

O	-2.152	3.761	-2.128
O	-3.926	-0.948	2.753
O	-0.014	-3.94	2.503
O	2.897	-1.142	-2.851
C	2.118	-1.743	-3.908
H	2.151	-2.831	-3.803
H	1.073	-1.423	-3.82
C	-0.562	-4.368	3.758
H	-0.051	-3.774	4.522
H	-1.63	-4.127	3.814
C	-4.489	-0.332	3.92
H	-3.713	-0.349	4.693
H	-4.727	0.718	3.716
C	4.106	2.394	3.613
H	3.555	2.625	4.53
H	4.174	1.302	3.549
C	-1.51	4.225	-3.316
H	-0.464	3.897	-3.359
H	-2.036	3.7 -4.119	
C	5.549	2.986	3.774
C	2.648	-1.392	-5.32
C	-0.4 -5.881	4.134	
C	-5.78	-0.983	4.527
C	-1.573	5.757	-3.654
O	-0.634	-6.102	5.368
O	-6.104	-0.447	5.639
O	-2.116	6.55	-2.852
O	6 3.761	2.9	
O	2.093	-2.009	-6.26
O	3.585	-0.541	-5.402
O	-0.102	-6.712	3.245
O	-6.369	-1.902	3.915
O	-1.05	6.018	-4.786
O	6.113	2.588	4.844
C	4.79	-4.64	2.227
H	5.098	-3.619	2.481
H	4.424	-5.098	3.151
C	-3.22	-5.343	-2.692
H	-2.586	-5.001	-3.521

H	-2.657	-6.117	-2.151
C	-6.274	1.101	-2.686
H	-5.781	0.657	-3.56
H	-6.747	0.284	-2.123
C	-0.968	5.64	3.712
H	-1.832	6.013	3.144
H	-1.339	4.875	4.407
C	5.235	2.678	-3.138
H	4.811	3.508	-3.718
H	5.696	3.092	-2.236
C	-7.448	2.014	-3.185
C	-0.456	6.858	4.555
C	6.328	1.97	-3.978
C	6.059	-5.456	1.793
C	-4.466	-6.064	-3.309
O	-4.126	-6.912	-4.201
O	6.94	-5.497	2.712
O	7.255	2.719	-4.393
O	-1.244	7.135	5.52
O	-8.038	1.536	-4.209
O	0.578	7.476	4.213
O	-7.757	3.041	-2.536
O	-5.619	-5.818	-2.889
O	6.102	-5.994	0.661
O	6.189	0.726	-4.159
Li	4.337	0.127	-3.846

WP5-Na – configuration with highest energy extracted from potential energy surface scanning

C	1.553	2.709	-3.142
C	0.845	3.654	-2.388
C	1.347	4.082	-1.152
C	2.562	3.573	-0.675
C	3.27	2.627	-1.429
C	2.762	2.191	-2.66
C	0.561	5.109	-0.316
H	1.243	5.694	0.266
H	0.007	5.752	-0.967

C	3.532	1.139	-3.481
H	4.58	1.233	-3.29
H	3.344	1.292	-4.523
C	-0.409	4.365	0.621
C	-1.708	4.066	0.19
C	0.009	3.985	1.903
C	-2.586	3.375	1.037
C	-0.868	3.295	2.75
C	-2.164	2.985	2.315
C	3.058	-0.268	-3.073
C	3.7	-0.949	-2.03
C	1.983	-0.867	-3.743
C	3.26	-2.223	-1.649
C	1.543	-2.142	-3.362
C	2.177	-2.818	-2.311
C	1.683	-4.212	-1.882
H	2.504	-4.778	-1.494
C	-3.123	2.212	3.239
H	-2.896	2.439	4.26
C	-3.893	-0.322	3.558
C	-3.7	-1.656	3.176
C	-2.597	-2.006	2.387
C	-1.679	-1.024	1.989
C	-1.872	0.31	2.372
C	-2.982	0.662	3.151
C	-2.429	-3.549	2.272
H	-2.116	-4.033	3.173
H	-3.352	-3.975	1.94
C	-1.354	-3.738	1.185
C	-1.724	-3.783	-0.166
C	-0.006	-3.864	1.547
C	-0.744	-3.941	-1.155
C	0.974	-4.022	0.558
C	0.605	-4.054	-0.794
H	1.267	-4.72	-2.727
H	-4.132	2.497	3.027
H	-0.079	4.049	-2.756
H	-3.578	3.146	0.707
H	-4.396	-2.407	3.486

H	-1.172	1.06	2.068
H	-1.026	-3.974	-2.187
H	0.276	-3.838	2.579
H	1.498	-0.351	-4.545
H	3.75	-2.743	-0.852
H	4.197	2.238	-1.064
H	0.999	4.221	2.235
O	1.04	2.271	-4.404
O	0.445	-2.753	-4.045
O	-3.1	-3.666	-0.534
O	-5.02	0.036	4.363
O	-2.138	4.463	-1.115
O	-0.442	2.906	4.058
O	-0.547	-1.384	1.193
O	2.349	-4.149	0.927
O	4.805	-0.342	-1.354
O	3.08	4.018	0.582
C	4.508	3.963	0.555
H	4.824	2.957	0.376
H	4.875	4.597	-0.226
C	5.694	-1.359	-0.886
H	5.174	-2.006	-0.211
H	6.055	-1.927	-1.718
C	2.443	-4.769	2.212
H	1.933	-4.168	2.936
H	1.993	-5.739	2.174
C	0.969	2.671	4.05
H	1.196	1.891	3.354
H	1.478	3.567	3.76
C	-0.18	-0.28	0.361
H	-1	-0.028	-0.279
H	0.067	0.562	0.973
C	1.428	2.252	5.459
C	5.066	4.442	1.909
C	6.883	-0.706	-0.158
C	3.924	-4.905	2.61
C	1.039	-0.667	-0.497
O	8.009	-1.268	-0.161
O	4.292	-5.856	3.348

O	2.203	-0.458	-0.067
O	1.792	3.126	6.288
O	6.189	5.008	1.962
O	4.307	4.245	3.105
O	6.708	0.54	0.522
O	4.881	-3.952	2.14
O	0.849	-1.265	-1.782
O	1.442	0.87	5.825
C	0.283	3.326	-5.002
H	-0.531	3.591	-4.359
H	0.913	4.179	-5.149
C	0.414	-2.298	-5.401
H	0.297	-1.234	-5.417
H	1.329	-2.564	-5.887
C	-3.334	-4.394	-1.743
H	-2.72	-3.995	-2.524
H	-3.091	-5.425	-1.591
C	-5.377	-1.067	5.2
H	-5.627	-1.91	4.592
H	-4.551	-1.315	5.833
C	-3.11	3.533	-1.6
H	-2.675	2.557	-1.65
H	-3.95	3.517	-0.938
C	-4.816	-4.268	-2.14
C	-6.59	-0.681	6.067
C	-3.573	3.962	-3.005
C	-0.269	2.855	-6.361
C	-0.768	-2.956	-6.136
O	-0.695	-3.178	-7.373
O	0.406	3.03	-7.409
O	-4.557	4.735	-3.135
O	-6.738	-1.194	7.206
O	-5.38	-5.199	-2.771
O	-7.55	0.259	5.576
O	-5.552	-3.092	-1.791
O	-1.95	-3.314	-5.414
O	-1.548	2.22	-6.431
O	-2.885	3.48	-4.163
Na	-2.767	0.57	-2.96

WP5-Na - *pS*

C	-4.369	-2.234	-1.088
C	-3.515	-3.35	-1.087
C	-2.446	-3.502	-0.179
C	-2.311	-2.499	0.799
C	-3.025	-1.299	0.658
C	-4.052	-1.121	-0.272
C	-1.497	-4.698	-0.342
H	-1.675	-5.441	0.449
H	-1.735	-5.181	-1.296
C	-4.846	0.188	-0.363
H	-5.639	0.169	0.399
H	-5.36	0.203	-1.327
C	-0.007	-4.373	-0.37
C	0.552	-3.735	-1.487
C	0.871	-4.747	0.66
C	1.879	-3.283	-1.441
C	2.244	-4.44	0.633
C	2.744	-3.584	-0.377
C	-4.034	1.468	-0.203
C	-3.653	1.975	1.046
C	-3.647	2.218	-1.329
C	-2.751	3.04	1.145
C	-2.894	3.403	-1.224
C	-2.311	3.752	0.02
C	-1.3 4.891	0.159	
H	-1.498	5.422	1.097
C	4.174	-3.033	-0.35
H	4.699	-3.501	0.487
C	4.414	-0.679	-1.357
C	4.341	0.717	-1.2
C	4.327	1.341	0.059
C	4.442	0.506	1.191
C	4.329	-0.888	1.034
C	4.287	-1.511	-0.224
C	4.265	2.866	0.187
H	4.768	3.138	1.121

H	4.822	3.316	-0.639
C	2.86	3.479	0.188
C	2.347	4.177	-0.93
C	2.045	3.416	1.329
C	0.993	4.563	-0.944
C	0.751	3.961	1.366
C	0.164	4.447	0.185
H	-1.448	5.584	-0.674
H	4.688	-3.327	-1.272
H	-3.646	-4.123	-1.837
H	2.263	-2.684	-2.263
H	4.315	1.353	-2.081
H	4.293	-1.522	1.916
H	0.565	4.986	-1.848
H	2.444	2.938	2.22
H	-3.958	1.866	-2.307
H	-2.384	3.342	2.121
H	-2.723	-0.471	1.295
H	0.469	-5.3 1.501	
O	-5.505	-2.136	-1.872
O	-2.703	4.263	-2.277
O	3.219	4.46	-1.965
O	4.601	-1.293	-2.582
O	-0.252	-3.518	-2.592
O	3.141	-4.95	1.559
O	4.669	1.112	2.413
O	0.006	4.001	2.533
O	-4.233	1.441	2.219
O	-1.446	-2.571	1.873
C	-1.597	-3.667	2.779
H	-1.715	-4.61	2.241
H	-0.645	-3.719	3.315
C	-5.322	2.272	2.682
H	-4.916	3.229	3.031
H	-5.986	2.482	1.834
C	2.761	-6.132	2.266
H	2.106	-5.871	3.11
H	2.181	-6.782	1.597
C	5.382	0.384	3.418

H	4.668	-0.084	4.107
H	5.959	-0.422	2.946
C	3.935	-7.003	2.827
C	-2.732	-3.6 3.841	
C	-6.168	1.64	3.816
C	6.404	1.222	4.262
O	-7.101	2.381	4.234
O	6.796	0.597	5.302
O	5.125	-6.729	2.55
O	-2.829	-4.64	4.545
O	-3.434	-2.552	3.938
O	-5.876	0.474	4.21
O	6.784	2.343	3.852
O	3.504	-7.981	3.525
C	-6.047	-3.338	-2.423
H	-5.44	-3.67	-3.276
H	-6.001	-4.135	-1.667
C	-3.608	4.215	-3.387
H	-3.191	3.568	-4.168
H	-4.56	3.774	-3.063
C	2.928	5.574	-2.814
H	2.319	5.242	-3.665
H	2.334	6.311	-2.256
C	5.361	-0.612	-3.586
H	4.676	-0.139	-4.301
H	5.952	0.187	-3.12
C	0.156	-4.111	-3.833
H	-0.131	-3.398	-4.613
H	1.248	-4.209	-3.862
C	4.174	6.343	-3.371
C	6.372	-1.504	-4.386
C	-0.462	-5.499	-4.214
C	-7.532	-3.233	-2.905
C	-3.974	5.599	-4.024
O	-4.548	5.465	-5.153
O	-8.156	-2.151	-2.809
O	-1.184	-6.108	-3.387
O	6.794	-0.929	-5.442
O	3.857	7.159	-4.298

O	6.716	-2.62	-3.932
O	5.306	6.159	-2.866
O	-3.754	6.657	-3.39
O	-7.962	-4.341	-3.366
O	-0.137	-5.869	-5.388
Na	-4.184	-0.663	3.253
C	0.594	4.608	3.693
H	1.647	4.843	3.496
H	0.57	3.868	4.502
C	-0.095	5.914	4.218
O	0.371	6.29	5.343
O	-0.984	6.468	3.529

WP5-Na - TS_1

C	0.811	-3.095	-1.003
C	2.057	-2.463	-1.036
C	3.1	-2.793	-0.167
C	2.867	-3.846	0.746
C	1.569	-4.374	0.878
C	0.506	-4	0.032
C	4.455	-2.09	-0.281
H	5.103	-2.46	0.517
H	4.916	-2.385	-1.232
C	-0.885	-4.621	0.231
H	-0.821	-5.32	1.072
H	-1.146	-5.205	-0.662
C	4.388	-0.567	-0.225
C	4.228	0.225	-1.369
C	4.491	0.113	1.004
C	3.969	1.597	-1.25
C	4.391	1.512	1.109
C	3.967	2.265	-0.019
C	-2.049	-3.677	0.517
C	-2.21	-3.095	1.784
C	-3.056	-3.43	-0.433
C	-3.19	-2.107	1.974
C	-4.153	-2.591	-0.173
C	-4.156	-1.805	1.003

C	-5.163	-0.672	1.227
H	-5.474	-0.674	2.278
C	3.49	3.711	0.09
H	3.824	4.135	1.04
C	1.307	5.045	0.46
C	-0.1	5.097	0.416
C	-0.885	4.13	-0.238
C	-0.201	3.089	-0.89
C	1.182	2.954	-0.711
C	1.965	3.896	-0.036
C	-2.401	4.282	-0.351
H	-2.688	5.192	0.197
H	-2.659	4.461	-1.4
C	-3.26	3.110.128	
C	-4.273	2.565	-0.692
C	-3.101	2.55	1.403
C	-4.839	1.325	-0.344
C	-3.845	1.439	1.838
C	-4.608	0.715	0.902
H	-6.042	-0.864	0.604
H	3.974	4.312	-0.694
H	2.214	-1.712	-1.798
H	3.726	2.144	-2.159
H	-0.621	5.929	0.881
H	1.673	2.096	-1.142
H	-5.478	0.807	-1.052
H	-2.363	2.988	2.07
H	-2.979	-3.921	-1.398
H	-3.209	-1.546	2.904
H	1.358	-5.086	1.669
H	4.65	-0.484	1.895
O	-0.107	-2.777	-1.989
O	-5.248	-2.499	-1.017
O	-4.643	3.289	-1.81
O	2.097	6.083	0.947
O	4.318	-0.334	-2.653
O	4.687	2.207	2.255
O	-0.938	2.251	-1.669
O	-3.823	0.994	3.149

O	-1.345	-3.48	2.792
O	3.953	-4.306	1.476
C	3.893	-5.627	2.013
H	3.307	-5.631	2.943
H	3.371	-6.285	1.303
C	-1.901	-3.856	4.058
H	-1.218	-3.461	4.818
H	-2.873	-3.372	4.207
C	-3.829	1.966	4.201
H	-2.898	1.855	4.772
H	-3.834	2.976	3.775
C	5.415	1.566	3.309
H	4.707	1.184	4.053
H	5.975	0.713	2.907
C	-0.259	1.3 -2.491	
H	0.382	0.65	-1.885
H	-1.058	0.67	-2.892
C	6.484	2.461	4.027
C	5.281	-6.283	2.321
C	-2.086	-5.387	4.341
C	-5.024	1.903	5.216
C	0.549	1.877	-3.695
O	-2.441	-5.616	5.545
O	-4.848	2.685	6.209
O	0.31	3.034	-4.099
O	7.038	3.386	3.387
O	5.157	-7.44	2.844
O	6.345	-5.684	2.038
O	-1.894	-6.219	3.424
O	-6.012	1.168	4.984
O	1.372	1.038	-4.19
O	6.744	2.063	5.208
C	-0.278	-3.759	-3.014
H	-0.234	-4.768	-2.586
H	-1.293	-3.605	-3.398
C	-5.533	-3.614	-1.864
H	-5.014	-3.495	-2.824
H	-5.148	-4.531	-1.398
C	-5.918	3.044	-2.399

H	-5.834	2.256	-3.159
H	-6.615	2.68	-1.629
C	1.449	7.306	1.284
H	0.717	7.559	0.502
H	0.891	7.19	2.224
C	5.646	-0.229	-3.196
H	6.008	0.798	-3.053
H	6.318	-0.898	-2.639
C	-6.601	4.293	-3.057
C	2.372	8.563	1.419
C	5.742	-0.566	-4.707
C	0.688	-3.732	-4.238
C	-7.048	-3.881	-2.162
O	-7.206	-4.764	-3.07
O	0.495	-4.694	-5.04
O	6.929	-0.527	-5.157
O	1.799	9.514	2.052
O	-7.574	3.972	-3.816
O	3.498	8.584	0.872
O	-6.213	5.45	-2.765
O	-7.939	-3.293	-1.506
O	1.523	-2.798	-4.348
O	4.684	-0.822	-5.335
Na	2.564	-0.821	-4.245

WP5-Na – TS_2a

C	-3.419	2.242	-1.558
C	-3.977	0.964	-1.668
C	-4.43	0.238	-0.56
C	-4.428	0.923	0.682
C	-3.763	2.157	0.8
C	-3.213	2.834	-0.304
C	-4.888	-1.21	-0.698
H	-5.55	-1.463	0.131
H	-5.491	-1.305	-1.614
C	-2.46	4.157	-0.125
H	-2.959	4.753	0.648
H	-2.508	4.715	-1.066

C	-3.748	-2.243	-0.758
C	-3.881	-3.544	-0.222
C	-2.547	-1.946	-1.413
C	-2.741	-4.37	-0.144
C	-1.48	-2.848	-1.494
C	-1.514	-4.041	-0.748
C	-0.989	4.042	0.275
C	-0.618	3.843	1.613
C	0.049	4.22	-0.658
C	0.727	3.6	1.935
C	1.408	4.171	-0.295
C	1.765	3.724	1
C	3.219	3.456	1.406
H	3.352	3.783	2.444
C	-0.323	-5.01	-0.684
H	-0.594	-5.824	-0.002
C	1.438	-4.386	1.095
C	2.59	-3.646	1.421
C	3.458	-3.113	0.452
C	3.191	-3.445	-0.895
C	1.961	-4.031	-1.219
C	1.032	-4.449	-0.254
C	4.668	-2.263	0.854
H	5.056	-2.661	1.804
H	5.454	-2.382	0.107
C	4.407	-0.76	1.044
C	4.999	0.224	0.218
C	3.615	-0.297	2.105
C	4.534	1.552	0.299
C	3.35	1.065	2.325
C	3.685	1.999	1.327
H	3.866	4.065	0.767
H	-0.193	-5.446	-1.685
H	-4.054	0.517	-2.659
H	-2.796	-5.306	0.404
H	2.829	-3.481	2.467
H	1.691	-4.174	-2.258
H	4.858	2.279	-0.44
H	3.201	-1.027	2.795

H	-0.223	4.442	-1.686
H	0.987	3.309	2.948
H	-3.635	2.619	1.772
H	-2.457	-0.982	-1.895
O	-3.068	2.901	-2.758
O	2.425	4.564	-1.143
O	6.023	-0.172	-0.619
O	0.69	-5.076	2.034
O	-5.144	-3.943	0.188
O	-0.378	-2.641	-2.278
O	4.159	-3.126	-1.815
O	2.733	1.526	3.474
O	-1.609	3.841	2.582
O	-5.043	0.288	1.723
C	-5.536	0.982	2.876
H	-5.289	0.341	3.727
H	-5.017	1.935	3.021
C	-1.458	4.729	3.701
H	-1.816	4.175	4.576
H	-0.398	4.953	3.863
C	3.121	0.973	4.738
H	2.204	0.643	5.241
H	3.75	0.089	4.587
C	-0.267	-1.444	-3.029
H	-0.473	-0.57	-2.4
H	0.79	-1.394	-3.313
C	4.098	-3.565	-3.171
H	3.073	-3.528	-3.558
H	4.671	-2.812	-3.72
C	-1.102	-1.366	-4.34
C	-7.078	1.27	2.914
C	-2.234	6.089	3.651
C	3.894	1.916	5.724
C	4.72	-4.959	-3.539
O	-2.147	6.735	4.747
O	4.061	1.375	6.867
O	5.151	-5.713	-2.637
O	-1.508	-2.405	-4.888
O	-7.503	1.486	4.096

O	-7.724	1.301	1.842
O	-2.838	6.422	2.605
O	4.284	3.043	5.341
O	4.732	-5.16	-4.799
O	-1.233	-0.165	-4.777
C	-4.101	3.76	-3.277
H	-5.076	3.291	-3.093
H	-4.084	4.716	-2.738
C	2.115	5.475	-2.201
H	1.878	4.913	-3.114
H	1.223	6.056	-1.931
C	6.934	0.809	-1.116
H	6.598	1.158	-2.1
H	6.944	1.678	-0.443
C	1.359	-5.54	3.209
H	2.438	-5.601	3.014
H	1.215	-4.818	4.024
C	-5.371	-5.331	0.426
H	-4.778	-5.924	-0.286
H	-5.031	-5.599	1.435
C	8.428	0.344	-1.233
C	0.937	-6.955	3.733
C	-6.851	-5.816	0.257
C	-3.973	4.032	-4.8
C	3.224	6.527	-2.545
O	2.99	7.122	-3.648
O	-4.817	4.854	-5.251
O	-7.059	-6.943	0.817
O	1.412	-7.195	4.893
O	9.1	1.054	-2.052
O	0.261	-7.722	3.01
O	8.851	-0.579	-0.498
O	4.16	6.741	-1.74
O	-3.081	3.41	-5.449
O	-7.656	-5.15	-0.435
Na	-1.877	1.824	-4.367

WP5-Na – TS_2b

C	1.061	-4.725	0.593
C	2.335	-4.176	0.37
C	2.557	-3.109	-0.522
C	1.452	-2.64	-1.25
C	0.162	-3.08	-0.911
C	-0.074	-4.101	0.016
C	3.95	-2.521	-0.722
H	4.678	-3.215	-0.278
H	4.159	-2.465	-1.795
C	-1.501	-4.575	0.339
H	-1.488	-5.072	1.311
H	-1.781	-5.342	-0.4
C	4.158	-1.122	-0.131
C	4.618	-0.055	-0.916
C	3.879	-0.83	1.216
C	4.55	1.259	-0.442
C	3.962	0.471	1.74
C	4.152	1.566	0.862
C	-2.583	-3.501	0.359
C	-2.891	-2.802	1.542
C	-3.351	-3.195	-0.78
C	-3.728	-1.676	1.476
C	-4.334	-2.188	-0.791
C	-4.435	-1.318	0.318
C	-5.293	-0.046	0.298
H	-5.869	0.009	1.229
C	3.834	3	1.259
H	3.915	3.112	2.343
C	1.798	4.584	1.391
C	0.464	4.869	1.038
C	-0.225	4.176	0.026
C	0.492	3.19	-0.676
C	1.766	2.813	-0.231
C	2.437	3.466	0.809
C	-1.653	4.548	-0.37
H	-1.934	5.447	0.2
H	-1.668	4.83	-1.426
C	-2.724	3.472	-0.169
C	-3.58	3.077	-1.22

C	-2.927	2.867	1.079
C	-4.355	1.912	-1.066
C	-3.881	1.856	1.285
C	-4.493	1.253	0.169
H	-5.996	-0.119	-0.537
H	4.586	3.677	0.826
H	3.201	-4.615	0.857
H	4.762	2.063	-1.143
H	-0.067	5.666	1.55
H	2.266	1.993	-0.726
H	-4.879	1.499	-1.923
H	-2.313	3.195	1.914
H	-3.181	-3.781	-1.679
H	-3.841	-1.038	2.347
H	-0.693	-2.601	-1.378
H	3.546	-1.643	1.855
O	0.831	-5.872	1.318
O	-5.223	-2.015	-1.843
O	-3.604	3.866	-2.354
O	2.54	5.358	2.279
O	5.092	-0.259	-2.216
O	3.771	0.765	3.065
O	-0.119	2.638	-1.761
O	-4.204	1.386	2.546
O	-2.291	-3.225	2.707
O	1.66	-1.721	-2.262
C	1.012	-1.987	-3.524
H	0.144	-2.634	-3.38
H	0.666	-1.014	-3.889
C	-3.004	-3.172	3.95
H	-2.282	-2.825	4.696
H	-3.811	-2.432	3.905
C	-4.356	2.326	3.617
H	-3.708	1.992	4.436
H	-4.006	3.315	3.304
C	4.167	-0.144	4.099
H	3.429	-0.009	4.895
H	4.094	-1.184	3.764
C	0.604	1.735	-2.598

H	1.044	0.919	-2.013
H	-0.168	1.29	-3.234
C	5.592	0.057	4.724
C	1.919	-2.602	-4.633
C	-3.632	-4.511	4.475
C	-5.799	2.522	4.202
C	1.684	2.351	-3.54
O	-3.966	-4.439	5.704
O	-5.792	3.256	5.246
O	1.765	3.589	-3.672
O	6.44	0.754	4.121
O	1.306	-3.275	-5.506
O	3.153	-2.32	-4.609
O	-3.779	-5.478	3.693
O	-6.787	2.004	3.634
O	2.369	1.457	-4.142
O	5.729	-0.572	5.823
C	1.773	-6.399	2.252
H	2.577	-5.684	2.453
H	1.221	-6.526	3.191
C	-5.488	-3.144	-2.677
H	-4.744	-3.195	-3.484
H	-5.387	-4.064	-2.085
C	-4.74	3.804	-3.214
H	-4.585	3.038	-3.984
H	-5.625	3.507	-2.632
C	1.991	6.607	2.686
H	1.558	7.118	1.813
H	1.172	6.445	3.402
C	6.513	-0.419	-2.31
H	7.004	0.345	-1.693
H	6.798	-1.404	-1.909
C	-5.115	5.15	-3.926
C	2.994	7.624	3.328
C	7.041	-0.294	-3.765
C	2.42	-7.791	1.923
C	-6.91	-3.199	-3.332
O	-7.003	-4.146	-4.183
O	3.136	-8.226	2.885

O	8.301	-0.431	-3.863
O	2.39	8.6	3.889
O	-5.925	4.977	-4.896
O	4.231	7.463	3.209
O	-4.675	6.239	-3.487
O	-7.808	-2.403	-2.974
O	2.19	-8.352	0.826
O	6.213	-0.069	-4.684
Na	3.823	-0.216	-4.093

WP5-K – configuration with highest energy extracted from potential energy surface scanning

C	1.553	2.709	-3.142
C	0.845	3.654	-2.388
C	1.347	4.082	-1.152
C	2.562	3.573	-0.675
C	3.27	2.627	-1.429
C	2.762	2.191	-2.66
C	0.561	5.109	-0.316
H	1.243	5.694	0.266
H	0.007	5.752	-0.967
C	3.532	1.139	-3.481
H	4.58	1.233	-3.29
H	3.344	1.292	-4.523
C	-0.409	4.365	0.621
C	-1.708	4.066	0.19
C	0.009	3.985	1.903
C	-2.586	3.375	1.037
C	-0.868	3.295	2.75
C	-2.164	2.985	2.315
C	3.058	-0.268	-3.073
C	3.7	-0.949	-2.03
C	1.983	-0.867	-3.743
C	3.26	-2.223	-1.649
C	1.543	-2.142	-3.362
C	2.177	-2.818	-2.311
C	1.683	-4.212	-1.882
H	2.504	-4.778	-1.494

C	-3.123	2.212	3.239
H	-2.896	2.439	4.26
C	-3.893	-0.322	3.558
C	-3.7	-1.656	3.176
C	-2.597	-2.006	2.387
C	-1.679	-1.024	1.989
C	-1.872	0.31	2.372
C	-2.982	0.662	3.151
C	-2.429	-3.549	2.272
H	-2.116	-4.033	3.173
H	-3.352	-3.975	1.94
C	-1.354	-3.738	1.185
C	-1.724	-3.783	-0.166
C	-0.006	-3.864	1.547
C	-0.744	-3.941	-1.155
C	0.974	-4.022	0.558
C	0.605	-4.054	-0.794
H	1.267	-4.72	-2.727
H	-4.132	2.497	3.027
H	-0.079	4.049	-2.756
H	-3.578	3.146	0.707
H	-4.396	-2.407	3.486
H	-1.172	1.06	2.068
H	-1.026	-3.974	-2.187
H	0.276	-3.838	2.579
H	1.498	-0.351	-4.545
H	3.75	-2.743	-0.852
H	4.197	2.238	-1.064
H	0.999	4.221	2.235
O	1.04	2.271	-4.404
O	0.445	-2.753	-4.045
O	-3.1	-3.666	-0.534
O	-5.02	0.036	4.363
O	-2.138	4.463	-1.115
O	-0.442	2.906	4.058
O	-0.547	-1.384	1.193
O	2.349	-4.149	0.927
O	4.805	-0.342	-1.354
O	3.08	4.018	0.582

C	4.508	3.963	0.555
H	4.824	2.957	0.376
H	4.875	4.597	-0.226
C	5.694	-1.359	-0.886
H	5.174	-2.006	-0.211
H	6.055	-1.927	-1.718
C	2.443	-4.769	2.212
H	1.933	-4.168	2.936
H	1.993	-5.739	2.174
C	0.969	2.671	4.05
H	1.196	1.891	3.354
H	1.478	3.567	3.76
C	-0.18	-0.28	0.361
H	-1	-0.028	-0.279
H	0.067	0.562	0.973
C	1.428	2.252	5.459
C	5.066	4.442	1.909
C	6.883	-0.706	-0.158
C	3.924	-4.905	2.61
C	1.039	-0.667	-0.497
O	8.009	-1.268	-0.161
O	4.292	-5.856	3.348
O	2.203	-0.458	-0.067
O	1.792	3.126	6.288
O	6.189	5.008	1.962
O	4.307	4.245	3.105
O	6.708	0.54	0.522
O	4.881	-3.952	2.14
O	0.849	-1.265	-1.782
O	1.442	0.87	5.825
C	0.283	3.326	-5.002
H	-0.531	3.591	-4.359
H	0.913	4.179	-5.149
C	0.414	-2.298	-5.401
H	0.297	-1.234	-5.417
H	1.329	-2.564	-5.887
C	-3.334	-4.394	-1.743
H	-2.72	-3.995	-2.524
H	-3.091	-5.425	-1.591

C	-5.377	-1.067	5.2
H	-5.627	-1.91	4.592
H	-4.551	-1.315	5.833
C	-3.11	3.533	-1.6
H	-2.675	2.557	-1.65
H	-3.95	3.517	-0.938
C	-4.816	-4.268	-2.14
C	-6.59	-0.681	6.067
C	-3.573	3.962	-3.005
C	-0.269	2.855	-6.361
C	-0.768	-2.956	-6.136
O	-0.695	-3.178	-7.373
O	0.406	3.03	-7.409
O	-4.557	4.735	-3.135
O	-6.738	-1.194	7.206
O	-5.38	-5.199	-2.771
O	-7.55	0.259	5.576
O	-5.552	-3.092	-1.791
O	-1.95	-3.314	-5.414
O	-1.548	2.22	-6.431
O	-2.885	3.48	-4.163
K	-2.853	1.322	-2.965

WP5-K – *pS*

C	-0.138	-4.21	1.858
C	-1.513	-3.903	1.808
C	-2.173	-3.573	0.609
C	-1.401	-3.602	-0.565
C	-0.015	-3.768	-0.493
C	0.659	-4.023	0.707
C	-3.671	-3.246	0.606
H	-4.132	-3.712	-0.271
H	-4.124	-3.686	1.501
C	2.181	-4.173	0.717
H	2.456	-4.834	-0.116
H	2.47	-4.678	1.642
C	-4.022	-1.761	0.585
C	-4.001	-0.998	1.766

C	-4.429	-1.117	-0.594
C	-4.109	0.399	1.678
C	-4.744	0.254	-0.634
C	-4.442	1.06	0.486
C	2.985	-2.876	0.585
C	3.111	-2.187	-0.634
C	3.693	-2.355	1.685
C	3.762	-0.949	-0.685
C	4.472	-1.185	1.598
C	4.428	-0.4 0.42	
C	5.116	0.966	0.318
H	5.87	0.925	-0.477
C	-4.45	2.581	0.42
H	-5.176	2.895	-0.341
C	-2.673	4.432	0.66
C	-1.375	4.91	0.389
C	-0.509	4.283	-0.519
C	-0.995	3.148	-1.201
C	-2.229	2.593	-0.829
C	-3.087	3.208	0.095
C	0.871	4.87	-0.832
H	0.899	5.117	-1.902
H	0.977	5.802	-0.265
C	2.083	3.988	-0.537
C	2.615	3.873	0.765
C	2.745	3.301	-1.566
C	3.557	2.862	1.027
C	3.845	2.462	-1.324
C	4.16	2.112	0.003
H	5.633	1.159	1.261
H	-4.794	2.992	1.375
H	-2.098	-3.911	2.722
H	-3.928	1.003	2.563
H	-1.014	5.799	0.897
H	-2.559	1.663	-1.274
H	3.846	2.65	2.053
H	2.374	3.41	-2.581
H	3.637	-2.893	2.626
H	3.775	-0.408	-1.628

H	0.572	-3.73	-1.405
H	-4.501	-1.707	-1.503
O	0.483	-4.704	2.986
O	5.302	-0.757	2.616
O	2.167	4.765	1.719
O	-3.574	5.106	1.48
O	-3.821	-1.662	2.964
O	-5.321	0.863	-1.731
O	-0.224	2.685	-2.232
O	4.62	1.929	-2.339
O	2.628	-2.754	-1.819
O	-1.973	-3.499	-1.839
C	-2.443	-4.771	-2.33
H	-1.711	-5.545	-2.057
H	-3.392	-5.026	-1.841
C	3.659	-3.501	-2.492
H	4.474	-2.817	-2.77
H	4.077	-4.243	-1.798
C	4.889	2.757	-3.482
H	4.277	2.408	-4.322
H	4.583	3.787	-3.263
C	-6.192	0.073	-2.552
H	-5.619	-0.368	-3.378
H	-6.595	-0.756	-1.956
C	-0.632	1.517	-2.94
H	-0.399	0.632	-2.336
H	-1.717	1.536	-3.11
C	-7.419	0.83	-3.164
C	-2.641	-4.849	-3.871
C	3.191	-4.24	-3.773
C	6.383	2.818	-3.95
C	0.014	1.39	-4.348
O	4.075	-5.018	-4.248
O	6.525	3.527	-5.002
O	0.026	0.188	-4.768
O	-7.633	2.031	-2.879
O	-3.293	-5.877	-4.233
O	-2.123	-3.963	-4.593
O	2.043	-4.006	-4.225

O	7.279	2.229	-3.301
O	0.368	2.425	-4.956
O	-8.116	0.072	-3.917
C	-0.29	-5.447	3.934
H	-0.616	-4.782	4.743
H	-1.193	-5.836	3.446
C	5.709	-1.705	3.604
H	4.924	-1.816	4.365
H	5.842	-2.689	3.132
C	3.028	5.103	2.81
H	2.678	4.59	3.714
H	4.045	4.749	2.602
C	-3.405	6.518	1.616
H	-2.527	6.736	2.243
H	-3.21	6.956	0.628
C	-4.568	-1.243	4.112
H	-3.849	-1.063	4.92
H	-5.079	-0.296	3.907
C	3.161	6.632	3.13
C	-4.611	7.281	2.256
C	-5.655	-2.238	4.65
C	0.44	-6.682	4.565
C	7.053	-1.374	4.338
O	7.276	-2.175	5.304
O	1.462	-7.153	4.012
O	-5.978	-3.239	3.971
O	-4.523	8.544	2.094
O	3.679	6.842	4.276
O	-5.5	6.655	2.88
O	2.831	7.482	2.27
O	7.783	-0.441	3.932
O	-0.162	-7.126	5.597
O	-6.126	-1.866	5.775
K	-0.026	-2.31	-3.842

WP5-K – TS_1

C	1.362	-3.162	-0.983
C	2.516	-2.373	-1.005

C	3.478	-2.39	0.01
C	3.279	-3.313	1.06
C	2.048	-3.992	1.159
C	1.051	-3.915	0.167
C	4.715	-1.492	-0.088
H	5.375	-1.722	0.752
H	5.256	-1.75	-1.007
C	-0.27	-4.677	0.345
H	-0.158	-5.352	1.201
H	-0.448	-5.297	-0.542
C	4.407	0.005	-0.108
C	4.185	0.717	-1.296
C	4.329	0.733	1.094
C	3.727	2.04	-1.251
C	3.972	2.091	1.136
C	3.549	2.741	-0.052
C	-1.532	-3.853	0.589
C	-1.791	-3.291	1.849
C	-2.532	-3.716	-0.39
C	-2.885	-2.424	2.013
C	-3.726	-3.009	-0.159
C	-3.855	-2.236	1.018
C	-5.009	-1.249	1.224
H	-5.332	-1.295	2.271
C	2.927	4.135	-0.037
H	3.239	4.664	0.865
C	0.627	5.194	0.475
C	-0.779	5.094	0.46
C	-1.467	4.1 -0.256	
C	-0.692	3.2 -1.01	
C	0.702	3.207	-0.874
C	1.391	4.168	-0.124
C	-2.993	4.068	-0.307
H	-3.364	4.909	0.298
H	-3.32	4.257	-1.334
C	-3.671	2.776	0.153
C	-4.59	2.094	-0.674
C	-3.431	2.235	1.424
C	-4.967	0.782	-0.336

C	-4.005	1.024	1.85
C	-4.652	0.203	0.906
H	-5.844	-1.561	0.59
H	3.331	4.708	-0.886
H	2.691	-1.745	-1.869
H	3.487	2.526	-2.195
H	-1.374	5.82	1.006
H	1.282	2.453	-1.389
H	-5.524	0.182	-1.049
H	-2.768	2.773	2.096
H	-2.376	-4.195	-1.351
H	-2.996	-1.876	2.945
H	1.836	-4.59	2.04
H	4.56	0.205	2.013
O	0.55	-3.151	-2.109
O	-4.8	-3.04	-1.032
O	-5.059	2.763	-1.789
O	1.309	6.272	1.032
O	4.41	0.133	-2.553
O	3.955	2.851	2.273
O	-1.372	2.355	-1.834
O	-3.925	0.58	3.158
O	-0.918	-3.574	2.883
O	4.323	-3.502	1.946
C	4.399	-4.739	2.659
H	3.816	-4.672	3.587
H	3.948	-5.536	2.052
C	-1.464	-4.037	4.125
H	-0.855	-3.579	4.912
H	-2.49	-3.672	4.249
C	-4.052	1.536	4.217
H	-3.115	1.538	4.787
H	-4.184	2.541	3.798
C	4.58	2.455	3.496
H	3.922	2.841	4.281
H	4.603	1.366	3.605
C	-0.653	1.488	-2.706
H	0.093	0.911	-2.146
H	-1.406	0.777	-3.058

C	6.021	3.009	3.781
C	5.848	-5.21	3.021
C	-1.476	-5.584	4.378
C	-5.231	1.318	5.229
C	0.012	2.133	-3.964
O	-1.857	-5.877	5.559
O	-5.154	2.111	6.226
O	0.772	1.313	-4.579
O	6.692	3.502	2.846
O	5.846	-6.202	3.822
O	6.842	-4.65	2.5
O	-1.137	-6.368	3.461
O	-6.119	0.468	4.991
O	-0.268	3.306	-4.284
O	6.354	2.87	5.003
C	0.533	-4.358	-2.884
H	0.98	-5.177	-2.308
H	-0.519	-4.611	-3.062
C	-4.946	-4.185	-1.876
H	-4.434	-4.01	-2.831
H	-4.463	-5.05	-1.401
C	-6.3 2.359	-2.365	
H	-6.123	1.604	-3.141
H	-6.928	1.891	-1.593
C	0.544	7.398	1.447
H	-0.225	7.616	0.69
H	0.018	7.172	2.385
C	5.738	0.394	-3.047
H	5.965	1.459	-2.902
H	6.463	-0.179	-2.453
C	-7.154	3.516	-2.989
C	1.334	8.735	1.644
C	5.955	0.063	-4.551
C	1.241	-4.326	-4.274
C	-6.419	-4.623	-2.185
O	-6.469	-5.517	-3.094
O	1.196	-5.443	-4.873
O	7.155	0.242	-4.928
O	0.661	9.596	2.306

O	-8.085	3.082	-3.744
O	2.46	8.892	1.117
O	-6.922	4.709	-2.677
O	-7.374	-4.141	-1.534
O	1.76	-3.256	-4.682
O	4.977	-0.317	-5.242
K	2.389	-0.714	-4.32

WP5-K – TS_2a

C	2.574	-2.759	-1.791
C	3.488	-1.699	-1.793
C	4.291	-1.38	-0.692
C	4.272	-2.308	0.385
C	3.278	-3.306	0.424
C	2.39	-3.542	-0.644
C	5.097	-0.084	-0.669
H	5.81	-0.112	0.157
H	5.704	-0.017	-1.585
C	1.33	-4.643	-0.567
H	1.663	-5.406	0.145
H	1.252	-5.112	-1.555
C	4.251	1.201	-0.549
C	4.755	2.376	0.064
C	2.966	1.298	-1.089
C	3.876	3.442	0.332
C	2.158	2.435	-0.984
C	2.553	3.495	-0.152
C	-0.075	-4.22	-0.144
C	-0.392	-4.037	1.211
C	-1.126	-4.103	-1.072
C	-1.657	-3.543	1.57
C	-2.443	-3.792	-0.685
C	-2.699	-3.384	0.645
C	-4.06	-2.837	1.083
H	-4.293	-3.222	2.08
C	1.659	4.682	0.237
H	2.089	5.128	1.142
C	-0.154	3.588	1.631

C	-1.478	3.149	1.77
C	-2.518	3.61	0.952
C	-2.199	4.621	0.011
C	-0.85	4.929	-0.23
C	0.196	4.382	0.531
C	-3.949	3.073	1.117
H	-4.358	3.455	2.065
H	-4.567	3.481	0.316
C	-4.094	1.553	1.117
C	-4.309	0.836	-0.077
C	-4.073	0.813	2.312
C	-4.248	-0.567	-0.056
C	-4.182	-0.59	2.347
C	-4.153	-1.309	1.132
H	-4.814	-3.221	0.388
H	1.696	5.444	-0.556
H	3.558	-1.086	-2.69
H	4.206	4.258	0.967
H	-1.7	2.406	2.532
H	-0.587	5.632	-1.014
H	-4.29	-1.122	-0.989
H	-3.987	1.36	3.246
H	-0.909	-4.3	-2.118
H	-1.849	-3.29	2.61
H	3.156	-3.918	1.31
H	2.549	0.465	-1.635
O	1.802	-2.956	-2.944
O	-3.515	-3.879	-1.553
O	-4.499	1.563	-1.229
O	0.839	3.202	2.512
O	6.103	2.404	0.369
O	0.96	2.427	-1.68
O	-3.262	5.254	-0.594
O	-4.332	-1.304	3.527
O	0.58	-4.31	2.157
O	5.247	-2.166	1.336
C	5.527	-3.214	2.273
H	5.097	-2.937	3.242
H	5.055	-4.149	1.948

C	0.253	-5.218	3.219
H	0.728	-4.813	4.12
H	-0.83	-5.225	3.39
C	-4.871	-0.609	4.653
H	-4.07	-0.072	5.18
H	-5.591	0.144	4.303
C	0.874	3.377	-2.752
H	-0.182	3.662	-2.808
H	1.451	4.274	-2.51
C	-3.125	6.113	-1.724
H	-2.122	6.056	-2.157
H	-3.817	5.724	-2.481
C	1.308	2.881	-4.164
C	7.048	-3.548	2.457
C	0.722	-6.705	3.064
C	-5.627	-1.489	5.705
C	-3.485	7.628	-1.525
O	0.494	-7.393	4.112
O	-5.96	-0.812	6.735
O	-3.445	8.279	-2.621
O	1.306	1.636	-4.388
O	7.324	-4.007	3.611
O	7.817	-3.43	1.472
O	1.247	-7.081	1.991
O	-5.87	-2.695	5.471
O	-3.786	8.065	-0.389
O	1.564	3.806	-4.984
C	2.432	-3.748	-3.962
H	3.51	-3.534	-3.967
H	2.306	-4.814	-3.725
C	-3.453	-4.846	-2.61
H	-3.122	-4.355	-3.534
H	-2.706	-5.61	-2.359
C	-5.404	1.111	-2.244
H	-4.957	1.428	-3.191
H	-5.463	0.016	-2.26
C	0.667	3.554	3.891
H	-0.397	3.529	4.157
H	1.167	2.762	4.459

C	6.682	3.648	0.768
H	6.216	4.464	0.196
H	6.473	3.833	1.829
C	-6.874	1.658	-2.203
C	1.251	4.922	4.382
C	8.223	3.788	0.53
C	1.882	-3.476	-5.389
C	-4.783	-5.618	-2.913
O	-4.699	-6.303	-3.985
O	2.364	-4.251	-6.269
O	8.732	4.741	1.209
O	1.083	5.095	5.634
O	-7.492	1.459	-3.3
O	1.81	5.691	3.566
O	-7.309	2.187	-1.155
O	-5.745	-5.554	-2.114
O	1.063	-2.534	-5.543
O	8.799	3.059	-0.311
K	0.464	-0.646	-3.611

WP5-K – TS_2b

C	1.425	-4.316	1.164
C	2.641	-3.645	0.954
C	2.918	-2.906	-0.213
C	1.941	-2.929	-1.226
C	0.665	-3.438	-0.934
C	0.357	-4.105	0.258
C	4.242	-2.159	-0.346
H	5	-2.704	0.235
H	4.563	-2.172	-1.391
C	-1.048	-4.682	0.498
H	-1.062	-5.164	1.477
H	-1.221	-5.467	-0.253
C	4.216	-0.695	0.124
C	4.611	0.356	-0.718
C	3.797	-0.336	1.418
C	4.355	1.686	-0.361
C	3.694	1.002	1.834

C	3.834	2.045	0.885
C	-2.216	-3.7	0.432
C	-2.659	-3.021	1.583
C	-2.945	-3.489	-0.752
C	-3.608	-1.993	1.454
C	-4.026	-2.591	-0.835
C	-4.285	-1.727	0.254
C	-5.285	-0.566	0.17
H	-5.903	-0.567	1.075
C	3.392	3.472	1.173
H	3.472	3.672	2.244
C	1.208	4.841	1.293
C	-0.149	4.984	0.942
C	-0.76	4.222	-0.069
C	0.056	3.318	-0.773
C	1.363	3.075	-0.33
C	1.958	3.792	0.715
C	-2.221	4.435	-0.461
H	-2.603	5.286	0.123
H	-2.27	4.729	-1.514
C	-3.16	3.239	-0.276
C	-3.927	2.727	-1.346
C	-3.323	2.628	0.974
C	-4.554	1.476	-1.196
C	-4.15	1.508	1.169
C	-4.646	0.819	0.045
H	-5.938	-0.737	-0.691
H	4.084	4.17	0.678
H	3.435	-3.734	1.688
H	4.547	2.458	-1.105
H	-0.761	5.72	1.455
H	1.953	2.314	-0.824
H	-4.996	0.991	-2.061
H	-2.781	3.04	1.821
H	-2.674	-4.072	-1.628
H	-3.841	-1.366	2.308
H	-0.12	-3.332	-1.677
H	3.516	-1.128	2.104
O	1.191	-5.181	2.198

O	-4.868	-2.528	-1.936
O	-4.014	3.493	-2.492
O	1.866	5.694	2.174
O	5.212	0.109	-1.957
O	3.368	1.371	3.11
O	-0.509	2.709	-1.853
O	-4.454	1.018	2.427
O	-2.083	-3.36	2.787
O	2.271	-2.518	-2.521
C	1.768	-3.404	-3.55
H	1.665	-4.408	-3.129
H	0.767	-3.068	-3.856
C	-2.875	-3.408	3.98
H	-2.223	-3.044	4.78
H	-3.725	-2.719	3.912
C	-4.74	1.944	3.483
H	-4.092	1.681	4.326
H	-4.48	2.962	3.174
C	3.782	0.599	4.244
H	3.017	0.786	5.002
H	3.763	-0.474	4.025
C	0.283	1.892	-2.708
H	0.841	1.146	-2.129
H	-0.449	1.345	-3.31
C	5.179	0.941	4.87
C	2.63	-3.513	-4.842
C	-3.439	-4.804	4.419
C	-6.215	1.997	4.014
C	1.25	2.618	-3.697
O	-3.866	-4.796	5.622
O	-6.32	2.736	5.05
O	1.989	1.793	-4.333
O	6.002	1.613	4.208
O	2.349	-4.53	-5.541
O	3.463	-2.606	-5.108
O	-3.454	-5.754	3.603
O	-7.124	1.377	3.417
O	1.203	3.858	-3.82
O	5.32	0.448	6.036

C	2.166	-5.493	3.191
H	2.84	-4.649	3.371
H	1.584	-5.633	4.109
C	-4.968	-3.69	-2.76
H	-4.179	-3.676	-3.525
H	-4.806	-4.586	-2.144
C	-5.113	3.288	-3.378
H	-4.845	2.544	-4.139
H	-5.968	2.886	-2.815
C	1.19	6.881	2.575
H	0.691	7.33	1.702
H	0.405	6.641	3.306
C	6.646	0.138	-1.933
H	6.978	1.013	-1.357
H	7.02	-0.757	-1.414
C	-5.634	4.574	-4.108
C	2.085	8.011	3.184
C	7.294	0.208	-3.345
C	3.023	-6.794	3.001
C	-6.337	-3.903	-3.489
O	-6.282	-4.862	-4.33
O	3.746	-7.044	4.021
O	8.567	0.199	-3.31
O	1.389	8.914	3.759
O	-6.387	4.3 -5.1	
O	3.328	7.995	3.026
O	-5.349	5.711	-3.66
O	-7.334	-3.206	-3.19
O	2.925	-7.462	1.946
O	6.549	0.276	-4.353
K	3.637	-0.202	-4.046

WP5-NH_4 – *pS*

C	0.129	-4.327	-1.648
C	1.502	-4.017	-1.646
C	2.186	-3.626	-0.48
C	1.439	-3.593	0.71
C	0.052	-3.758	0.678

C	-0.645	-4.078	-0.493
C	3.683	-3.302	-0.514
H	4.161	-3.754	0.362
H	4.117	-3.758	-1.411
C	-2.166	-4.235	-0.471
H	-2.426	-4.843	0.405
H	-2.465	-4.798	-1.359
C	4.031	-1.817	-0.525
C	3.984	-1.075	-1.719
C	4.453	-1.15	0.636
C	4.083	0.324	-1.656
C	4.758	0.223	0.647
C	4.431	1.007	-0.481
C	-2.977	-2.937	-0.408
C	-3.105	-2.191	0.775
C	-3.687	-2.473	-1.532
C	-3.765	-0.957	0.768
C	-4.471	-1.304	-1.5
C	-4.433	-0.465	-0.361
C	-5.128	0.901	-0.322
H	-5.895	0.888	0.462
C	4.433	2.53	-0.442
H	5.155	2.858	0.316
C	2.645	4.357	-0.746
C	1.346	4.839	-0.491
C	0.486	4.245	0.445
C	0.981	3.142	1.171
C	2.215	2.578	0.817
C	3.067	3.159	-0.134
C	-0.895	4.841	0.737
H	-0.927	5.116	1.8
H	-0.997	5.757	0.144
C	-2.105	3.952	0.459
C	-2.621	3.789	-0.844
C	-2.784	3.308	1.504
C	-3.56	2.77	-1.08
C	-3.883	2.461	1.279
C	-4.179	2.06	-0.037
H	-5.631	1.056	-1.28

H	4.779	2.925	-1.401
H	2.068	-4.073	-2.571
H	3.884	0.911	-2.549
H	0.978	5.704	-1.034
H	2.552	1.667	1.298
H	-3.835	2.518	-2.101
H	-2.429	3.459	2.519
H	-3.629	-3.056	-2.446
H	-3.781	-0.373	1.685
H	-0.513	-3.671	1.601
H	4.543	-1.723	1.554
O	-0.514	-4.883	-2.735
O	-5.301	-0.927	-2.539
O	-2.158	4.642	-1.826
O	3.539	5.004	-1.595
O	3.788	-1.761	-2.901
O	5.35	0.853	1.724
O	0.21	2.72	2.223
O	-4.672	1.97	2.305
O	-2.613	-2.697	1.983
O	2.054	-3.426	1.954
C	2.445	-4.685	2.53
H	1.632	-5.411	2.383
H	3.331	-5.069	2.007
C	-3.643	-3.393	2.707
H	-4.452	-2.689	2.949
H	-4.071	-4.175	2.064
C	-4.959	2.846	3.406
H	-4.353	2.542	4.268
H	-4.66	3.868	3.143
C	6.255	0.084	2.529
H	5.711	-0.356	3.375
H	6.653	-0.745	1.931
C	0.635	1.619	3.019
H	0.359	0.685	2.515
H	1.726	1.63	3.139
C	7.487	0.865	3.101
C	2.747	-4.645	4.052
C	-3.176	-4.055	4.027

C	-6.457	2.914	3.86
C	0.053	1.652	4.46
O	-4.043	-4.832	4.528
O	-6.612	3.664	4.881
O	0.027	0.503	5.018
O	7.667	2.07	2.814
O	3.395	-5.656	4.459
O	2.302	-3.689	4.739
O	-2.042	-3.772	4.495
O	-7.343	2.294	3.228
O	-0.243	2.75	4.974
O	8.224	0.119	3.827
C	0.243	-5.679	-3.654
H	0.554	-5.06	-4.505
H	1.156	-6.038	-3.16
C	-5.708	-1.924	-3.479
H	-4.922	-2.077	-4.23
H	-5.845	-2.881	-2.956
C	-3.006	4.946	-2.937
H	-2.635	4.417	-3.823
H	-4.023	4.585	-2.738
C	3.372	6.413	-1.768
H	2.491	6.616	-2.397
H	3.183	6.878	-0.792
C	4.521	-1.361	-4.066
H	3.791	-1.176	-4.863
H	5.049	-0.42	-3.876
C	-3.152	6.466	-3.295
C	4.575	7.156	-2.435
C	5.583	-2.375	-4.614
C	-0.494	-6.948	-4.201
C	-7.047	-1.625	-4.233
O	-7.278	-2.483	-5.148
O	-1.509	-7.385	-3.61
O	5.909	-3.371	-3.929
O	4.484	8.423	-2.314
O	-3.663	6.641	-4.449
O	5.463	6.512	-3.04
O	-2.836	7.339	-2.454

O	-7.766	-0.659	-3.89
O	0.097	-7.451	-5.212
O	6.036	-2.024	-5.753
N	0.122	-2.048	3.907
H	-0.75	-2.598	4.088
H	0.224	-1.95	2.897
H	0.08	-1.081	4.335
H	0.956	-2.592	4.234

WP5-NH4 – TS_1

C	-1.708	-4.153	1.56
C	-2.822	-3.326	1.802
C	-3.405	-2.519	0.808
C	-2.857	-2.618	-0.486
C	-1.671	-3.329	-0.688
C	-1.036	-4.062	0.322
C	-4.597	-1.619	1.159
H	-5.461	-1.896	0.542
H	-4.864	-1.8 2.206	
C	0.28	-4.785	0.031
H	0.153	-5.353	-0.9
H	0.46	-5.508	0.831
C	-4.365	-0.122	0.999
C	-3.518	0.563	1.88
C	-5.012	0.638	0.009
C	-3.113	1.871	1.572
C	-4.761	2.008	-0.166
C	-3.678	2.601	0.521
C	1.516	-3.89	-0.118
C	1.81	-3.19	-1.3
C	2.453	-3.786	0.926
C	2.88	-2.288	-1.341
C	3.621	-3.006	0.823
C	3.791	-2.143	-0.286
C	4.949	-1.144	-0.382
H	5.477	-1.299	-1.328
C	-3.132	3.967	0.107
H	-3.573	4.208	-0.867

C	-0.865	3.196	-0.782
C	0.536	3.27	-0.769
C	1.231	4.287	-0.098
C	0.469	5.3	0.529
C	-0.924	5.135	0.637
C	-1.613	4.084	0.013
C	2.767	4.323	-0.09
H	3.108	4.923	-0.948
H	3.09	4.858	0.806
C	3.462	2.968	-0.14
C	3.679	2.21	1.032
C	3.935	2.435	-1.349
C	4.124	0.885	0.911
C	4.539	1.167	-1.442
C	4.525	0.324	-0.312
H	5.652	-1.363	0.427
H	-3.491	4.741	0.802
H	-3.243	-3.277	2.801
H	-2.308	2.321	2.149
H	1.118	2.53	-1.307
H	-1.509	5.844	1.216
H	4.176	0.249	1.79
H	3.826	3.037	-2.246
H	2.264	-4.348	1.836
H	3.027	-1.704	-2.247
H	-1.242	-3.364	-1.682
H	-5.726	0.14	-0.639
O	-1.238	-5.072	2.48
O	4.638	-3.052	1.754
O	3.367	2.818	2.222
O	-1.61	2.299	-1.485
O	-3.04	-0.103	2.994
O	-5.508	2.821	-1.001
O	1.14	6.427	0.993
O	5.155	0.707	-2.596
O	1.086	-3.426	-2.478
O	-3.438	-2.015	-1.594
C	-4.706	-2.548	-2.003
H	-4.999	-3.361	-1.329

H	-5.456	-1.752	-1.916
C	1.708	-4.47	-3.253
H	2.776	-4.236	-3.366
H	1.639	-5.418	-2.702
C	5.646	1.686	-3.517
H	4.84	1.994	-4.197
H	5.961	2.579	-2.96
C	-6.811	2.381	-1.38
H	-6.744	1.703	-2.241
H	-7.258	1.811	-0.552
C	0.355	7.507	1.486
H	-0.504	7.671	0.816
H	-0.051	7.256	2.476
C	-7.831	3.515	-1.737
C	-4.78	-3.089	-3.459
C	1.135	-4.694	-4.677
C	6.869	1.252	-4.392
C	1.069	8.896	1.592
O	1.606	-5.73	-5.236
O	7.183	2.151	-5.242
O	0.424	9.711	2.335
O	-7.545	4.719	-1.536
O	-5.882	-3.657	-3.721
O	-3.807	-2.911	-4.237
O	0.322	-3.866	-5.164
O	7.437	0.153	-4.199
O	2.105	9.13	0.929
O	-8.927	3.045	-2.19
C	-2.15	-5.578	3.46
H	-2.105	-4.957	4.363
H	-3.175	-5.512	3.07
C	4.738	-4.198	2.606
H	4.129	-4.045	3.505
H	4.336	-5.076	2.083
C	3.942	2.409	3.467
H	3.142	2.531	4.203
H	4.211	1.347	3.455
C	-1.059	1.34	-2.379
H	-0.07	1.001	-2.046

H	-1.742	0.488	-2.306
C	-3.31	0.49	4.272
H	-2.467	0.204	4.911
H	-3.305	1.584	4.19
C	5.189	3.209	3.984
C	-0.973	1.781	-3.871
C	-4.624	0.071	5.016
C	-1.931	-7.072	3.875
C	6.191	-4.581	3.049
O	6.192	-5.437	3.993
O	-1.197	-7.817	3.186
O	-5.405	-0.754	4.486
O	-0.846	0.793	-4.676
O	5.413	3.003	5.221
O	-1.016	2.991	-4.162
O	5.841	3.922	3.188
O	7.179	-4.1 2.448	
O	-2.614	-7.387	4.905
O	-4.741	0.645	6.148
N	-1.204	-1.832	-3.917
H	-0.586	-2.519	-4.406
H	-0.921	-1.841	-2.937
H	-1.08	-0.836	-4.267
H	-2.201	-2.148	-3.966

WP5- NH_4 – TS_2a

C	-0.875	-3.593	1.55
C	-2.167	-3.054	1.536
C	-3.006	-3.097	0.413
C	-2.541	-3.864	-0.686
C	-1.202	-4.299	-0.711
C	-0.333	-4.161	0.387
C	-4.348	-2.361	0.402
H	-4.955	-2.725	-0.428
H	-4.899	-2.617	1.32
C	1.1 -4.705	0.318	
H	1.131	-5.489	-0.448
H	1.338	-5.169	1.281

C	-4.249	-0.825	0.311
C	-5.157	-0.043	-0.447
C	-3.29	-0.115	1.037
C	-4.879	1.322	-0.652
C	-3.132	1.272	0.974
C	-3.852	2.011	0.022
C	2.212	-3.712	-0.008
C	2.431	-3.281	-1.327
C	3.114	-3.265	0.973
C	3.338	-2.237	-1.564
C	4.166	-2.376	0.685
C	4.195	-1.735	-0.575
C	5.093	-0.537	-0.866
H	5.536	-0.639	-1.862
C	-3.608	3.493	-0.3
H	-4.137	3.716	-1.234
C	-1.41	3.487	-1.572
C	-0.02	3.681	-1.573
C	0.626	4.486	-0.629
C	-0.189	5.19	0.285
C	-1.538	4.824	0.412
C	-2.161	3.931	-0.476
C	2.15	4.59	-0.585
H	2.504	5.232	-1.406
H	2.413	5.107	0.344
C	2.916	3.271	-0.658
C	2.736	2.274	0.322
C	3.878	3.041	-1.652
C	3.439	1.066	0.217
C	4.635	1.855	-1.723
C	4.371	0.817	-0.802
H	5.922	-0.54	-0.149
H	-4.057	4.119	0.485
H	-2.53	-2.601	2.455
H	-5.458	1.88	-1.381
H	0.584	3.168	-2.318
H	-2.129	5.231	1.226
H	3.284	0.288	0.956
H	4.031	3.822	-2.39

H	2.991	-3.633	1.987
H	3.386	-1.793	-2.555
H	-0.795	-4.753	-1.608
H	-2.633	-0.64	1.715
O	-0.147	-3.523	2.738
O	5.182	-2.09	1.574
O	1.863	2.59	1.317
O	-2.056	2.794	-2.577
O	-6.288	-0.666	-0.945
O	-2.209	1.845	1.835
O	0.401	6.204	1.021
O	5.647	1.656	-2.662
O	1.691	-3.864	-2.338
O	-3.452	-4.155	-1.672
C	-3.175	-5.156	-2.66
H	-2.875	-4.657	-3.588
H	-2.342	-5.79	-2.333
C	2.362	-4.266	-3.539
H	1.675	-4.032	-4.36
H	3.27	-3.672	-3.688
C	6.073	2.788	-3.413
H	5.278	3.105	-4.105
H	6.255	3.633	-2.731
C	-2.762	2.669	2.868
H	-1.944	3.332	3.172
H	-3.567	3.293	2.468
C	-0.451	7.155	1.656
H	-1.337	7.326	1.028
H	-0.807	6.759	2.618
C	-3.296	1.961	4.148
C	-4.356	-6.143	-2.961
C	2.764	-5.775	-3.676
C	7.371	2.604	-4.266
C	0.179	8.564	1.92
O	3.248	-6.037	-4.827
O	7.684	3.679	-4.882
O	-0.581	9.301	2.632
O	-2.92	0.783	4.402
O	-4.331	-6.604	-4.148

O	-5.134	-6.46	-2.03
O	2.596	-6.556	-2.713
O	7.977	1.509	-4.29
O	1.284	8.875	1.416
O	-4.049	2.692	4.855
C	-0.251	-4.671	3.592
H	-0.995	-5.365	3.182
H	0.718	-5.19	3.599
C	5.498	-3.065	2.574
H	4.921	-2.857	3.484
H	5.202	-4.06	2.216
C	1.409	1.623	2.261
H	0.35	1.855	2.41
C	-1.852	3.235	-3.925
H	-0.823	3.59	-4.061
H	-1.974	2.338	-4.543
C	-7.319	0.152	-1.5
H	-7.384	1.09	-0.929
H	-7.069	0.417	-2.535
C	-2.812	4.333	-4.498
C	-8.765	-0.448	-1.476
C	-0.636	-4.381	5.072
C	7.01	-3.169	2.971
O	7.192	-3.989	3.931
O	-0.693	-5.433	5.781
O	-9.548	0.152	-2.286
O	-2.567	4.593	-5.722
O	-3.695	4.835	-3.765
O	7.88	-2.521	2.345
O	-0.848	-3.197	5.435
O	-9.059	-1.355	-0.663
N	-0.609	-0.724	3.93
H	-0.662	-1.598	4.498
H	-0.528	-1.023	2.958
H	-1.474	-0.138	4.042
H	0.253	-0.139	4.163
C	2.118	1.638	3.645
O	1.584	0.822	4.476
O	3.077	2.405	3.851

H	1.458	0.613	1.837

WP5- NH_4 – TS_2b

C	0.162	-4.742	-1.153
C	1.535	-4.431	-1.197
C	2.182	-3.686	-0.193
C	1.395	-3.301	0.907
C	0.015	-3.51	0.892
C	-0.649	-4.176	-0.144
C	3.68	-3.378	-0.31
H	4.213	-3.882	0.509
H	4.042	-3.805	-1.252
C	-2.171	-4.353	-0.119
H	-2.424	-4.98	0.749
H	-2.463	-4.921	-1.006
C	4.076	-1.908	-0.287
C	3.819	-1.083	-1.388
C	4.757	-1.334	0.801
C	3.98	0.307	-1.259
C	5.101	0.027	0.843
C	4.568	0.899	-0.136
C	-3.014	-3.075	-0.03
C	-3.039	-2.297	1.132
C	-3.851	-2.684	-1.088
C	-3.756	-1.097	1.177
C	-4.649	-1.523	-1.007
C	-4.528	-0.637	0.093
C	-5.18	0.746	0.105
H	-5.685	0.923	1.056
C	4.582	2.424	0.029
H	4.989	2.646	1.02
C	2.932	4.105	-1.045
C	1.615	4.575	-1.23
C	0.553	4.08	-0.456
C	0.828	3.137	0.537
C	2.137	2.654	0.705
C	3.206	3.071	-0.125
C	-0.852	4.679	-0.642

H	-0.964	5.494	0.09
H	-0.909	5.131	-1.638
C	-2.047	3.752	-0.487
C	-2.49	2.957	-1.557
C	-2.8	3.707	0.7
C	-3.469	1.978	-1.325
C	-3.918	2.868	0.861
C	-4.174	1.881	-0.117
H	-5.941	0.766	-0.681
H	5.256	2.88	-0.704
H	2.127	-4.749	-2.049
H	3.605	0.953	-2.05
H	1.401	5.315	-1.993
H	-3.698	1.263	-2.111
H	-2.506	4.363	1.513
H	-3.876	-3.291	-1.987
H	-0.541	-3.173	1.755
H	5.014	-1.977	1.635
O	-0.448	-5.596	-2.056
O	-5.579	-1.194	-1.972
O	-1.893	3.13	-2.789
O	4.01	4.628	-1.749
O	3.335	-1.658	-2.551
O	5.931	0.567	1.811
O	2.495	1.762	1.665
O	-4.793	2.973	1.927
O	-3.671	-0.344	2.332
O	1.945	-2.673	2.016
C	2.615	-3.536	2.942
H	3.061	-4.386	2.411
H	3.43	-2.94	3.369
C	-4.767	-0.475	3.256
H	-4.967	0.533	3.633
H	-5.661	-0.817	2.722
C	-4.825	4.21	2.644
H	-4.07	4.194	3.442
H	-4.563	5.03	1.962
C	6.732	-0.333	2.575
H	6.133	-0.778	3.381

H	7.068	-1.158	1.929
C	1.536	1.114	2.492
H	0.602	0.95	1.94
H	1.967	0.128	2.694
C	8.025	0.27	3.221
C	1.776	-4.095	4.128
C	-4.528	-1.415	4.472
C	-6.2	4.602	3.282
C	1.222	1.8	3.853
O	-5.581	-1.685	5.122
O	-6.1	5.641	4.017
O	0.454	1.092	4.595
O	8.375	1.449	2.983
O	2.362	-5.018	4.768
O	0.651	-3.585	4.378
O	-3.353	-1.793	4.736
O	-7.236	3.952	3.016
O	1.719	2.91	4.126
O	8.634	-0.587	3.946
C	0.364	-6.551	-2.742
H	0.805	-6.094	-3.638
H	1.197	-6.853	-2.091
C	-6.03	-2.218	-2.865
H	-5.332	-2.309	-3.707
H	-6.035	-3.182	-2.338
C	-2.725	3.237	-3.952
H	-2.259	2.617	-4.725
H	-3.719	2.821	-3.748
C	3.828	5.887	-2.398
H	3.189	5.766	-3.285
H	3.304	6.573	-1.719
C	4.075	-1.434	-3.758
H	3.33	-1.331	-4.554
H	4.621	-0.484	-3.694
C	-2.935	4.67	-4.552
C	5.133	6.61	-2.869
C	5.092	-2.538	-4.208
C	-0.371	-7.864	-3.173
C	-7.473	-2.026	-3.44

O	-7.726	-2.861	-4.369
O	-1.517	-8.121	-2.735
O	5.322	-3.518	-3.461
O	4.883	7.765	-3.353
O	-3.483	4.636	-5.703
O	6.251	6.057	-2.761
O	-2.594	5.679	-3.891
O	-8.241	-1.168	-2.949
O	0.349	-8.588	-3.937
O	5.598	-2.29	-5.352
N	-0.772	-1.204	3.741
H	-1.743	-1.331	4.116
H	-0.851	-1.087	2.73
H	-0.301	-0.323	4.116
H	-0.184	-2.049	3.933
H	0.03	2.799	1.188
H	-2.528	-2.611	2.035
